# The *PLA* Gene Family in Tomato: Identification, Phylogeny, and Functional Characterization

**DOI:** 10.3390/genes16020130

**Published:** 2025-01-23

**Authors:** Zixing Li, Zhuping Yao, Meiying Ruan, Rongqing Wang, Qingjing Ye, Hongjian Wan, Guozhi Zhou, Yuan Cheng, Shangjing Guo, Chaochao Liu, Chenxu Liu

**Affiliations:** 1 College of Biotechnology, Jiangsu University of Science and Technology, Zhenjiang 212018, China; 221211801105@stu.just.edu.cn; 2 State Key Laboratory for Managing Biotic and Chemical Threats to the Quality and Safety of Agro-Products, Institute of Vegetables, Zhejiang Academy of Agricultural Sciences, Hangzhou 310021, China; yaozp@zaas.ac.cn (Z.Y.); ruanmy@zaas.ac.cn (M.R.); wangrq@zaas.ac.cn (R.W.); yeqj@zaas.ac.cn (Q.Y.); hjwan@zaas.ac.cn (H.W.); zhougz@zaas.ac.cn (G.Z.); chengyuan@zaas.ac.cn (Y.C.); 3 Zhejiang Xianghu Laboratory, Hangzhou 311258, China; 4 College of Horticulture, Qingdao Agricultural University, Qingdao 266109, China; guoshangjing@qau.edu.cn

**Keywords:** phospholipase A, genome-wide identification, phylogenetic analysis, cold stress, tomato

## Abstract

Background: Phospholipase A (PLA) enzymes catalyze the hydrolysis of glycerophospholipids, releasing free fatty acids and lysophospholipids that play vital roles in plant growth, development, and stress responses. Methods: This study identified and analyzed *SlPLA* genes through bioinformatics and further explored the function of *PLA* genes under cold stress through virus-induced gene silencing (VIGS) experiments. Results: This study systematically characterized the *SlPLA* gene family in tomato, identifying 80 genes distributed across 12 chromosomes. Phylogenetic analysis categorized these genes into three groups: pPLA, PLA1, and PLA2. Conserved motifs and gene structure analysis revealed distinct patterns, with some genes lacking untranslated regions (UTRs), which suggests functional diversification. Promoter analysis indicated that *SlPLA* genes are regulated by light, hormones, and stress-related elements, particularly cold stress. RNA-seq data and qRT-PCR results indicated the differential expression of *SlPLA* genes across various tissues in tomato cultivars (Heinz and Micro-Tom). Under cold stress, certain *SlPLA* genes, especially *SlPLA1-2*, were up-regulated, suggesting their involvement in cold tolerance. Silencing *SlPLA1-2* resulted in increased membrane damage, elevated malondialdehyde (MDA) levels, higher electrolyte leakage, and a lower expression of cold-responsive genes within the ICE1-CBF-COR pathway and jasmonic acid (JA) biosynthesis. Conclusions: This study discovered 80 *SlPLA* genes in tomato across 12 chromosomes, categorizing them into pPLA, PLA1, and PLA2 via phylogenetic analysis. The qRT-PCR analysis identified that *SlPLA1-2* was strongly induced by cold stress, and further experiments regarding genetics and physiology revealed that *SlPLA1-2* boosts the cold tolerance of tomato by affecting the CBF signaling pathway and JA biosynthesis, offering insights for future stress-resilience breeding.

## 1. Introduction

Phospholipids, as a class of lipid molecules containing phosphates, are one of the primary components of cell membranes [1]. Phospholipases (PLs) catalyze the hydrolysis of phospholipids, yielding a variety of lipid molecules, including free fatty acids (FFAs), phosphatidic acids (PAs), diacylglycerol (DAG), and lysophospholipids [2]. These hydrolysis products are essential to the signaling pathways that regulate plant growth, development, and responses to stress [3]. PLs can be classified into three main families according to their different hydrolysis sites, including phospholipase A (PLA), phospholipase C (PLC), and phospholipase D (PLD) [4]. PLA, serving as ubiquitous phospholipid hydrolases found in various organisms, liberates free fatty acids and lysophospholipids through the cleavage of the *sn-1* or *sn-2* positions of glycerophospholipids. Based on distinct functional domains, PLAs could be categorized into three groups: patatin-like phospholipase A (pPLA), phospholipase A1 (PLA1), and phospholipase A2 (PLA2) [5]. pPLAs hydrolyzes glycerolipids to produce free fatty acids and monoacyl derivatives [6]. PLA1s catalyze the hydrolysis of phospholipids at the *sn-1* position, specifically targeting phosphatidylcholine (PC) and PA [7]. Free fatty acids and hemolytic phospholipids are produced by PLA2s hydrolyzing glycerophospholipids at the *sn-2* position [8,9].

A growing body of evidence has indicated that PLA was critical for cell growth, seed germination, root development, and pollen maturation [10]. In *Arabidopsis*, the over-expression of *pPLAIIIβ* resulted in reduced length of leaves, petioles, hypocotyls, main root, and root hairs, as well as a decrease in cellulose content compared with wild-type plants. This indicated that *pPLAIIIβ* was essential for root growth and the proliferation of plant cells [10]. The up-regulation of *pPLAIIIα* in *Arabidopsis* has been demonstrated to decrease lignin levels in secondary cell walls, causing inhibited plant growth and development, reduced leaf surface area, shortened seed length, and decreased root hair density [11]. The silencing of *GhpPLA23* and *GhpPLA44* genes decreased the size of petals, stigmas, and anthers, as well as resulted in a significant reduction in the vitality of pollen. This ultimately affected the reproductive and developmental processes of upland cotton [12]. In addition, *PLA* genes play a role in regulating plant responses to biotic and abiotic stress factors. In cotton (*Gossypium barbadense*), *GbPLA1-32-*silenced plants exhibited a disruption in the stability of leaf membrane structure compared to wild types under salt stress [13]. Under salt and drought stress, the knockout of *pPLAIIIγ* resulted in decreased germination rates of *Arabidopsis* seeds and significant reductions in main root length and dry weight. Compared with the control, the growth of knockout plants was more sensitive to salt and drought stress [14]. Compared with wild-type plants, *AtPLAI* knocked-out plants exhibited increased lesion size, maceration of leaves, and leaf necrosis under *Botrytis cinerea* (*B. cinerea*) infection [15]. This result indicates that plant *PLA*s also participate in plant biotic stress responses.

Recently, PL was reported to be crucial for sexual reproduction in numerous plants [16]. Phospholipase *NOT-LIKE-DAD* (*NLD*), also referred to as *MTL* or *ZmPLA1*, which encoded a membrane-localized phospholipase, was essential for the process of fertilization (Gilles et al., 2021). Previous studies have demonstrated that the mutation of the maize *MTL* gene triggers haploid induction (HI) [17]. Furthermore, in rice and wheat, the knockout of *MTL/ZmPLA1/NLD* homologous genes also leads to HI [18].

Tomato, an important vegetable crop in the *Solanaceae* family, has been widely cultivated around the world [19]. However, studies on the *SlPLA* gene family of tomato are currently absent, and the functions of its members in growth, development, and stress response remain unclear. In this study, a detailed investigation into the gene structure, chromosomal positions, evolutionary relationships, and expression of the *SlPLA* gene family was undertaken. In addition, the role of *SlPLA1-2* in tomato plants exposed to low temperatures was identified using the VIGS method. This study aims to further our knowledge of the evolutionary connections between *SlPLA* genes in tomato and their possible function in combating abiotic stress.

## 2. Materials and Methods

### 2.1. Plant Materials and Treatments

Tomato seeds of the varieties ‘Micro-Tom’ and ‘Ailsa Craig’ (AC) were used in this study. Germinated seeds were planted in a 3:1 mixture of peat and vermiculite. Plants were grown in a greenhouse under a 12 h photoperiod at 25 °C with 70% humidity. Samples from root, stem, leaf, mature pollen, pistil, and fruit were collected from ‘Micro-Tom’ to study the expression levels of *SlPLA* genes in different tissues, with four biological replicates for each.

Cold treatment was applied to ‘AC’ plants at the five-leaf stage by placing them in a 4 °C incubator. Samples of leaves were collected at 0, 3, 6, 12, and 24 h and then quickly frozen in liquid nitrogen and kept at −80 °C for RNA extraction.

### 2.2. Identification and Phylogenetic Analysis of PLA Genes

The *PLA* gene family members were identified using the Hidden Markov Model (HMM) and Blastp approaches [20]. A Blastp search of the NCBI tomato genome database (https://www.ncbi.nlm.nih.gov/, accessed on 3 November 2023) was conducted using AtPLAs protein sequences as the query [21]. The HMM search was also performed on the tomato genome database using conserved functional domains from the Pfam database (http://pfam.xfam.org, accessed on November 10, 2023). Redundant entries from both methods were filtered to yield the final *SlPLA* gene family members, which were annotated on the Softberry website (http://www.softberry.com/, 10 December 2023). To examine the evolutionary relationships, PLA protein sequences from *Arabidopsis* and maize were also included. Genome data of these species were downloaded from the NCBI, and ClustalX2 software was used for multiple sequence alignment. A phylogenetic tree was generated utilizing MEGA11.0 software, employing the Maximum Likelihood (ML) method and incorporating 2000 bootstrap replications for statistical support [22].

### 2.3. Characterization of PLA Proteins, Chromosomal Localization, and Synteny Analysis in Tomato

The ExPASy tool was used to predict the molecular weights and isoelectric points of SlPLA proteins (https://web.expasy.org/protparam/, accessed on 4 November 2023) [23,24]. Subcellular localization predictions were obtained using Cell-PLoc2.0 (http://www.csbio.sjtu.edu.cn/bioinf/plant-multi/, accessed on accessed on 13 November 2023) [20]. The locations of SlPLA genes on chromosomes were identified with the ‘Show Genes on Chromosome’ tool in TBtools software [25], and synteny analysis of *SlPLA* genes was conducted using the One step MCScanX model of TBtools v1.110 [26].

### 2.4. Conserved Motifs, Gene Structure, and Cis-Acting Elements of SlPLA Genes

The MEME suite was used to identify conserved motifs in SlPLA proteins (https://meme-suite.org/meme/tools/meme, accessed on accessed on 25 November 2023) [27], specifying a total of 10 motifs that vary from 6 to 200 residues, with visualization in TBtools (version 2.080). Researchers analyzed the *SlPLA* family’s gene structure using GSDS online (https://gsds.gao-lab.org/, accessed on 28 November 2023).

In TBtools, the 2000 bp upstream promoter region for each *PLA* member was extracted, and PlantCARE was used to predict *cis*-acting elements (http://bioinformatics.psb.ugent.be/webtools/plantcare/html/, accessed on accessed on 4 February 2024) [28,29]. Visualization of *cis*-acting structure for each *SlPLA* member was conducted in TBtools [30].

### 2.5. Tissue-Specific, Cold Stress-Responsive Expression Analysis

RNA-seq data obtained from the Tomato Functional Genomics Database (TFGD; http://ted.bti.cornell.edu/, accessed on 4 April 2024) and Molecular Plant Online [31] were used to examine the expression patterns of the *SlPLA* genes. Expression data across different tissues (root, stem, leaf, bud, flower, and fruit) were compared between two tomato varieties, Heinz and Micro-Tom. The expression data of *SlPLA* genes under cold stress were obtained from published studies [32].

### 2.6. RNA Isolation and qRT-PCR Analysis

RNA extraction was performed by HiPure Plant RNA Plus Kit (Megan, R4150, Guangzhou, China), followed by reverse transcription into cDNA for fluorescence quantitative identification [33]. The initial cDNA strand was generated from 1 μg of total RNA utilizing HiScript II Q RT Super Mix for qPCR (+gDNA wiper) (Nanjing Vazyme Biotech, Nanjing, China) in accordance with the manufacturer’s guidelines. Gene-specific primers for qRT-PCR were designed using NCBI’s Primer-BLAST tool. Primer details are provided in Table S1. qRT-PCR was conducted on a CFX96 Real-Time System (Bio-Rad, Boston, MA, United States) using ChamQ Universal SYBR qPCR Master Mix reagent (Vazyme, Nanjing, China). The qRT-PCR parameters were 95 °C for 30 s, followed by 40 cycles of 95 °C for 3 s, 60 °C for 30 s, and final steps of 95 °C for 15 s, 60 °C for 60 s, and 95 °C for 15 s. Each reaction included four replicates, with data processed using the 2^−ΔCT^ or 2^−ΔΔCT^ method.

### 2.7. Virus-Induced Gene Silencing Assay

A 300 bp CDS fragment for silencing was obtained from the Sol Genomics Network (https://solgenomics.net/, accessed on 18 April 2024). The VIGS primers were designed using the Vazyme CETool (https://crm.vazyme.com/cetool/simple.html, accessed on 18 April 2024), and viral vector construction and plant injection followed methods from previous research [34]. The primer sequence was F: GTGAGTAAGGTTACCGAATTCATGGATGGTCTTTGTTTGACAGG. R: CGTGAGCTCGGTACCGGATCCGCAAAATCTTCAATACCCAATTCTC.

### 2.8. Cold Stress Tolerance Assay

Four-week-old tomato seedlings were subjected to cold stress at 4 °C, while control plants were kept in normal conditions. For RNA extraction and qRT-PCR analysis, leaf samples were gathered at 0 h, 3 h, 6 h, and 12 h after treatment. After 7 days of cold exposure, ion leakage assays were conducted to measure relative electrolyte leakage (REL) in the leaves. For REL measurement, 0.1 g of leaf tissue was placed in 10 mL of deionized water, shaken for 2 h, and the initial conductivity (R1) was recorded. Samples were then heated to 95 °C for 15 min, allowed to cool to room temperature, and then their final conductivity (R2) was measured. REL was calculated as the ratio of R1 to R2. The maximum photochemical efficiency of PSII (*Fv/Fm*) was evaluated using the PlantExplorer XS (Wageningen, The Netherlands). MDA content was determined using the MDA kit (Grace, G0109W, Suzhou, China).

### 2.9. Statistical Analysis

All experiments were conducted in a completely randomized design with three replicates. One-way ANOVA was used for statistical analyses, followed by Tukey’s multiple range test (*p* < 0.05) in SPSS (version 16.0).

## 3. Results

### 3.1. Identification and Chromosome Localization of SlPLA Genes

A Blastp search revealed 81 possible *PLA* genes within the entire genome of the tomato (Table S2). The amino acid sequence of Solyc04g079230 exhibits consistency with that of Solyc04g079210, as confirmed by annotations on the Softberry website. Of these, 80 *SlPLA* genes were named based on group classification and chromosomal location (Table 1). The amino acid (aa) sequences of SlPLAs ranged from 78 aa (SlPLA1-41) to 1348 aa (SlpPLA4), with molecular weight (MW) spanning 8.57 kDa (SlPLA1-41) to 149.43 kDa (SlpPLA4) and isoelectric points (pI) from 4.86 (SlpPLA16) to 9.43 (SlPLA2-1). Subcellular localization analysis revealed that 51 genes were located within chloroplasts, 19 genes in vacuoles, 1 gene in Golgi apparatus, and the remaining in diverse cellular compartments such as cell membranes, cell walls, nucleus, and cytoplasm.

**Figure 1:**
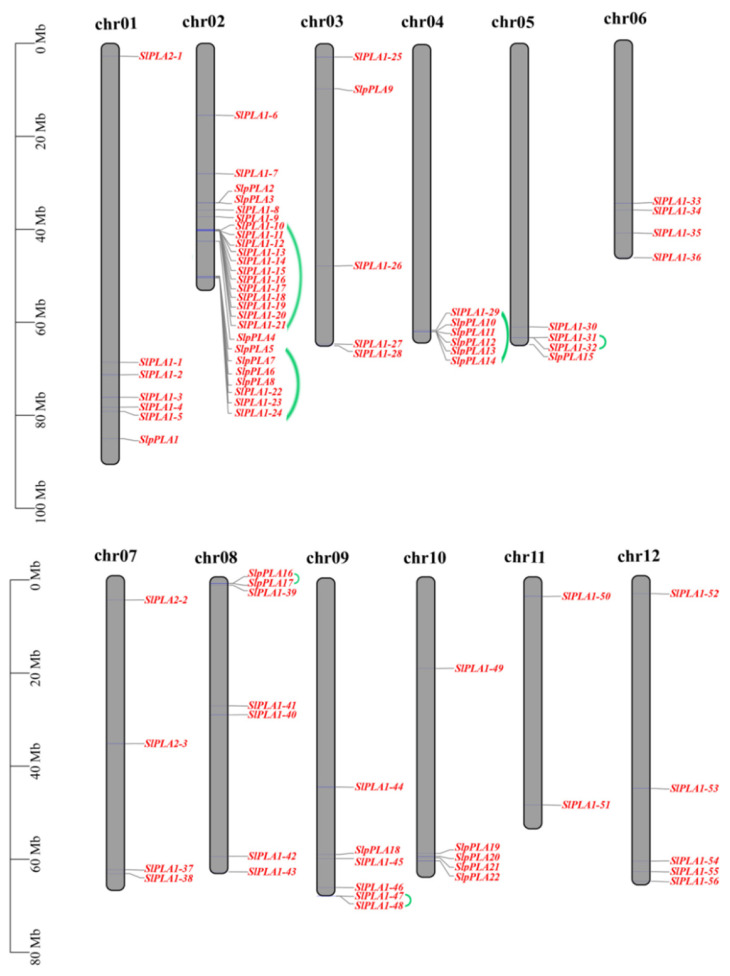
Chromosomal positioning of *PLA* genes in tomato. The gray bar structure signifies the chromosome. The chromosome number is located above the chromosome and displayed in black font. The *PLA* genes were represented in red font and located on the right side of the chromosome, with tandem repeat genes connected with a green line. The scale bar located on the left side of the figure represents the length of chromosomes in millions of bases (Mb).

The *SlPLA* genes were found to be distributed unevenly across 12 chromosomes according to chromosomal mapping (Figure 1). Among these, chromosome 2 contained the largest number (26) of *SlPLA* members, followed by chromosomes 1 and 8 with 7 each, while chromosome 11 had only 2 genes. The expansion of gene family members was largely driven by tandem repeat events [35]. In this study, seven tandem segments were found on chromosomes 2, 4, 5, 8, and 9 of tomato (Figure 1).

### 3.2. Phylogenetic Analysis of PLA Gene Family

**Figure 2:**
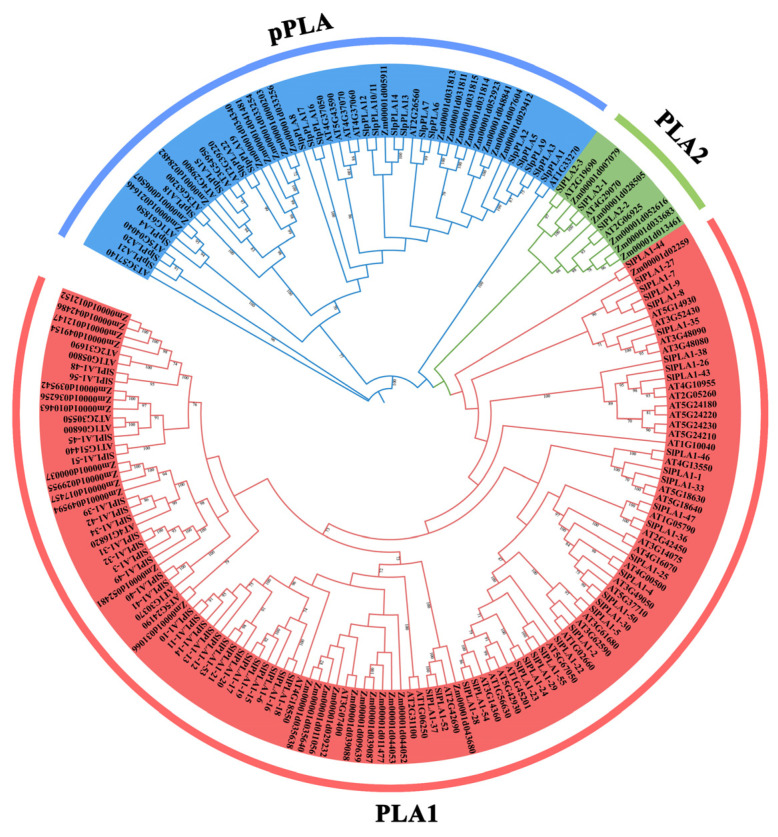
Phylogenetic tree of PLAs from tomato, *Arabidopsis*, and maize. Multiple sequence alignment was performed on the amino acid sequences of 185 PLA in *Arabidopsis*, rice, and tomato. The phylogenetic tree was constructed with MEGA11.0 software using the ML method with 2000 bootstrap replications. Red/green/blue represent PLA1/PLA2/pPLA group, respectively.

A phylogenetic tree was created using selected *PLA* genes from *Arabidopsis*, maize, and tomato to further examine their phylogenetic relationships (Figure 2). The results revealed that these *PLA* genes could be categorized into three groups: pPLA, PLA1, and PLA2, which is consistent with prior research [13]. The pPLA group consisted of 52 *PLA* members, with 21 from tomato; the PLA2 group contained 11 *PLA* members, while the PLA1 comprised 122 members, including 56 from tomato. Moreover, the analysis showed that the *SlPLA* genes exhibited a closer evolutionary relationship with the *Arabidopsis* members within each group.

### 3.3. Analysis of Conserved Motifs and Gene Structure in SlPLA Genes

**Figure 3:**
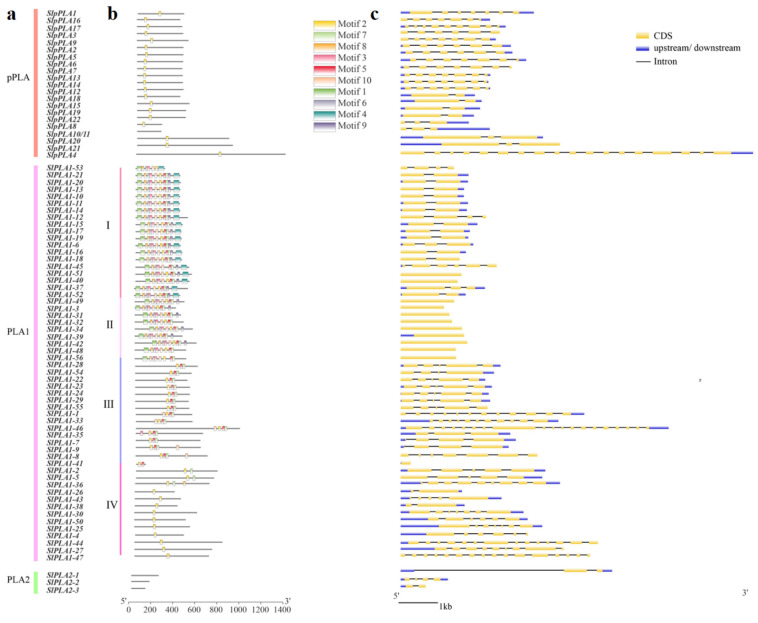
Gene structure and motif analysis of *PLA* gene family in tomato. (**a**) Classification of 80 *SlPLA* genes based on phylogenetic tree (**b**) Ten conserved motifs of the SlPLA family, predicted by Multiple Em for Motif Elicitation (MEME). The different colors represent different amino acids. The bigger letters represent a more conserved sequence. (**c**) The gene structure of the *SlPLA* genes. The yellow box represents the CDS regions, the UTR is denoted by the blue box, and the intron regions are represented by the black line.

Using the MEME tool, we identified 10 conserved motifs (motifs 1–10) among the 80 SlPLA proteins (Figure 3a), with lengths ranging from 16 to 50 amino acids (Table 2). Four proteins (SlPLA2-1, SlPLA2-2, SlPLA2-3, and SlpPLA10/11) lacked conserved motifs. The motif 2 was ubiquitous in all SlPLA proteins except for SlPLA2-1, SlPLA2-2, SlPLA2-3, SlPLA10/11, and SlPLA1-45 (Figure 3b). In the SlpPLA group, only motif 2 was consistently present. Based on these conserved motifs, group PLA1 was divided into four subgroups (I-IV) [13]. Subgroup I members had seven conserved motifs, including the subgroup-specific motif 4. Subgroup II shared high similarity with subgroup I but lacked motif 4. In subgroup III, all members, except SlPLA1-41, contained motifs 2, 5, and 7. Three members (SlPLA1-2, SlPLA1-5, SlPLA1-36) in subgroup IV contained motif 7. None of the SlPLA2 members detected conserved motifs.

Intron-exon configurations revealed that *SlPLA* genes had 0-18 introns, with similar intron-exon arrangements within each subgroup (Figure 3c). Most subgroup I members (57%) within the PLA1 group contained a single intron, while subgroup II genes were intron less, and subgroup III had mostly 3-4 introns. *SlPLA1-46* in subgroup III contained the highest intron count (18). In addition, we observed that some members contained the 5′UTR or 3′UTR or both of them. Among them, 3 genes had only the 5′UTR region, 11 genes had only the 3′UTR, 41 genes had both the 5′UTR and 3′UTR, and 15 genes lacked any UTR.

### 3.4. Collinearity and Duplication Analysis of SlPLA Genes

**Figure 4:**
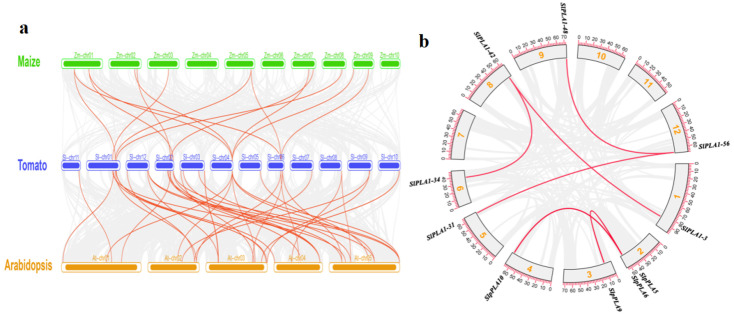
Analysis of collinearity and homogeneity of *SlPLA* genes. (**a**) Collinearity analysis was performed on three species of *PLA* genes, including *Arabidopsis*, maize, and tomato. Red lines in the background indicate collinear regions in tomato and other plants, while gray line highlight collinear gene pairs between *PLA* members. (**b**) The duplication genes in the *SlPLA* gene family. The red line indicates all collinear genes in the genome, while the gray box symbolizes chromosomes, and the yellow numbers inside the gray box denote chromosome numbers.

In order to analyze the evolutionary relationships of *PLA* genes from tomato, *Arabidopsis*, and maize, a comparative analysis was performed by TBtools (Figure 4a). Results indicated 37 orthologous pairs between tomato and *Arabidopsis*, and 14 pairs of *PLA* genes showed synteny with maize. Furthermore, we also imported the tomato genome data and the genome annotation file into the TBtools plugin One step MCScanX, which allowed us to obtain information on gene duplication of the tomato genome. Within the 80 *SlPLA* genes, a total of six duplicate gene pairs were identified (Figure 4b). There were two pairs (*SlpPLA5/SlpPLA9*, *SlpPLA6/SlpPLA10*) of tandem repeat genes in the pPLA group. The PLA1 group had four pairs of tandem repeat genes, containing *SlPLA1-42/SlPLA1-34*, *SlPLA1-42/SlPLA1-3*, *SlPLA1-48/SlPLA1-56*, *SlPLA1-31/SlPLA1-48*.

### 3.5. Promoter Cis-Element Analysis of SlPLA Genes

**Figure 5:**
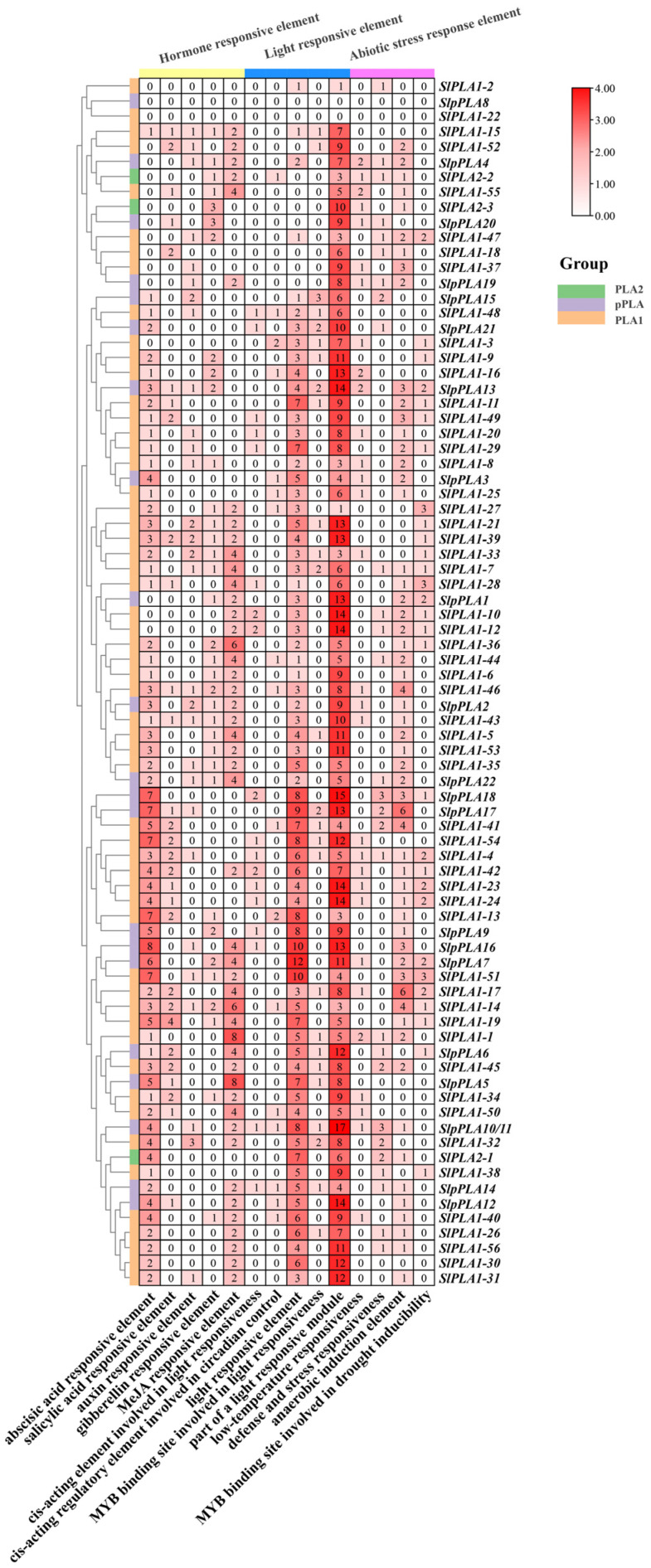
Analysis of *cis*-acting elements in the promoter region of the *SlPLA* gene family. At the top of the heat map, various colors are used to distinguish the overall categories of *cis*-acting elements, such as ABA, MeJA, and IAA, all of which are classified as hormone-response elements. Each box contains the number of *cis*-acting elements. The intensity of the box’s color is directly proportional to the number of components it encompasses.

To investigate the regulatory potential of SlPLA family members, we analyzed 2000 bp upstream promoter regions for *cis*-element composition using the PlantCARE database (Figure 5). The identified *cis*-elements were classified into three main categories: light response, hormone response, and abiotic stress response element. The findings indicated that, with the exception of *SlpPLA8* and *SlPLA1-22*, light response elements and hormone response elements were present in the promoter regions of all *SlPLA* family members. Light response elements were the most abundant (including conserved DNA module involved in light responsiveness, part of a light-responsive module, *cis*-acting regulatory element involved in light responsiveness, part of a module for light response, and MYB binding site involved in light responsiveness). Additionally, 73 genes were found to contain abiotic stress-responsive elements (e.g., drought, low temperature, anaerobic, and defense elements). In addition, we found many hormone-specific *cis*-elements, including abscisic acid (ABA), methyl jasmonate (MeJA), gibberellin (GA), salicylic acid (SA), and auxin (IAA).

### 3.6. Expression Analysis of SlPLA Genes in Different Organs Based on RNA-Seq Data

**Figure 6:**
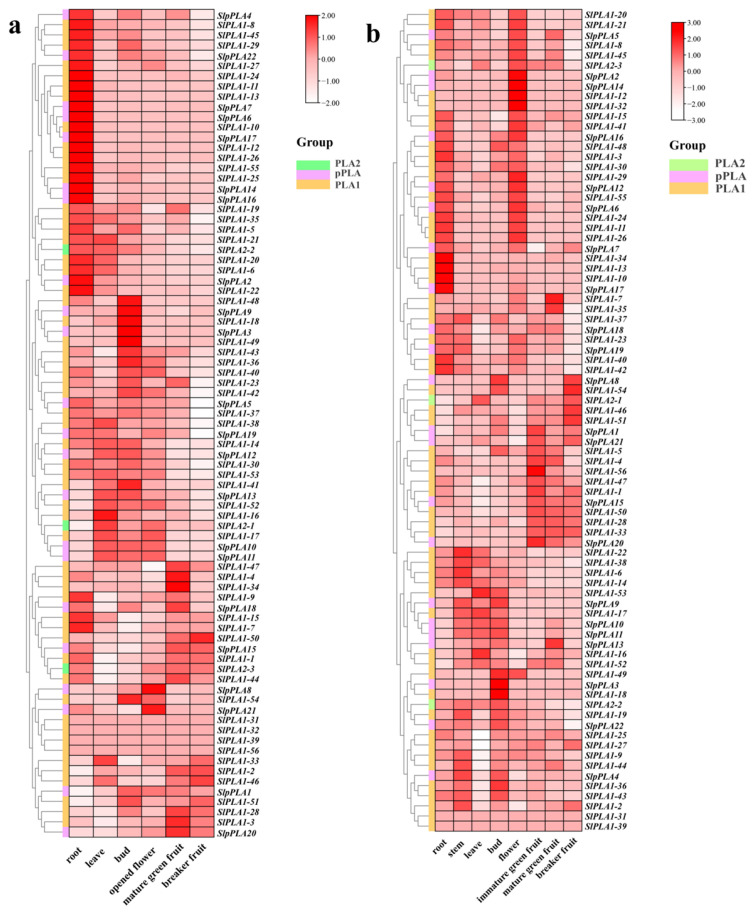
RNA-seq analysis of *SlPLA* gene expression across various tomato tissues. (**a**) RNA seq data of *PLAs* in different tissues of Heinz tomato. (**b**) RNA seq data of *PLAs* in different tissues of Micro-Tom tomato. Heat maps were displayed using red, pink, and white colors to indicate high, medium, and low expression levels, respectively.

We analyzed *SlPLA* gene expression profiles in various tomato organs of the Heinz and Micro-Tom cultivars (Figure 6). In Heinz, 28% of *SlPLA* genes were highly expressed in roots, 8% in leaves, 11% in flower buds, and 7% in fruits (Figure 6a). There were only two highly expressed genes (*SlpPLA21* and *SlpPLA8*) in the opened flower, and both were classified within the pPLA group. In breaker fruit, only the *SlPLA1-50* gene was highly expressed. Compared to Heinz, Micro-Tom had more *SlPLA* genes that were highly expressed in flowers (25%) (Figure 6b). In Micro-Tom, high expression of the *SlPLA* genes accounted for 12.5% in the root, 5% in the stem, 6% in flower bud, 6% in immature green fruit, and 6% in breaker fruit. We observed that during the developmental stage of Micro-Tom tomato fruit (immature green fruit, mature green fruit, and breaker fruit), the expression levels of two genes (*SlPLA1-46* and *SlPLA1-51*) were significantly up-regulated, while the expression levels of five genes (*SlPLA1-4*, *SlPLA1-5*, *SlPLA1-47*, *SlpPLA20*, and *SlPLA1-56*) were significantly down-regulated. Furthermore, with the exception of *SlpPLA20*, all other genes were classified within the PLA1 group. When the expression profiles of the *SlPLA* genes between the two tomato genotypes were compared, *SlPLA1-16* was highly expressed in leaves of both varieties, and five genes (*SlPLA1-10*, *SlPLA1-11*, *SlPLA1-13*, *SlPLA1-26*, and *SlpPLA17*) were highly expressed in their root.

### 3.7. Expression Analysis of the SlPLA Genes in Different Organs Using qRT-PCR

**Figure 7:**
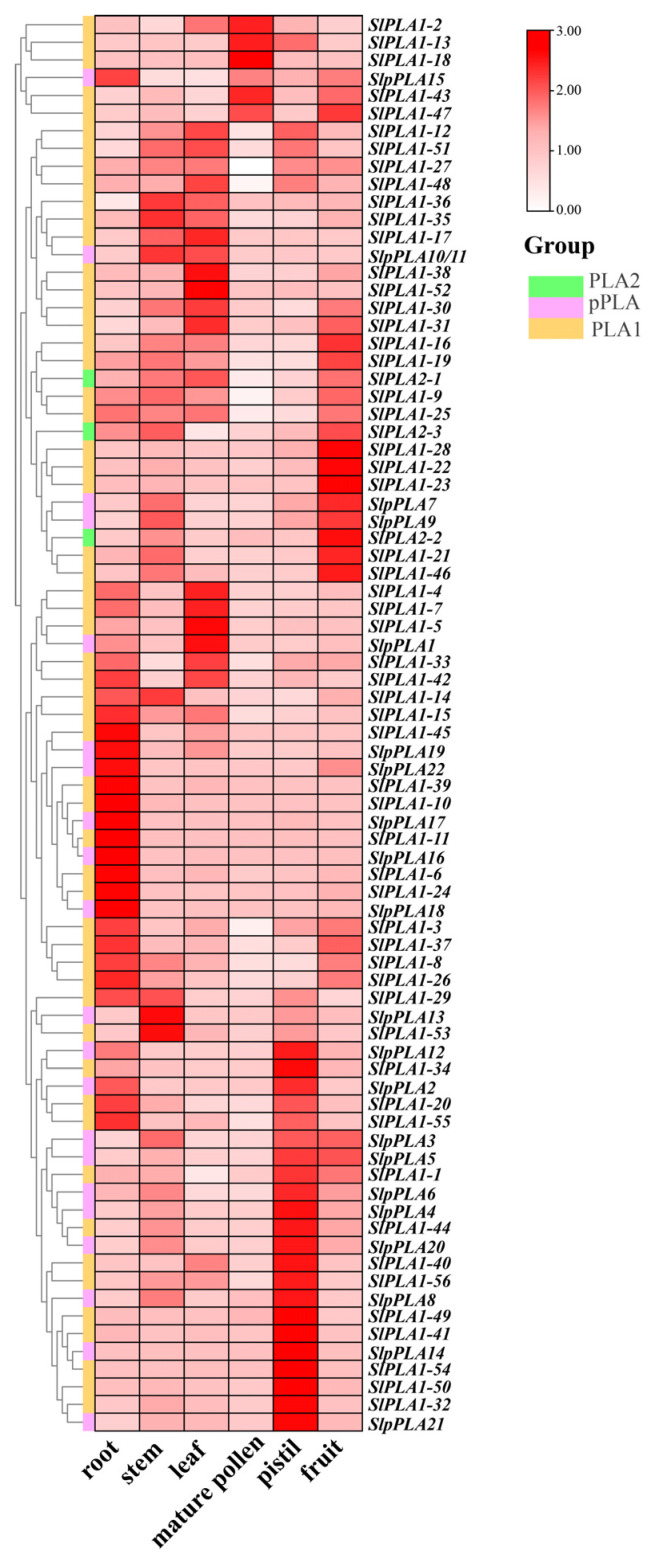
qRT-PCR was employed to assess the relative expression levels of 80 genes within the *SlPLA* family across various tissues of the Micro-Tom. Heat maps were presented using a color scheme of red, pink, and white to represent high, medium, and low expression levels, respectively.

We further examined the expression levels of *SlPLA* genes in six organs of tomato (Micro-Tom) by qRT-PCR method, including root, stem, leave, mature pollen, pistil, and fruit. Results revealed diverse expression patterns of all *SlPLA* genes across the different organs (Figure 7). *SlPLA* genes had similar gene structures and physicochemical properties across groups, but their expression varied in different organs. We found that *SlPLA1-3*, *SlPLA1-6*, *SlPLA1-10*, *SlPLA1-11*, *SlPLA1-24*, *SlPLA1-39*, *SlPLA1-45*, *SlPLA1-55*, *SlpPLA15*, *SlpPLA16*, *SlpPLA17*, *SlpPLA18*, *SlpPLA19*, and *SlpPLA22* were highly expressed in root. There were eight *SlPLA* genes highly expressed in the leaves, namely *SlPLA1-4*, *SlPLA1-5*, *SlpPLA1*, *SlPLA1-7*, *SlPLA1-17*, *SlPLA1-38*, *SlPLA1-48*, and *SlPLA1-52*. Approximately 10% of *SlPLA* genes were highly expressed in fruit. Only four genes, e.g., *SlPLA2-1*, *SlpPLA13*, *SlPLA1-35*, and *SlPLA1-53*, were highly expressed in stems. In addition, approximately a quarter of the *SlPLA* genes were highly expressed in the pistil. It is worth noting that *SlPLA1-2*, *SlPLA1-13*, *SlPLA1-18*, and *SlPLA1-43* were highly expressed in mature pollen, and all of these genes belonged to the *PLA1* group.

### 3.8. Expression Analysis of SlPLA Genes Under Cold Stress

**Figure 8:**
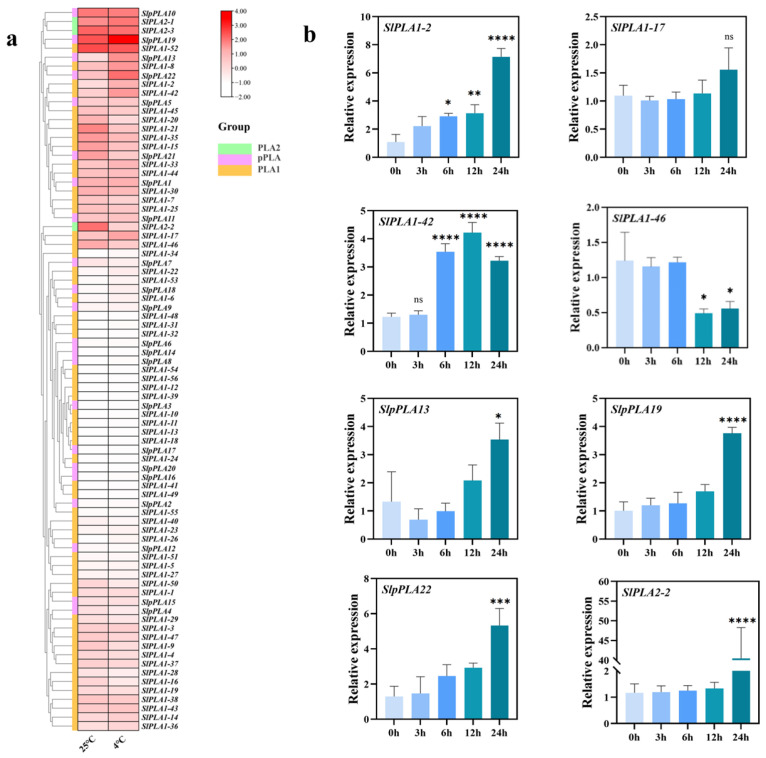
Expression analysis of *SlPLA* genes under low-temperature stress. (**a**) Expression profile of *SlPLA* genes under low-temperature conditions. Heat maps were presented in red/pink/white colors that represent high/medium/low expression, respectively. (**b**) qRT-PCR analysis of representative *SlPLA* genes under low-temperature conditions (0 h, 3 h, 6 h, 12 h, and 24 h). We used 0 h as the control for cold stress treatment, with its relative expression set at 1. The “ns” indicates that the difference between the groups is not statistically significant. * *p* < 0.05, ** *p* < 0.01, *** *p* < 0.001, **** *p* < 0.0001.

To investigate the expression of *SlPLA* genes in response to cold stress, we analyzed tomato RNA-seq data under cold treatment, and the results showed that five genes (*SlPLA1-2*, *SlPLA1-17*, *SlpPLA13*, *SlPLA1-42*, and *SlpPLA19*) were up-regulated and three genes (*SlPLA2-2*, *SlPLA1-46*, and *SlpPLA22*) were down-regulated (Figure 8a). In addition, qRT-PCR analysis was performed on these differentially expressed genes (Figure 8b). Under chilling stress, the up-regulation of *SlPLA1-2*, *SlpPLA13*, *SlPLA1-42*, and *SlpPLA19* gene expression levels were consistent with RNA-seq results. The down-regulation in the expression of the *SlPLA1-46* gene aligns with the RNA-seq results, while the *SlPLA2-2* and *SlpPLA22* were significantly up-regulated, which is inconsistent with RNA-seq data. The relative expression of *SlPLA1-17* showed no significant difference under cold treatment compared with normal temperature treatment, which was also inconsistent with RNA-seq data. This inconsistency may be attributed to variations in sampling times, methods of plant cultivation, and other related factors, which can lead to differing outcomes.

### 3.9. SlPLA1-2 Positively Regulated Tomato Cold Tolerance

**Figure 9:**
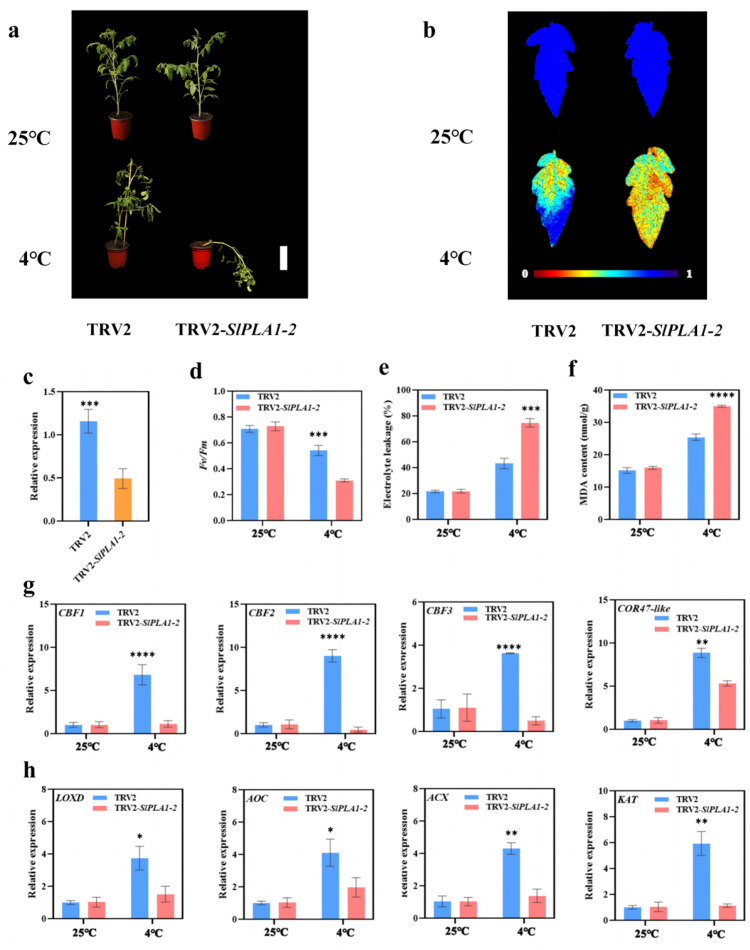
Silencing *SlPLA1-2* with VIGS heightened the tomato plants’ sensitivity to cold stress. (**a**) Phenotypes of TRV2 and TRV2-*SlPLA1-2* tomato plant after exposure to 25 or 4 °C for 7 days. (**b**) *Fv*/*Fm* in TRV2 and TRV2-*SlPLA1-2* tomato leaves after exposure to 25 or 4 °C for 7 days. The false color code depicted at the bottom of the image ranges from 0 (black) to 1.0 (purple), representing the level of damage in the leaves. (**c**) qRT-PCR analysis of the relative expression levels of *SlPLA1-2* in the silenced plant (TRV2-*SlPLA1-2*) and control (TRV2) plant. (**d**) The Photosynthetic Capacity (*Fv/Fm*) in TRV2-*SlPLA1-2* tomato leaves after exposure to 25 or 4 °C for 7 days. (**e**) The relative electrolyte leakage in TRV2, and TRV2-*SlPLA1-2* tomato leaves after exposure to 25 or 4 °C for 7 days. (**f**) The MDA content in TRV2-*SlPLA1-2* tomato leaves after exposure to 25 or 4 °C for 7 days. (**g**) Relative transcript levels of *SlCBF1*, *SlCBF2*, *SlCBF3*, and *COR47-like* in TRV2 and TRV2-*SlPLA1-2* tomato plant after exposure to 25 or 4 °C. (**h**) Relative transcript levels of *LOXD*, *AOC*, *ACX*, and *KAT* in TRV2, and TRV2-*SlPLA1-2* tomato plant after exposure to 25 or 4 °C. * p < 0.05, ** p < 0.01, *** p < 0.001, **** p < 0.0001.

To further investigate the function of *SlPLA1-2*, a gene from the PLA1 group that is highly expressed in response to cold stress in tomato, we used gene silencing technology to reduce endogenous *SlPLA1-2* expression. Silencing efficiency was verified to ensure successful suppression of *SlPLA1-2* expression (Figure 9c). Under normal conditions (25 °C), TRV2-*SlPLA1-2* plants displayed no noticeable phenotypic differences compared to TRV2 control plants. However, following cold treatment (4 °C), the *SlPLA1-2*-silenced plants exhibited more severe wilting symptoms and a significant reduction in the maximum photo quantum efficiency of PSII (*Fv*/*Fm*) compared to the control. Both relative electrolyte leakage and malondialdehyde (MDA) content were markedly higher in *SlPLA1-2*-silenced plants than in TRV2 plants (Figure 9b,d–f), indicating that *SlPLA1-2* may play a positive role in enhancing tomato cold tolerance.

To further explore the mechanisms by which *SlPLA1-2* contributes to cold tolerance, we examined the expression levels of downstream genes essential to tomato’s cold response. *CRT/DRE-binding factors* (*CBFs*) and *Cold-regulated* (*COR*) genes, which are key players in cold response, were significantly induced in TRV2 plants under cold treatment compared to normal temperatures. However, in *SlPLA1-2*-silenced plants, *CBF* expression was significantly lower under cold stress compared to controls (Figure 9g). Previous studies have suggested that impaired *PLA1* genes may disrupt JA biosynthesis, so we also assessed the expression of JA biosynthesis-related genes (*LOXD*, *AOC*, *ACX*, and *KAT*) under both normal and cold conditions. These genes were significantly induced in TRV2 plants under cold stress, but in TRV2-*SlPLA1-2* plants, their expression levels were reduced relative to TRV2 under the same conditions (Figure 9h). This evidence suggests that *SlPLA1-2* might improve cold tolerance in tomato by modulating the CBF signaling pathway and boosting JA biosynthesis.

## 4. Discussion

### 4.1. Bioinformatic Analysis of PLA Genes in Tomato: Structure, Features, and Evolutionary Relationships

Phospholipase A is a crucial enzyme that facilitates the hydrolysis of phospholipids, leading to the production of hydrolysates that play a vital role in the growth and development of plants. In recent years, researchers have elucidated the characteristics of *PLA* genes and further enhanced our understanding of plant responses to stresses, including *Arabidopsis*, maize, and cotton [36]. However, the *PLA* gene family has not been characterized in tomato, hindering the understanding of the functional role of *SlPLA* genes. This study fills that gap by providing the first comprehensive analysis of the *PLA* genes in tomato, identifying 80 *SlPLA* genes in significantly larger families than in maize (47 members) and *Arabidopsis* (57 members), which suggests species-specific expansion in tomato. Our phylogenetic analysis categorized *PLA* genes into three primary groups: *pPLA*, *PLA1*, and *PLA2*, consistent with earlier studies [13]. This analysis also revealed that tomato *SlPLA* genes share close evolutionary relationships with those in other dicots, indicating evolutionary conservation along with distinct expansions in tomato [37]. Gene duplication appears to be a major driver of this family’s expansion in tomato, with both tandem and segmental duplications contributing to gene amplification [38,39]. Tandem duplications, defined as adjacent genes from the same subfamily within a chromosome and separated by fewer than ten genes [40], were evident across seven clusters on chromosomes 2, 4, 5, 8, and 9. Additionally, we identified six segmentally duplicated gene pairs, supporting the role of duplication events in the expansion of the *SlPLA* gene family in tomato [41].

Functional predictions of conserved motifs suggest these sequences may act as recognition sites or encode functional protein regions, providing insight into family members’ structural and functional diversity [42]. Interestingly, we observed variation in gene architecture, with certain *SlPLA* genes lacking UTRs, a feature also reported in *PLA* genes in sorghum [43]. This variability in UTR presence may indicate functional diversification within the family. Promoter analysis identified three main types of *cis*-acting elements associated with light response, hormone response, and abiotic stress response. Light-responsive elements were the most numerous, suggesting a strong regulatory link between *SlPLA* gene expression and light conditions. Additionally, many *SlPLA* promoters contained stress-responsive *cis*-elements related to drought, low temperature, and anaerobic conditions, reflecting a stress-resilient expression pattern similar to what has been reported in soybean [44]. The prevalence of these *cis*-elements implies that *SlPLA* genes expression may be finely tuned by various environmental factors, such as light intensity, hormone levels, and stress exposure, making these genes potentially significant targets for enhancing stress resilience in tomato [12].

### 4.2. Expression Analysis Indicates PLA Genes’ Roles in Tomato Development and Stress Response

RNA-seq data revealed that *SlPLA* genes accounted for 28% of high expression levels in Heinz tomato root and 20% in Micro-Tom tomato flowers, suggesting notable differential expression between these varieties. Additionally, qRT-PCR results identified 14 *SlPLA* genes with high expression levels in roots, 4 in stems, 8 in leaves, 18 in pistil, and 9 in fruit, highlighting the gene family’s diverse roles in growth and development across tissues. In Micro-Tom, four genes (*SlPLA2-1*, *SlpPLA13*, *SlPLA1-35*, and *SlPLA1-53*) were specifically expressed in pollen, suggesting potential involvement in pollen development or fertilization. This is consistent with findings in maize, where *ZmPLA1*, a pollen-specific gene, contributes to haploid production when inactivated [45]. Therefore, these pollen-specific genes in tomato may similarly play roles in HI. In addition, the expression patterns also aligned with findings from *cis*-element analysis. For instance, *SlpPLA4*, which contains the highest number of photo-responsive elements, is homologous to the phototactic *Arabidopsis* gene *AT1G61850* [46]. Meanwhile, *SlpPLA18*, showing strong phylogenetic similarity to *Arabidopsis pPLAIIIδ* (*AT3G63200*), was exclusively expressed in tomato roots. *Arabidopsis pPLAIIIδ* overexpression has been linked to reduced lignin and seed germination rates [11], suggesting that *SlpPLA18* may similarly contribute to root development and germination in tomato.

We further analyzed *SlPLA* gene expression under cold stress using RNA-seq data and qRT-PCR. RNA-seq data indicated that four genes (*SlPLA1-2*, *SlPLA1-17*, *SlpPLA13*, *SlPLA1-42*, and *SlpPLA19*) were significantly up-regulated, while three (*SlPLA2-2*, *SlPLA1-46*, and *SlpPLA22*) were down-regulated. However, qRT-PCR validation showed that three genes (*SlPLA1-17*, *SlPLA2-2*, and *SlpPLA22*) displayed different expression trends compared to RNA-seq, likely due to differences in sampling time points. *Cis*-element analysis of eight differentially expressed genes under chilling stress revealed that, except for *SlPLA1-2* and *SlpPLA22*, all contained low-temperature responsive elements. In addition, five of these genes (excluding *SlPLA1-2*, *SlPLA2-2*, and *SlpPLA19*) had ABA-responsive elements, and six had MeJA-responsive elements, except for *SlpPLA13* and *SlPLA1-2*. Both ABA and JA are typical phytohormones that enhance plant cold tolerance by activating the classical ICE1-CBF-CORs pathway, as well as other physiological and biochemistry processes [47]. Thus, variations in the number of *cis*-acting elements may play a significant role in modulating *SlPLA* gene expression under cold stress conditions.

### 4.3. The SlPLA1-2 Positively Regulated Tomato Cold Tolerance Through CBF Pathway and JA Biosynthesis Pathway

We conducted a VIGS assay on *SlPLA1-2* to explore the involvement of *PLA* genes in tomato cold tolerance. Following cold stress treatment, *SlPLA1-2*-silenced plants exhibited severe wilting phenotype, along with increased MDA content and electrolyte leakage, in contrast to TRV2 control plants. MDA, a byproduct of membrane lipid peroxidation, reflects the extent of stress-induced cell membrane damage, with higher levels indicating decreased stress tolerance [48]. Similarly, electrolyte leakage, which indicates membrane permeability, was significantly higher in the VIGS plants, suggesting substantial membrane damage in TRV2-*SlPLA1-2* lines [49]. In this study, the MDA content and electrolyte leakage in VIGS lines were significantly higher than in the control group after cold treatment. These results indicated that TRV2-*SlPLA1-2* suffered serious damage in the cell membrane. Damage to the cell membrane can impact numerous critical cellular processes, including carbon allocation, cell elongation, defense responses, seedling establishment, and overall plant growth. These processes were also associated with the release of membrane fatty acids and the storage of lipids within the plant system [3]. Lipid metabolism is critical for plant growth and defense against abiotic and biotic stress [50]. PLA enzymes are crucial in lipid metabolism as they break down phospholipids to produce free fatty acids and lysophosphatids, vital for maintaining membrane stability and responding to stress [51]. Thus, silencing *SlPLA1-2* likely impairs lipids metabolism, leading to reduced tolerance to cold stress.

Based on these findings, we propose that *SlPLA1-2* acts as a positive regulator of tomato cold tolerance [13]. The ICE1-CBF-COR pathway is a well-established signaling cascade involved in cold stress response [52]. Under cold stress conditions, plants with silenced *SlPLA1-2* exhibited significantly reduced expression of *SlCBFs* and *SlCOR47-like* genes compared to control, indicating that *SlPLA1-2* may enhance cold tolerance by modulating the CBF-COR pathway [53]. Additionally, the *AT1G02660* gene (*PLIP2*), homologous to *SlPLA1-2*, has been implicated in JA biosynthesis [54]. In *SlPLA1-2*-silenced plants, expression of JA biosynthesis genes (*LOXD*, *AOC*, *ACX*, and *KAT*) was significantly reduced under cold stress, suggesting that *SlPLA1-2* may positively regulate these genes to enhance JA synthesis, which in turn contributes to cold resistance. These results indicate that *SlPLA1-2* may enhance tomato cold tolerance by positively regulating the CBF signaling pathway and JA biosynthesis, positioning *SlPLA* genes as beneficial modulators of stress resilience in tomato. Nevertheless, the limitations of this study are primarily attributed to the insufficient availability of gene editing materials. Future research endeavors will aim to address and overcome these constraints.

Furthermore, under conditions of cold stress, analyses of RNA-seq and qRT-PCR data revealed the presence of four consistently expressed genes (*SlPLA1-42*, *SlPLA1-46*, *SlpPLA19*, and *SlpPLA22*), with the exception of *SlPLA1-2*. These genes may also exert regulatory influences in response to cold stress. Among these, the protein sequence of *SlPLA1-46* in the PLA1 group exhibits similarity to that of the *AT4G13550,* as demonstrated by evolutionary tree analysis. Previous studies have identified the *AT4G13550* gene as playing a significant role in lipid remodeling in response to abiotic stress [55]. And the *AT4G16820* gene, which encodes a protein sequence analogous to *SlPLA1-42*, has been identified as encoding an enzyme involved in the biosynthesis of JA [56]. In the pPLA group, the gene *AT2G39220*, which exhibits similarity to the *SlpPLA19* and *SlpPLA22* genes, has been identified as a participant in the biosynthetic pathway of JA [54]. Subsequent investigations will provide additional validation regarding the roles of genes that exhibit sensitivity to cold stress.

## 5. Conclusions

This study identified and characterized 80 *SlPLA* genes, offering the first comprehensive analysis of their structural features, evolutionary relationships, and functional roles. Phylogenetic analysis grouped *SlPLA* genes into pPLA, PLA1, and PLA2 across 12 chromosomes, with *cis*-elements linked to light, hormone, and stress responses. The expression profile and VIGS experiment demonstrated that *SlPLA1-2* enhances cold tolerance in tomato by activating the CBF signaling pathway and JA biosynthesis. These findings lay the groundwork for further investigation into the molecular mechanisms underlying tomato resistance to abiotic stress within the *PLA* family, as well as for future functional studies and potential crop improvement strategies targeting the *SlPLA* gene family.

## Figures and Tables

**Table 1:** *SlPLA* gene family members and related information.

Gene Rename	Gene ID	Molecular Weight /kda	Amino Acid	Isoelectric Points/PI	CDS/bp	Genomic Sequence/bp	Subcellular Localization
*SlPLA2-1*	*Solyc01g008780*	27.66	240	9.43	723	5204	chloroplast
*SlPLA1-1*	*Solyc01g067380*	56.59	503	6.63	1512	8886	chloroplast
*SlPLA1-2*	*Solyc01g079600*	81.04	731	6.16	2196	3758	chloroplast
*SlPLA1-3*	*Solyc01g090220*	41.25	365	6.26	1098	1098	chloroplast
*SlPLA1-4*	*Solyc01g094570*	49.39	434	5.99	1035	5247	chloroplast
*SlPLA1-5*	*Solyc01g095720*	79.22	700	6.08	2103	3579	nucleus
*SlpPLA1*	*Solyc01g104310*	44.8	412	5.67	1239	4033	chloroplast, vacuole
*SlPLA1-6*	*Solyc02g014470*	40.85	402	6.28	1029	1475	chloroplast, cell wall
*SlPLA1-7*	*Solyc02g032850*	65.26	578	8.04	1737	10,228	nucleus
*SlpPLA2*	*Solyc02g065090*	44.97	414	9.14	1245	2288	vacuole
*SlpPLA3*	*Solyc02g065100*	45.86	411	9.37	1236	1896	vacuole
*SlPLA1-8*	*Solyc02g067660*	72.98	645	8.76	1938	7483	chloroplast, nucleus
*SlPLA1-9*	*Solyc02g069400*	66.13	581	6.9	1746	3071	cell membrane, chloroplast
*SlPLA1-10*	*Solyc02g076990*	44.33	395	5.1	1188	1427	cytoplasm
*SlPLA1-11*	*Solyc02g077000*	44.5	394	5.68	1185	1530	cell wall
*SlPLA1-12*	*Solyc02g077010*	52.46	466	6.48	1401	3272	cell wall, chloroplast
*SlPLA1-13*	*Solyc02g077020*	45.27	398	5.92	1197	1436	chloroplast, cytoplasm
*SlPLA1-14*	*Solyc02g077030*	45.03	397	8.28	1194	1538	chloroplast, cytoplasm
*SlPLA1-15*	*Solyc02g077100*	47.78	419	6.58	1260	1961	chloroplast
*SlPLA1-16*	*Solyc02g077110*	47.17	417	6.63	1254	1492	cell wall, chloroplast
*SlPLA1-17*	*Solyc02g077140*	46.75	408	6.7	1227	1567	chloroplast
*SlPLA1-18*	*Solyc02g077150*	47.37	413	5.29	1242	1317	chloroplasts, cytoplasm
*SlPLA1-19*	*Solyc02g077160*	47.01	410	6.34	1233	1537	chloroplast
*SlPLA1-20*	*Solyc02g077420*	45.23	402	5.84	1209	1541	chloroplast
*SlPLA1-21*	*Solyc02g077430*	45.01	398	5.54	1197	1533	cell wall, cytoplasm
*SlpPLA4*	*Solyc02g080340*	149.43	1348	5.57	4047	8953	vacuole
*SlpPLA5*	*Solyc02g090490*	44.99	412	8.33	1239	2215	cell membrane, vacuole
*SlpPLA6*	*Solyc02g090630*	44.66	408	5.52	1227	2954	vacuole
*SlpPLA7*	*Solyc02g090640*	43.74	402	6.27	1209	1882	vacuole
*SlpPLA8*	*Solyc02g090660*	24.61	218	8.65	657	1337	vacuole
*SlPLA1-22*	*Solyc02g090920*	54.1	466	7.64	1401	2254	chloroplast
*SlPLA1-23*	*Solyc02g090930*	55.45	488	8.35	1467	4428	chloroplasts, cytoplasm
*SlPLA1-24*	*Solyc02g090940*	55.73	487	7.51	1464	3081	chloroplast, cytoplasm
*SlPLA1-25*	*Solyc03g025510*	56.56	498	8.02	1497	5363	chloroplast
*SlpPLA9*	*Solyc03g044710*	51.37	460	5.82	1383	7197	chloroplast, vacuole
*SlPLA1-26*	*Solyc03g083370*	40.32	354	9.16	1065	1898	chloroplast
*SlPLA1-27*	*Solyc03g122280*	76.53	697	5.21	2094	7773	nucleus
*SlPLA1-28*	*Solyc03g123750*	64.1	558	5.75	1677	3169	cytoplasm
*SlPLA1-29*	*Solyc04g078800*	55.61	477	7.59	1434	2766	chloroplasts, cytoplasm
*SlpPLA10/11*	*Solyc04g079210/Solyc04g079230*	23.35	212	5.33	639	1438	cell membrane, vacuole
*SlpPLA12*	*Solyc04g079240*	45.46	412	6.53	1239	3534	vacuole
*SlpPLA13*	*Solyc04g079250*	44.93	405	6.37	1218	2024	vacuole
*SlpPLA14*	*Solyc04g079260*	44.96	406	8.3	1221	2349	chloroplasts, vacuoles
*SlPLA1-30*	*Solyc05g051280*	63.74	562	6.58	1689	6566	chloroplast
*SlPLA1-31*	*Solyc05g053910*	46.23	412	5.42	1239	1239	cell wall, chloroplast
*SlPLA1-32*	*Solyc05g053920*	49.35	435	6.41	1308	1308	chloroplast, nucleus
*SlpPLA15*	*Solyc05g056030*	51.21	466	8	1401	4200	vacuole
*SlPLA1-33*	*Solyc06g054550*	56.29	506	6.73	1521	7825	chloroplast
*SlPLA1-34*	*Solyc06g060870*	48.49	518	8.53	1557	1557	nucleus
*SlPLA1-35*	*Solyc06g071280*	69.04	602	7.17	1809	3347	chloroplast
*SlPLA1-36*	*Solyc06g083920*	75	666	8.87	2001	5919	chloroplast
*SlPLA2-2*	*Solyc07g014730*	17.12	156	7.45	471	3553	cell membrane, nucleus
*SlPLA2-3*	*Solyc07g032220*	13.06	119	5.51	360	604	Golgi apparatus
*SlPLA1-37*	*Solyc07g055160*	54.79	481	5.76	1446	2930	chloroplast
*SlPLA1-38*	*Solyc07g056250*	42.23	379	9.03	1140	1999	nucleus
*SlpPLA16*	*Solyc08g006850*	42.23	385	4.86	1158	6136	vacuole
*SlpPLA17*	*Solyc08g006860*	44.81	404	4.96	1215	4063	vacuole
*SlPLA1-39*	*Solyc08g007225*	47.34	425	8.18	1278	1615	chloroplasts
*SlPLA1-40*	*Solyc08g022240*	55.31	482	8.28	1449	1449	chloroplast
*SlPLA1-41*	*Solyc08g023410*	8.57	78	9.05	237	237	chloroplast
*SlPLA1-42*	*Solyc08g078090*	61.08	552	8.88	1659	1659	chloroplast
*SlPLA1-43*	*Solyc08g082450*	45.67	411	6.79	1236	4749	chloroplast
*SlPLA1-44*	*Solyc09g056350*	89	791	8.56	2376	10,320	chloroplast
*SlpPLA18*	*Solyc09g065240*	41.36	383	9	1152	1967	chloroplasts, vacuoles
*SlPLA1-45*	*Solyc09g065890*	55.44	480	6.56	1443	3501	chloroplast
*SlPLA1-46*	*Solyc09g091050*	104.51	936	5.36	2811	10,488	chloroplast, cytoplasm
*SlPLA1-47*	*Solyc09g098450*	74.34	669	7.53	2010	11,294	nucleus
*SlPLA1-48*	*Solyc09g098460*	51.41	457	8.58	1374	1374	chloroplast
*SlPLA1-49*	*Solyc10g038170*	50.13	443	9.03	1331	1332	cell wall, chloroplast
*SlpPLA19*	*Solyc10g078530*	45.56	435	8.08	1308	2303	vacuole
*SlpPLA20*	*Solyc10g079410*	93.7	826	7.88	2481	3511	vacuole
*SlpPLA21*	*Solyc10g079770*	96.8	861	6.45	2586	4647	vacuole
*SlpPLA22*	*Solyc10g080690*	46.7	432	8.98	1299	2245	vacuole
*SlPLA1-50*	*Solyc11g011120*	51.69	458	6.5	1377	7590	chloroplast
*SlPLA1-51*	*Solyc11g065530*	58.37	504	8.39	1515	1515	chloroplast
*SlPLA1-52*	*Solyc12g010910*	46.58	410	5.67	1233	1772	chloroplast
*SlPLA1-53*	*Solyc12g036490*	29.49	259	6.33	780	1607	chloroplasts, cytoplasm
*SlPLA1-54*	*Solyc12g055730*	58.31	503	5.93	1512	4002	chloroplast
*SlPLA1-55*	*Solyc12g088800*	56.26	480	8.77	481	5064	chloroplasts, cytoplasm

**Table 2:** A list of the conserved motifs found in SlPLA proteins.

Motif	Width	Sequence
1	41	GKNNWEGLLDPLDVDLRREJIRYGEFAQATYDAFNTDKASK
2	21	EDYSITVTGHSLGGALATLLA
3	24	RLGRRDIVIAWRGTVTTLEWVNBL
4	50	FKLEVNRDIALVNKQWDILKDEYCVPGSWWVEKNKGMVQQEDGSWILMDR
5	21	PVTVFTFGSPRVGBKNFKEAL
6	16	SNWIGYVAVATDEGKV
7	21	ELKNLKILRIVNSLDIVPKLP
8	21	SKYRVTKYLYATSSIPLPDAF
9	21	IGYFDVGQELMIDTTKSPYLK
10	23	PLVHHGFYSJYTSESSRSQFNKT

## Data Availability

The original contributions presented in this study are included in the article/Supplementary Material. Further inquiries can be directed to the corresponding authors.

## Supplementary Materials

The following supporting information can be downloaded at https://www.mdpi.com/article/10.3390/genes16020130/s1. Table S1. The primers used in real-time PCR (qRT-PCR) analysis in this study. Table S2. The SlPLA members identified in tomato genome.
