# Supplementary material for: The PLA Gene Family in Tomato: Identification, Phylogeny, and Functional Characterization
Source: Genes (Basel). 2025 Jan 23;16(2):130. doi: 10.3390/genes16020130 (PMC11855271; doi:10.3390/genes16020130)
Supplement: Supplementary file 1 [file genes-16-00130-s001.zip › Table S1.pdf]

Table S1. The primers used in real-time PCR (qRT-PCR) analysis in this study

gene ID	gene rename	primer	primer sequence(5'-3')
Solyc02g064700		Actin-F	TTGGGAAGGTTCTGGGGACT
		Actin-R	ATGGTTTCCTGCTGTGTCGT
Solyc01g008780	SlPLA2-1	SlPLA2-1-F	GCATACAGGTCCACGAGCTT
		SlPLA2-1-R	CCCAAATTGGTGACCCTCCA
Solyc01g067380	SlPLA1-1	SlPLA1-1-F	GCTTTCTGTGGCCTGGATCT
		SlPLA1-1-R	AGTAAGGGGGCAAATGAGGC
Solyc01g079600	SlPLA1-2	SlPLA1-2-F	TGTCTGAATAACCAGCAGAAGT
		SlPLA1-2-R	CAGACTGAGCGGCTTGAAGT
Solyc01g090220	SlPLA1-3	SlPLA1-3-F	CCTCGTACTGCCGATGACTC
		SlPLA1-3-R	TGAGGAAAGTTCACTGGCCC
Solyc01g094570	SlPLA1-4	SlPLA1-4-F	AAGGATCAGGTGCTTTGCGA
		SlPLA1-4-R	TGCTACAGTGGTCCGAGGTA
Solyc01g095720	SlPLA1-5	SlPLA1-5-F	CTGGACGGAGCAAGACAACT
		SlPLA1-5-R	CAAGACAGGTGGTTGTCGGA
Solyc01g104310	SlpPLA1	SlpPLA1-F	GGAAGGAAGGGATAGCGTCG
		SlpPLA1-R	CCAGGGCTAGTCACCGTTTT
Solyc02g014470	SlPLA1-6	SlPLA1-6-F	CAGTTGGAACGCTAAACGCC
		SlPLA1-6-R	TGAATCTCCCACTCGTGGAC
Solyc02g032850	SlPLA1-7	SlPLA1-7-F	GCATCGGTGGGTTGCAATTT
		SlPLA1-7-R	GGTCCATCCACGCATAACGA
Solyc02g065090	SlpPLA2	SlpPLA2-F	TTTCCTGAGAGCAGTCGCAC
		SlpPLA2-R	TGTGAAGACCACGGCTTGAA
Solyc02g065100	SlpPLA3	SlpPLA3-F	TAGTATGATCGGGGAGGCGG
		SlpPLA3-R	TCCCCTCGAACTGCTTCAAC
Solyc02g067660	SlPLA1-8	SlPLA1-8-F	GGTTTTGGAACGCCTCAAGC
		SlPLA1-8-R	TGTTACTGGTCTGTCCACCA
Solyc02g069400	SlPLA1-9	SlPLA1-9-F	CACCAGCAGCCAACCTCTAA
		SlPLA1-9-R	TGCACACCATAGCTGGGAAG
Solyc02g076990	SlPLA1-10	SlPLA1-10-F	TGGAGGCTTGCTTCAACCTT
		SlPLA1-10-R	GCTGTGTCCCGTCACAGTTA
Solyc02g077000	SlPLA1-11	SlPLA1-11-F	CCACTTCCTGATGGTTTCATCG
		SlPLA1-11-R	TTCTGCATAGTTCCTCTCCAAGC
Solyc02g077010	SlPLA1-12	SlPLA1-12-F	TTGTCGTTGCTTGGAGAGGAA
		SlPLA1-12-R	TAAATTGTGAACGGGCGCTT
Solyc02g077020	SlPLA1-13	SlPLA1-13-F	CCAAGGGGAGAACAAAACAAAG
		SlPLA1-13-R	TTGCTTGAGCCAATTCTCCG
Solyc02g077030	SlPLA1-14	SlPLA1-14-F	ACCCCTTGGCTGTTGATCTT
		SlPLA1-14-R	GCATATTTGGATGCTCTCTCCA
Solyc02g077100	SlPLA1-15	SlPLA1-15-F	AAGCGGCCACAGTATTGGTT
		SlPLA1-15-R	ATTGGCCTCTCCAGTTCGTG
Solyc02g077110	SlPLA1-16	SlPLA1-16-F	CGTCGGTACCTCATTCGCTA

		SlPLA1-16-R	CTCAATCCCCGTCCGAGAAA
Solyc02g077140	SlPLA1-17	SlPLA1-17-F	GGACAACCAGTGCCAGAGAT
		SlPLA1-17-R	ACCTTCTACGCATAGTGTGCC
Solyc02g077150	SlPLA1-18	SlPLA1-18-F	AAGGATTTCCAGTCACGGCA
		SlPLA1-18-R	CTCCACAACCCACCACGAAA
Solyc02g077160	SlPLA1-19	SlPLA1-19-F	TAGCCCACGAGTTGGAGAGA
		SlPLA1-19-R	ACGATCTGGCAGCTTCGTAA
Solyc02g077420	SlPLA1-20	SlPLA1-20-F	TTCGACGTTGGGCAGGAAAT
		SlPLA1-20-R	CGCGATATCACGATCCACCT
Solyc02g077430	SlPLA1-21	SlPLA1-21-F	GCACGGAATTGATGGTACGC
		SlPLA1-21-R	TCCCATTGTGCTTCTCGACC
Solyc02g080340	SlpPLA4	SlpPLA4-F	GAGTTGTGCACTTGGCACTG
		SlpPLA4-R	TCCATGCTCCAACCATCCAC
Solyc02g090490	SlpPLA5	SlpPLA5-F	AATGGTGCCACCCCTTTGAT
		SlpPLA5-R	ATAGATGCAGCCTCCCCTGT
Solyc02g090630	SlpPLA6	SlpPLA6-F	AGGTCTCATCAAGGTGGGAGA
		SlpPLA6-R	AGCCTGCCGGAATTTTCTCT
Solyc02g090640	SlpPLA7	SlpPLA7-F	TTGGTGGACCATTTGGTGGG
		SlpPLA7-R	AGTTGGGATTACGACGGCAG
Solyc02g090660	SlpPLA8	SlpPLA8-F	AGCAGGGAGTAGTACCGGAG
		SlpPLA8-R	TGCAATCTTCGGTTCCCCAA
Solyc02g090920	SlPLA1-22	SlPLA1-22-F	GGAACGTGTGAGTAGCAACAA
		SlPLA1-22-R	GCACGGAGACCGAGAGAAAG
Solyc02g090930	SlPLA1-23	SlPLA1-23-F	AAAGACTTCATCGACTGCCCT
		SlPLA1-23-R	GAAGACGGAAGAATCCGCCA
Solyc02g090940	SlPLA1-24	SlPLA1-24-F	CTTATCTGCAGAATGGCGAGC
		SlPLA1-24-R	ACAAAGGCTCCTTGGTCATGT
Solyc03g025510	SlPLA1-25	SlPLA1-25-F	GATTTCTTGCCACGGACAGC
		SlPLA1-25-R	CTGGGGCGTAAAGTCTTCGT
Solyc03g044710	SlpPLA9	SlpPLA9-F	CCTCTGCAGCCCCAACTTAT
		SlpPLA9-R	TGTGTGATTGCCATTAGAGTAGGA
Solyc03g083370	SlPLA1-26	SlPLA1-26-F	TAATGCCCTAGCTCCACCCT
		SlPLA1-26-R	TTGTGCCACGAAAGGCAATC
Solyc03g122280	SlPLA1-27	SlPLA1-27-F	CGTCGATCCACGGCTATTCA
		SlPLA1-27-R	TCGTCCTTCTGGTCTCGGAT
Solyc03g123750	SlPLA1-28	SlPLA1-28-F	TTGGACCTCATGCGCTTTCT
		SlPLA1-28-R	GATCTCCGGCAGTGGTATCG
Solyc04g078800	SlPLA1-29	SlPLA1-29-F	CGGACAACTTGATTGGAGGG
		SlPLA1-29-R	TGTAGTTTACCATTCCAAAAGCTG
Solyc04g079210/Solyc04g079230	SlpPLA10/11	SlpPLA10/11-F	GAACATCTGCTGCTCCGACA
		SlpPLA10/11-R	AGGTCGGATTGTTAGCAGCG
Solyc04g079240	SlpPLA12	SlpPLA12-F	TTGTTGATGGTGGAGTCGCA
		SlpPLA12-R	GCAGATCCTGTGCCTACAGA

Solyc04g079250	SlpPLA13	SlpPLA13-F	ATGGTGGTGTTGCGGCTAA
		SlpPLA13-R	CCCCCATTTTGCTGCCATAGA
Solyc04g079260	SlpPLA14	SlpPLA14-F	GGTGGCGTTGCGGCTAATAA
		SlpPLA14-R	CCCCATTTTGCTCCCATCAA
Solyc05g051280	SlPLA1-30	SlPLA1-30-F	TTATGCACCTGGCCGAATGT
		SlPLA1-30-R	TCTTCCGTCAACGGGGATTG
Solyc05g053910	SlPLA1-31	SlPLA1-31-F	TGGAGTTGGTACTCCCTATTTCAA
		SlPLA1-31-R	GGATCAAGCAACCCTTCCCA
Solyc05g053920	SlPLA1-32	SlPLA1-32-F	TCAAGATTACGTGCTCGTGGAA
		SlPLA1-32-R	ATCAAGCGGATCAAGCAAGC
Solyc05g056030	SlpPLA15	SlpPLA15-F	ATGGTTTTCTCCGGCGAGTT
		SlpPLA15-R	CGGTGCAGTACTGGAAAGGT
Solyc06g054550	SlPLA1-33	SlPLA1-33-F	GGACAACCTCGAATCGGGAA
		SlPLA1-33-R	GGTTATGGAGCCACACCTCC
Solyc06g060870	SlPLA1-34	SlPLA1-34-F	CAGCAGATGAGGTTCTGCCA
		SlPLA1-34-R	TCCTGTCATCACACACTGCC
Solyc06g071280	SlPLA1-35	SlPLA1-35-F	TCCGAGTCTTGTTTCTGGGC
		SlPLA1-35-R	CCAGCCATTTGCAGCTGTTT
Solyc06g083920	SlPLA1-36	SlPLA1-36-F	GCACTCTGAGTCTACAGTTCCA
		SlPLA1-36-R	TCCGGGGACAAACAACTCAG
Solyc07g014730	SlPLA2-2	SlPLA2-2-F	CCTGGGGAACAACCATGTGA
		SlPLA2-2-R	ATTTTTCCAGCAGCCACAGC
Solyc07g032220	SlPLA2-3	SlPLA2-3-F	GTAGGGTGGAGTGGTTGTCC
		SlPLA2-3-R	CCCGGACTTCTGCACTTTCT
Solyc07g055160	SlPLA1-37	SlPLA1-37-F	AGCAACTTACGACGCCTTCA
		SlPLA1-37-R	GCTTTATGGGCACCAACACG
Solyc07g056250	SlPLA1-38	SlPLA1-38-F	ATTGCTGGTCACTCCCTTGG
		SlPLA1-38-R	TCCGCCTTTGAAGGAAGCAT
Solyc08g006850	SlpPLA16	SlpPLA16-F	GCGGAAGAGGATCCAGCATT
		SlpPLA16-R	ATGCATCTAACATTGGCGGGA
Solyc08g006860	SlpPLA17	SlpPLA17-F	ACTGGTGCAGCAAGTTCGTA
		SlpPLA17-R	TCCCATGCAGTAGTTGTGCC
Solyc08g007225	SlPLA1-39	SlPLA1-39-F	TACACTTCCGGCGACCAATC
		SlPLA1-39-R	CTCGATTCGCGGGGATGAAT
Solyc08g022240	SlPLA1-40	SlPLA1-40-F	CAATCCACCTCCCCCTTGTC
		SlPLA1-40-R	CATGGTAGGTGTGTTGGCCT
Solyc08g023410	SlPLA1-41	SlPLA1-41-F	GGGGGTGCACTTGCTCTAAT
		SlPLA1-41-R	ACTCTTGGGGCTGCAAATGA
Solyc08g078090	SlPLA1-42	SlPLA1-42-F	AAACAGGCGGGGCAAAAATC
		SlPLA1-42-R	AAAGCCAATGCAGCACCAAG
Solyc08g082450	SlPLA1-43	SlPLA1-43-F	TCGACCAATGTGGACCTGTG
		SlPLA1-43-R	CCACCATGGAGGAGCAAGAG
Solyc09g056350	SlPLA1-44	SlPLA1-44-F	CCTTGAGGGCTCTGATGATGA
		SlPLA1-44-R	TCATCCCTGAAATGTGTTGCC

Solyc09g065240	SlpPLA18	SlpPLA18-F	TCTCCGTCGGAAGAAGAGGT
		SlpPLA18-R	GGCTTACACGTGTCCCTCAA
Solyc09g065890	SlPLA1-45	SlPLA1-45-F	TGACGAAACGACGAAACGGT
		SlPLA1-45-R	CCAAACGAGTCACCGTACCT
Solyc09g091050	SlPLA1-46	SlPLA1-46-F	GCGGGGACTTCAAGGAAGAA
		SlPLA1-46-R	AGAGTAGCCAATGCACCACC
Solyc09g098450	SlPLA1-47	SlPLA1-47-F	AAGCTTTGTGCTGTCTGGGA
		SlPLA1-47-R	CAGATGCCCGGTAAGTGACA
Solyc09g098460	SlPLA1-48	SlPLA1-48-F	AGCTCATTGACTCCTGCTCG
		SlPLA1-48-R	CCAACTCTTGGCCCTCCAAA
Solyc10g038170	SlPLA1-49	SlPLA1-49-F	TACGAGGCACAGCAACTTGT
		SlPLA1-49-R	TTGTAGACTCTGTTGCGCGT
Solyc10g078530	SlpPLA19	SlpPLA19-F	ATGGAGATTCCGATTCCGGC
		SlpPLA19-R	ACCATATCGGCGGTTCCATC
Solyc10g079410	SlpPLA20	SlpPLA20-F	CACAACGATCGAGCAGAGGA
		SlpPLA20-R	TGTTGTGACTCGTGGACTCG
Solyc10g079770	SlpPLA21	SlpPLA21-F	CCGAGCCCAAGGGTATCAAC
		SlpPLA21-R	AGCAACTGTATCCACGTTGAAG
Solyc10g080690	SlpPLA22	SlpPLA22-F	TGCATGCAGAGAGAATCGCA
		SlpPLA22-R	TAGTAGACCCACCTCCGTCG
Solyc11g011120	SlPLA1-50	SlPLA1-50-F	TCAGGGCGAGGAGTAACAGA
		SlPLA1-50-R	GCGCGAAAAGCTTCTCAACT
Solyc11g065530	SlPLA1-51	SlPLA1-51-F	TTTCGCTCTGCCACGAGTAG
		SlPLA1-51-R	CCATCGCTCGTTCTCACCAT
Solyc12g010910	SlPLA1-52	SlPLA1-52-F	CCCGAAGTCGTCTTTCACGA
		SlPLA1-52-R	GCACCAAGACTATGCCCTGT
Solyc12g036490	SlPLA1-53	SlPLA1-53-F	GGGAAGCATGGGGTAAGGAA
		SlPLA1-53-R	CAACGGTTGGGCTAAACCTC
Solyc12g055730	SlPLA1-54	SlPLA1-54-F	GGAGGAGCGTTGGCTATACT
		SlPLA1-54-R	AGGCACCGGATGTTCCAAAT
Solyc12g088800	SlPLA1-55	SlPLA1-55-F	GGCATCGACGATTCGAGGTT
		SlPLA1-55-R	TCCGGCCTTACCACTTTTCC
Solyc12g098730	SlPLA1-56	SlPLA1-56-F	CCTCGAAGGAGGGTGAAAGG
		SlPLA1-56-R	CCAATGCACTCCCCATGCTA
Solyc03g026280	CBF1	qRT-PCR-CBF1-F	GTCATCGTCGTTTTCTGAAG
		qRT-PCR-CBF1-R	AACGGCCTCTTAATGCTAAA
Solyc03g124110	CBF2	qRT-PCR-CBF2-F	TTCGATCGGAAGAAGTTTCA
		qRT-PCR-CBF2-R	CAAGTAATCCTGGCATGGAA
Solyc03g026270	CBF3	qRT-PCR-CBF3-F	CGCCGAAATCTTCCGACCT
		qRT-PCR-CBF3-R	CGGCATGCAGAATAACGCTT
Solyc04g082200	COR47-like	qRT-PCR-COR47-like-F	GGCATCCGTTGAAGAGACTG
		qRT-PCR-COR47-like-R	TCCTCCTTGGGTTCCACTTC
	LOXD	qRT-PCR-LOXD-F	CCTCAAGGAGGAGCACATTC
		qRT-PCR-LOXD-R	AATGGGCTTAAGTGTGCCAA

AOC	qRT-PCR-AOC-F	CAAGCTATCTTCTGCCTTCCA
	qRT-PCR-AOC-R	AGTTCTTGAACTTCAGTGTTAGT
ACX	qRT-PCR-ACX-F	TTGCAAGGGTATGGGTTGAC
	qRT-PCR-ACX-R	GGTCCTCTTAGCCAAAGCTC
KAT	qRT-PCR-KAT-F	TGAAGGGACTCGTTCAGTAGT
	qRT-PCR-KAT-R	TATGCAGAACTGTCACCAGC

All primers were designed within the exon of the gene sequence.
