# Supplementary material for: The PLA Gene Family in Tomato: Identification, Phylogeny, and Functional Characterization
Source: Genes (Basel). 2025 Jan 23;16(2):130. doi: 10.3390/genes16020130 (PMC11855271; doi:10.3390/genes16020130)
Supplement: Supplementary file 1 [file genes-16-00130-s001.zip › Table S2.pdf]

Table S2. The SlPLA members identified in tomato genome

Solyc01g008780 SlPLA2-1

MDFSFFGNFPWFKPHSANDMASTVASTSTLMQTPKQNAQFWKWTVFSFLPWAKVAEGNIQMPTTVNKKLKRRPSREGVDSLARKSAIRFRPYVSKVPWHTGPRAFLSQLFPRYGHYCGPNWSSGKDGGSPIWDRRPIDWLDFCCYCHDMGYDSHDQAELLKADLAFLECLEKPNMSTRGDPHVALLYKTMCTSGLRNILIPYRQQLITLQSKQLCFGFGWLGGIMEPAKCLKDRFVWLQK*

Solyc01g067380 SlPLA1-1

MPSGAYVGVAQDLNAIVIAFRGTQESSLQNWIEDLYWKQLDISYPGMEDAMVHHGFYSAYHNTSLRPGVLTAVKSAKEFYGDIPIMVTGHSMGGAMAAFCGLDLTVHLGCQNVSVMTFGQPRIGNAAFVSYYRERVPNTIRVTNRHDIVPHLPPYYQYFPHKTYRHFPREVWLYDLGFGSLVYTVEKVCDNSGEDPSCSRSVKGNSVKDHVRYFGVKLSCDVSAGCRIVMGNGLASYHTTDNDGNIIFSRNISSVLRMNVESSEEGKSL*

SlPLA1-1 supplementation

MQVGDPHIWTFHLGLSFGLKIIRHWLPVFPLFFYSTWARLMQVIFLAELFVLDIVIQIMDGKNWLKVVIFLCLIAVSTGREFKVKAKNKHHAAIYNHTLATILVEYASAVYVSDLTELFAWTCSRCNGLTKGFQILELIVDVQRCLQAYVGVAQDLNAIVIAFRGTQESSYPSHIHYEDDERRYILHPKVAIIGVTILSGTEIPIKMGNAPSLSASLQNWIEDLYWKQLDISYPGMEDAMVHHGFYSAYHNTSLRPGVLTAVKSAKEFYGDIPIMVTGHSMGGAMAAFCGLDLTVHLGCQNVSVMTFGQPRIGNAAFVSYYRERVPNTIRVTNRHDIVPHLPPYYQYFPHKTYRHFPREASSSTQNPVVLDFELHKLVTHSSDSASCCLDSWLHYVDCEILKLQVWLYDLGFGSLVYTVEKVCDNSGEDPSCSRSVKGNSVKDHVRYFGVKLSCDVSAGCRIVMGNGLASYHTTDNDGNIIFSRNISSVLRMNVESSEEGKSL

Solyc01g079600 SlPLA1-2

MDGLCLTGGGIQGMAGPIAIAGGLDVRSTQFSTSAVGRSSMSVEKTSSCRNKSWGFSFRYPLRSFWSGGKGRYDAIAVDDAVLMEEKNEEKNENWVLKILHIRSLQEEKEVGEEKDGGVEDLGEKVDGEGGDDIEDDSEENIKHDDGDEEECDVCCVDDDEKFKFDRKSFSKLLRRVTLAEARLYAQMSYLGSLAYGIPQIKPENLLRNHGLRLVTSSCEKREQALKVEKEKAEAEDQEKKENEIAQTQGEERSTTIPVEGNGKTSGNRISASTAYHIAASAASYLHSHTMSILPFKSSKTMPNKDSSETTVGCDDNIDAMNREVASFMVTSDSVTSVVAAKEEVKQAVADDLNSNHSSPCEWFVCDDDQSLTRFFVIQGSESLASWKANLLFEPVKFEGLDVMVHRGIYEAAKGMYAQMLPEVRSHLKSHGSRANFRFTGHSLGGSLSLLVNLMLFIRGEVPLSSLLPVITFGAPSIMCGGDRLLRRLGLPRSHVQAITMHRDIVPRAFSCNYPNHVAEFLKAINANFRNHQCLNNQQKLLFAPMGEFLILQPDEKFSPNHDLLPSGSGLYLLNCPVSDSTEAEKQLQAAQSVFLNSPHPLEILSDRSAYGSGGTVQRDHDMTSYLKSVRNVIRHELNNIRKAKRKQRRRVWWPLVSPSGVNAGIVVRRYVESGSMGHGQINFAGILQSGKESLKRFSTLVASQHMHLLVVLLFPARLLVVGTFSMFNFR*

Solyc01g090220 SlPLA1-3

MAEEEEIVGEVEALQAVYGDDCLLLQTYPPSFHLHIKPRTADDSSQQFVEAIIGIQAGSKYPDEPPAIRIIDSKGLDEQRQKQLISCISERASELSSCLMLVALCEEAVERLSSMNHPDGECPLCLYPLVAEDSGSSEPFMKLMSCFHCFHCECIIRWWNWLELLKESDAPTASGSASSSGSIRDQDEESRRKCPVCRKSFLAKDIEHVLDFVKTQHAVTSSRSEVNNEDKILSSVSEKLRRAKFDAILKLQQEKGGLIEIKKHEVLRPGIYLPQPAALPSTASTEEAKEQQDKDLAANSRTNSSGSTNKPNTSRARNSSTKKHQGHNSRKQVAQSSRKQVTQWVKKENSNIYIIMRCPNQLFLTNKYFGVVLPNTWQYYSASTLNLSTITHRIIKISNSCHLGSSHCLVFNDEDKLGKRWMEFQGINNWDGLLDPLDDDLRGEILRYGEFVEAAYGCFDFDTSSATYATCRYPKRSMLMQRGLGRSGYKVVKNLHATCAVQMPRWLEKFPNLKSPQSSWIGYVAVCDDMDEIARLGRRDIVIAYRGTVTYSEWLENLRVTLTCLPDDMSPNENNKPMVQSGLLSMYTTKIEGHMPSLQETIREEIISILNNYSDEYSLSITITGHSLGAALATLTAYDVTTKFNNLPMVTVLSFGGPRVGNKSFRYQLEKNGTKVLRIVNSDDPITKVPGIVIDDDDVAHRGDAMVARLPSWLDKYMEDAPWVYAEVGKELKLSSKDSIRKGSVAKCHDLKTYLYLVNNFVSSSTCPLRATVRNYSFRLYLRNTI*

SlPLA1-3 supplementation

MEFQGINNWDGLLDPLDDDLRGEILRYGEFVEAAYGCFDFDTSSATYATCRYPKRSMLMQRGLGRSGYK

VVKNLHATCAVQMPRWLEKFPNLKSPQSSWIGYVAVCDDMDEIARLGRRDIVIAYRGTVTYSEWLENLRVTLT
CLPDDMSPNENNKPMVQSGLLSMYTTKIEGHMPSLQETIREEIISILNNYSDEYSLSITITGHSLGAALATLTAYDV
TTKFNNLPMVTVLSFGGPRVGNKSFRYQLEKNGTKVLRIVNSDDPITKVPGIVIDDDDVAHRGDAMVARLPSW
LDKYMEDAPWVYAEVGKELKLSSKDSIRKGSVAKCHDLKTYLYLVNNFVSSSTCPLRATVRNYSFRLYLRNTI

Solyc01g094570 SlPLA1-4

MSVACCIPVVECVYCLGCIRWVWKKFLYTAGRESENWGLAIASEFEPVPRFCRYIMAVYEDDIRNPISTPP
GGYGIDPDWVIVKRSHEDTQGKVSPYLIYVDHQNADIVIAIRGLNMAKDTDFLVLLDDKLGQAEFDGGYVHNG
LLKAAEWVWEAESQLLRELVERYPDYTLTFAGHSLGAGVVTLLTMLTVKNREKLGFLDRKRIRCFAIAPTRCVSLN
LAVRYADIINSVVLQDDFLPRTTVALEHAFKSLLCFPCLMCIMCLKDTFTMEEKMLKDPRRLYAPGRLYHIIVRKPF
SSANIKPIVRTAIPVDGRFEHIVLSCNMTSDHGILRILTESQRTIDLMLERHQSTDSMNIPEQQRMERRDSLAKE
HMEEHKAALQRAVALDVPQAYSPSAYGTFRNIEQGPDFGQPGESSLPISQKRREIWDELAGRLFLDR*

SlPLA1-4 supplementation

MSVACCIPVVECVYCLGCIRWVWKKFLYTAGRESENWGLAIASEFEPVPRFCRYIMAVYEDDIRNPISTPP
GGYGIDPDWVIVKRSHEDTQGKVSPYLIYVDHQNADIVIAIRGLNMAKDTDFLVLLDDKLGQAEFDGGYVHNG
LLKAAEWVWEAESQLLRELVERYPDYTLTFAGHSLGAGVVTLLTMLTVKNREKLGFLDRKRIRCFAIAPTRCVSLN
LAVRYADIINSVVLQDTFTMEEKMLKDPRRLYAPGRLYHIIVRKPFSSANIKPIVRTAIPVDGRFEHIVLSCNMTSD
HGILRILTESQRTIDLMLERHQSTDSMNIPEQQRMERRDSLAKEHMEEHKAALQRAVALDVPQAYSPSAYGTFR
NIEQGPDFGQPGESSLPISQKRREIWDEVSWNIFNAPVQVSRMDFSKEQVVAVDILSWNTVAG

Solyc01g095720 SlPLA1-5

MACSCISIMNSPINRTSKDAWTEQDNFRRSFPSKETREKAQMRRSYSDNHLSCRANRIQSLETQPKLKSS
RSTGGPFKIQLSSSFLPDSLRSFLFDIETSKDINIDGVIFESDHDHDDDNEGIETEEETRRSNWIQRLVELKRNWIE
KQKEEDAEISEDNLENSGEDCEEEGCEVDYEGDNEEADEMNIDRESFSRLLRRVSWSDSKLFSKLAFLCNMAYVI
PEIKARDLERCYGLDFVTSSLVKKAEAMAIKAKFDKDSVCVPVPSSDNSVPNRDKTEEIEHKCLPPPSVAYDIAAS
AASYVQSRAKGLLSVGSESKLVVDDATLKANKGCSADEKDNSSQRVYKSEMAAYVAASTMTTMVAADEKQKLE
AARDLQSLQSSPCEWFICDDLTTYTRCFVIQGSDSLASWKANLFFEPCKFEEMDVLVHRGIYEAAKGIYDQYMP
EIMEHLQRFGNKAKFQFTGHSLGGSLSLLVNLMLLTRKVVKPSSLLPVVTFGSPFVFCGGQKVLNDLGLDENHV
QSVMMHRDIVPRAFSCNYPNHVAQVLKRLNRTFRSHPCLNKNKLLYSPMGKIFIIQPDERSSAPHPLLPPGSGIY
SLDSTNCAFTRRAFRVFLNSPHPLEILSVPTAYGSGGTILRDHDSSNYLKAVNNIIRQHTKLLVRRVRKQRNLIWPL
LASQSPHAWSHERDIEDRGILRKEIMSSV*

Solyc01g104310 SlpPLA1

MFQKLTILQSKGKITSIYVWTKEIQIHMKHITHQKSSSSSSQPNKSMAASLSSLFLSNPRPFRPPINPKSNPH
LVFPLRTLNFSAKPNPPPPNSNAKQPPTIREPPLPTPPVGDEKKSFAVATGELFLGIASRVLRRGSSLVNGSNEEPA
GVTMFKDDEESESYFLKRRKEGIASVVEDPVQPEVVWEQTEKDVEAEKSLKTVTSPGFSFSAAGLLFPYHLGVAK
LLIEKGYIKETTPLAGSSAGAIVCVVVASGASMQEALDATKILAQDCRLKGTAFRLGAVLREVLEKFLPDDAHIRCN
GRVRVAVTQILWRPRGLLVDQFDSKEDLINAVFTSSFIPGYLAPRPATFFRNRLCIDGGLTLFMPPTSAAQTVRIC
AFPASRLGLQGIGISPDCNPDSRATPRQLLNWALEPAEDDILDKLFEQGYVDAAVWAKENPVEDLIRDDDSSSL
GISLVQ

SlpPLA1 supplementation

MAASLSSLFLSNPRPFRPPINPKSNPHLVFPLRTLNFSAKPNPPPPNSNAKQPPTIREPPLPTPPVGDEKKS
FAVATGELFLGIASRVLRRGSSLVNGSNEEPAGVTMFKDDEESESYFLKRRKEGIASVVEDPVQPEVVWEQTEKD
VEAEKSLKTVTSPGFSFSAAGLLFPYHLGVAKLLIEKGYIKETTPLAGSSAGAIVCVVVASGASMQEALDATKILAQ
DCRLKGTAFRLGAVLREVLEKFLPDDAHIRCNGRVRVAVTQILWRPRGLLVDQFDSKEDLINAVFTSSFIPGYLAP
RPATFFRNRLCIDGGLTLFMPPTSAAQTVRICAFPASRLGLQGIGISPDCNPDSRATPRQLLNWALEPAEDDILDK
LFEQGYVDAAVWAKENPVEDLIRDDDSSSLGISLVQ

Solyc02g014470 SlPLA1-6

LCISLISKGSMRNWFKRKMKKNTEKKDSSIAKRWMLLSGKNNWEGLMDPLDYNLRRYIIHYGEMAQAS YDNFNSNKASKNAGNNRYSRNNFFTKVGLDKDHNNPFKYRVTKYLYATSSIQVPEAFIVKSLSRESWSKESNWI GFIAVCTDEGKIALGRREILISWRGTVQTLDWVNDLDFFQVSAPEIFRGNTEPQIHRGWYSIYTSDDPRSPFNNT SVRDQVVEEVKRLVEEYKSEKMSITITGHSMGAAVGTLNAIDIVVNGFNKGCLVTAILFASPRVGDSNFVNAFSK LENLRILRVTNCLDIIPNYPLIDYSEIGVELGIDTTKSKYLKVPGDIRSWHSLEAYMHGVAGYQGANGGFKLEVGR DISLLNKHLNALKDEYCVPTCWWVEKNNGMVQQDDGYWKLMDHEDDDDSVYA*

SlPLA1-6 supplementation

MKKNTEKKDSSIAKRWMLLSGKNNWEGLMDPLDYNLRRYIIHYGEMAQASYDNFNSNKASKNAGNNR YSRNNFFTKVGLDKDHNNPFKYRVTKYLYATSSIQVPEAFIVKSLSRESWSKESNWIGFIAVCTDEGKIALGRREILI SWRGTVQTLDWVNDLDFFQVSAPEIFRGNTEPQIHRGWYSIYTSDDPRSPFNNTSVRDQVVEEVKRLVEEYKS EKMSITITGHSMGAAVGTLNAIDIVVNGFNKGCLVTAILFASPRVGDSNFVNAFSKLENLRILRVTNCLDIIPNYPL IDYSEIGVELGIDTTKSKYLKVPGDIRSWHSLEAYMHGVAGYQGANGGFKLEVGRDISLLNKHLNALKDEYCVPT CWWVEKNNGMVQQDDGYWKLMDHEDDDDSVYA

Solyc02g032850 SlPLA1-7

MVSEASSFESSETLAALVASTPLLEESWKVCGVADASVGCNFAVNRVGETAYVGFSGVKLGAGVDQSCRN LVPLPDELFFSLCVDGPDPAMVHAGLLHLFQSVYIDNLFRDQMVEIMNTSKSIVITGHSIGGAIASLLTLWLLCRL QTICSVICITFGSPMLGNQSFSRAILQKRWAGHFCHVVSQHDIVPRLFFAPSCCFQFISYENKTQLFHVVLDSLGV VSRGECKSSFCPSGSYLFCTNKGAVCVDNGMVVIKLLYFTLLNSSQSSSLEDHLDYADFIQKVQWQFIENRSFTE GSIPKSSYKAGITLALESLGIASHEVNFEDAKEALKKAKKLGRTRNLNSANLAIGLSKINPFRAQIEWFKASCDNSA EQMGYYDSFKQRGASKRGFKVNMNRIKLAQFWDSLIDKLEANELPYDFHKRAKWVNASQFYKLVVEPLDIAEY YRTGMHLVKGHYMQHGRERRYKIFDKWWKTENDTDNPTARSRFASSTQDSCFWARVEEARDSLIKVRAEGD ARKFLKMLEDVTKFDQYAKRLIENKEISQDVLAKNSSYTKFIEEWKDLQSQLQLLQPQFP*

Solyc02g065090 SlpPLA2

KPILSYSFSSSVIKLKNIMGRIFVAALTLLVTLQVLHIPIAFAATTGKKITILSIDGGGIRGIIPGTILAFLESKLQEL DGPNARIADYFDVVAGTSTGGLIATMLTAPNKDNRPLYAAKNITNFYMDHGPKIFPESSRTSFLKRLANIFGGPK YDGKYLRTLVRSILGNLTVTQTLTQLVIPSFDIKRLQAVVFTTSDAKAHVSKNALLSDVCLSTSAAPTYFPVHYFETR DSQGKTRTFDLVDGGVAANNPTLVAITQTSKEMMLGKLQNAGLKPMDCKKMLVLSLGTGIAKDEKKYSAAAA STWGVLGWLYNNGASPLLDVYGDASSDIVDVHLSTIFQSLDSQNNYLRIQDDSLSGEAASMDVATKKNMEALV QIGNDRLKKGVSRVNLDTGRYEEVSGEGTNEEALIRFAKLLSEQRKIRQPVDEFISIRKIL*

SlpPLA2 supplementation

MGRIFVAALTLLVTLQVLHIPIAFAATTGKKITILSIDGGGIRGIIPGTILAFLESKLQELDGPNARIADYFDVVA GTSTGGLIATMLTAPNKDNRPLYAAKNITNFYMDHGPKIFPESSRTSFLKRLANIFGGPKYDGKYLRTLVRSILGNL TVTQTLTQLVIPSFDIKRLQAVAKAHVSKNALLSDVCLSTSAAPTYFPVHYFETRDSQGKTRTFDLVDGGVAANN PTLVAITQTSKEMMLGKLQNAGLKPMDCKKMLVLSLGTGIAKDEKKYSAAAASTWGVLGWLYNNGASPLLDV YGDASSDIVDVHLSTIFQSLDSQNNYLRIQDDSLSGEAASMDVATKKNMEALVQIGNDRLKKGVSRVNLDTGRY EEVSGEGTNEEALIRFAKLLSEQRKIRQPVDEFISIRKIL

Solyc02g065100 SlpPLA3

MARIFVVALLITFLVLLQQYPIACVATKRKTVTILSIDGGGIRGIIPSTILAFLESKLQELDGANARIADYFDVIA GTSTGGLVTTMLTAPNKDNRPLYAAKDINNFYMEHAPKIFPQRSRNNFMKKMFNLFGGPKYDGEYLRLLVRSE LGNLTMKQTLTDTLIPTFDIKRLQPIIFTTTDARAIVSKNARLSDVSLGTSAAPTYFPVHYFETKDAQGKIRTFDLVD GAVAANNPTLLAITHISREMMTRRLKYEDAKTVDCKKMLVLSLGTGTGKNKEKYNAATASKWGLLSWMYNHG AIPLLDIFTDAITDIVDIHVSTMFQSLHNHKNYLRIQNDSMIGEAASMDISTIENMQTSVQTGKDLLKKPVSRVN LETGRVEAVRGEGTNEEALTRFAKLLSEERKFRRLET*

Solyc02g067660 SlPLA1-8

MSQVSLFSSGQELANLFLSSDLLHHSWITISALSSHPYINKPILFKVYPYHSNGAIVAFVSSPTCNLQKEMVS SEELQGSQSPFDFISTKLNPHFSVNKAAITLFASLLNDLSALKEQLDSFSPLIITGVSLGGSVASLFTLWLLKDSNKR PTCITFGSPLLGDSGLQQAISERPSWNSSFLHVVSNQDPIPRSLISPTNVFAGSIPQPCIYMPFGMFLLCSDSDCS CFEEPESVLDLMTEMNLNSQHQDNHSLAFDYEQVLERLKHRVILKGASQLFQFSVDQLQAGIDLQLEAIGIGGQ TSNMNSIRTKVKKRVEESFAKKRNAFDPGKKLNKMKEAMTWLEWYKKVTLKEGGYYDSYKRSEYRGRDAVKS RQEIVKHQRVLTKYWKTMVAEAEKMPQREEAVFRTRWLYAGTNYRRMVEPLDIAAYYMKPGNTDYVNLGRS EHYKKLEEWRREDNPSGSGNDRRKCVSLTEDSCFWAYVEEAIINSKRLREGSLQEKENAREYLVNFGDML*

SlPLA1-8 supplementation

MAQKVHVVESSSGLLQEQHNHVQDQEEDANYVSSYQGGPNKQRTNYQGGQDHDQCGSVVSGYLDSL RYPSLCRILEIGKVLWDRCYAFRNISNSPKAKMSQVSLFSSGQELANLFLSSDLLHHSWITISALSSHPYINKPILFK VYPYHSNGAIVAFVSSPTCNLQKEMVSSEELQGSQSPFDFISTKLNPHFSVNKAAITLFASLLNDLSALKEQLDSFS PLIITGVSLGGSVASLFTLWLLKDSNKRPTCITFGSPLLGDSGLQQAISERPSWNSSFLHVVSNQDPIPRSLISPTN VFAGSIPQPCIYMPFGMFLLCSDSDCSCFEEPESVLDLMTEMNLNSQHQDNHSLAFDYEQVLERLKHRVILKGA SQLFQFSVDQLQAGIDLQLEAIGIGGQQTSNMNSIRTKVKKRVEESFAKKRNAFDPGKKLNKMKEAMTWLEW YKKVTLKEGGYYDSYKRSEYRGRDAVKSRQEIVKHQRVLTKYWKTMVAEAEKMPQREEAVFRTRWLYAGTNYR RMVEPLDIAAYYMKPGNTDYVNLGRSEHYKKLEEWRREDNPSGSGNDRRKSKIRQTMNESSTSLRITRGSPLH VNVVDILPSFSMGLTQEFGVNVGSLGKSKQLIQEQTMEELRSKEKKRPYGSSTSY

Solyc02g069400 SlPLA1-9

MVSLYNTGLELANLLLGSDLLNQSWEAISKLQKQNLTLLEDPSLPFSVKNQIYESPSKGSIIAFVSSPNCAVN HLKEEFRELISSDQITSFDFLRTRSNTSSISIHKAAFALFASLEKELSLLKQQFTSSQPLIVTGHSLGGSVASLFTLWLL ESLPSYGVQHILCITFGSPLLGNNGFQKAMSEHPMWSSCFLHVVSDKDPVPGFLISDHNASAATLTTPTWYMP FGTYLFGSESGFSCFEEPQSILELLMIMSSRCAESENLNNCSQVIDYGHILEQLKNKAICRGISELPDTISNPLQAGT YLLLEATGVGMVQENVNLSYLAMNIAKRAEVQAKHKQKLFDPSKISKTKKSMSYLEWYMKNSLEEAGYYDKYK TRRSRSRDEIASTENLIKHHRTLKLFWKRTVTEVENLPWKEGRTRRKRLLYAATNYRRMVEPLDIAEYYGRGKRN YVLHGRSEHYKLLEKWLTGENTQTYQRSQASSLTVDSCFWAYVEEAMISCQVLKDGQSSSPQDQESARENLIKF EQYVMDMVNNFLVSPEIFLSQSSFMLWWNEYSKVVGNSYTSPLTNFMNDGFRRYA*

Solyc02g076990 SlPLA1-10

MGSMADKWEELSGKNNWEGLLNPLDLDLRKYIIQYGELAQAINDTFITEKASKYAGASRYSMENLFTKV GLDPTKYRVTKYFYATASIPLPDGVFFVKSLSREAWSKESNFMGYIAVATDEGKVSLGRRDIVINWRGSMQTLE WVNDLQFVLVPAPEVFGDGGLLQPLVHQGFYNVYTASSSRSQFNVTSARDQVMEEVKRLVEEYKNEEVSITVT GHSLGASLATLNAVDIAFNKINKASNGKEFPVTAFPFACPKVGDLQFKAAFDKIIGLRILRIDNLLDIAPKYPPIGYF DVGQELMIDTTKSPYVKPPGQPVNWHSLESYLHGIAGTQGTGLLAGFNLEVNRDISLINKQLDGLKDEYCIPVN WWVEKNKGMVQQEDGSWLLLDRDDYDF*

Solyc02g077000 SlPLA1-11

MAEKWEELSGKNNWEGLLHPLDVDLRKYIIHYGELAQATYDTFITERASKYAGASRYSMENLFTKVGLDP NKYRVTKFFYATASIPLPDGFIVKSLSREAWSKESNFMGYIAVATDEGKVSLGRRDIVIAWRGTMQKLEWVNDL QFLLVPAPQVFGDGGLLPLFQPLVHHGFYNAYTSSSSRSQFNLTSARDQVIEEVKRLVEEYKHEEVSITVTGHSLG ASLATLNAVDIAFNGINKTSEGKEFPVTAFPFASPKVGDLQFKAAFDKIKGLRVLKIHNLLDIVPKYPPIGYFDVGQ ELMIDTTKSPYVKPPGEPVSWHLLEPYLHGVAGTQGLGLLAGFKLEVNRDISLVNKQWDVLKDEYCIPGLWWV EKNKGMVQQEDGSWLMLDRDEYDF*

Solyc02g077010 SlPLA1-12

MAGMAEKWEELSGKSNWDGLVHPLAVDLRKYIIQYGELAQATYDTFITERASKYAGASRYSNENFFTKVG LDPNKYGVTKFFYATASIPLPDAFITRSFSREAWSKESNFMGYVAVATDEGKVSLGRRDIVVAWRGTKQALEWV

NDLQFLLVPAPNVFGNGGLLPLFQPLVHHGFYNIYTSESARSQFNQTSVRDQVMEEVKRLVEEYKDEEVSITVTG
HSLGASLATLNAVDIAFNGINKTSEGKEFPVTAFVFASPKVGDINFLNKFSKLKHLHILRIHNLLDIVPKYPPIGYFD
VGQEIMIDTTKSPYVKPPGEIVSWHLLEPYLHGVAGTQGLGLLAGFKLEVNRDISLVNKEWDILKNEYCVPAFW
WTEKHKGMVQQENGSWLLMDRDEYEF*

SlPLA1-12 supplementation

MAGMAEKWEELSGKSNWDGLVHPLAVDLRKYIIQYGELAQATYDTFITERASKYAGASRYSNENFFTKVG
LDPNKYGVTKFFYATASIPLPDAFITRSFSREAWSKESNFMGYVAVATDEGKVSLGRRDIVVAWRGTKQALEWV
NDLQFLLVPAPNVFGNGGLLPLFQPLVHHGFYNIYTSESARSQFNQTSVRDQVMEEVKRLVEEYKDEEVSITVTG
HSLGASLATLNAVDIAFNGINKTSEGKEFPVTAFVFASPKVGDINFLNKFSKLKHLHILRIHNLLDIVPKYPPIGYFD
VGQEIMIDTTKSPYVKPPGEIVSWHLLEPYLHGVAGTQGLGLLAGFKLEVNRDISLVNKEWDILKNEYCVPAFW
WTEKHKGMVQQENGSWLLMDRDDNTLLVLALQVNNSPPHAVANVRLFFTRAVAEGQKARQYLVWTLVYQL
GSSDKIFVHLCQSLFLSVRSASFVV

Solyc02g077020 SlPLA1-13

PRGEQNKE*KKHKRSRKRIMACIFAEKWEELSGKNNWDGLLNPLDLDLRKYIIHYGELAQATYDTFISERA
SKYAGASRYSMENFFTKVGLDPKKYCVTKYFYATSSMPLPDAFITKSLSREAWSKESNFMGYIAVATDEGKASLG
RRDIVINWRGTLQVLEWVNDLQFLLVPAPQVFGDGGLLPLFHPLVHHGFHNIYTTENPRSQFNKTCVRDQVM
EEVKRLVEEYKDEEVSITVTGHSLGASLATLNAVDIAFNKINKASNGKEFPVTAFVFASPKVGDVNFLNAFSKLKH
LHILRIHNVLDIVPKYPPIGYFDVGQEIMIDTTKSPYVKPPGEPVSWHLLEPYLHGIAGTQGIGMLAGFKLEVNR
DISLVNKQWNVLKDEHCIPPLWWSEKHKGMVQQEDGTWLLQDRDEYEF*

SlPLA1-13 supplementation

MACIFAEKWEELSGKNNWDGLLNPLDLDLRKYIIHYGELAQATYDTFISERASKYAGASRYSMENFFTKVG
LDPKKYCVTKYFYATSSMPLPDAFITKSLSREAWSKESNFMGYIAVATDEGKASLGRRDIVINWRGTLQVLEWV
NDLQFLLVPAPQVFGDGGLLPLFHPLVHHGFHNIYTTENPRSQFNKTCVRDQVMEEVKRLVEEYKDEEVSITVT
GHSLGASLATLNAVDIAFNKINKASNGKEFPVTAFVFASPKVGDVNFLNAFSKLKHLHILRIHNVLDIVPKYPPIGY
FDVGQEIMIDTTKSPVKPPGEPVSWHLLEPYLHGIAGTQGIGMLAGFKLEVNRDISLVNKQWNVLKDEHCIPPL
WWSEKHKGMVQQEDGTWLLQDRDEYEF

Solyc02g077030 SlPLA1-14

MGSMAEKWEELGGKNNWNGLLNPLAVDLRKYIIHYGELAQATYDTFIMERASKYAGASRYSMENFFTK
VGLDPNKYRVTKFFYATSSIPLPDGFIVKSFSREAWSKESNFMGYIAVATDEGKASLGRRDIVVNWRGTIQKME
WVNDLQFLLIPAPKIFGAGGLLPLFKPLVHHGFYNVYTSASSRSQFNKTSVRDQVIKEVKRLVEEYKDEEVSITVT
GHSLGASLATMNAVDIAFNKINKASNGKEFPVTAFAFASPKVGDIQFKATFDKLKHLHILRIHNLLDIVPKYPPIGY
FDVGKELMIDTTKSPYVKPPGENVSWHLLEPYLHGVAGTQGLGLFAGFKLEVNRDISLVNKQWNILKDEYCIPG
MWWVEKNKGMVQQEDGSWLMLDRDEYDF*

Solyc02g077100 SlPLA1-15

MGCINKGLEAVSIKIRNFFKKKKGIENRDNSVAERWEILNGKNNWEGLLDPLDYDLRRYLIHYGQMPQAI
CDSFNSEIASKNLGTNRYSKKNFFKRVGLDKNNPFKYEVTKYIYGASDVPSKTEKIKSSNWIGFVAVATDEGKVAL
GRRDILIAWRGTMCPAEWNDDSDWVLVPPTKIFGENTHDTLVHRGFYSVYTSLNDASKFNRTASARDQVIEEV
KRLVEQYKRDKVSITLSGHSIGSSLSTLCAIDIVVNKINKEFPVTAFLFASPRTGEANFKKAHQHLKNLQILRITNAL
DEVPEKPDRGQVEGSDTDWRVYEHVGYEVKIDTTKSEYLKKDVNNHSLEVYLHGIAGTHGPEGEFKLEITRDIAL
VNKETDALKNEYGVPVYWRTVQNKGMVQQEDGSWILNDREDDTDLSE*

Solyc02g077110 SlPLA1-16

QLKSKIPQTTKMFKATKKETIEKMSMPKLLLALCIFSVVSPTIEMMQNVDKNMGNITERWRILSGNSNW
EGLLDPLDNDLRRYLIRYGEMTQAARDAFNTNKISKYAGTCRYSKKNFFSRTGIEISNPFKYEVTKYIYATSAVQVP
EALFIKSLSREAWSKESNWAGFVAVATDKGKIALGRRDIVIVWRGSVQTLEWFNDFDFIQVSATKIFGEKSDPKV

HHGWYSIYTSDDSRSLFNKESARDQVLGEVKRLMEQYKTEEVSITVTGHSMGASMATLNAGDIVFNGINKGFPVTAFLFASPRVGDENFKRTFSKLENLRALRIRNAPDLVPSYPLFGYSDVGVELAIDTRKSGYLKSPGDQSSFHNTDCYLHGIAGTQGSKGGFKLEVERDISLINKYLDALKDEYGVPTSWWVEKNNGMVQQQNGTWILVDHEDDDF*

SlPLA1-16 supplementation

MSMPKLLLALCIFSVVSPTIEMMQNVDKNMGNITERWRILSGNSNWEGLLDPLDNDLRRYLIRYGEMTQAARDAFNTNKISKYAGTCRYSKKNFFSRTGIEISNPFKYEVTKYIYATSAVQVPEALFIKSLSREAWSKESNWAGFVAVATDKGKIALGRRDIVIVWRGSVQTLEWFNDFDFIQVSATKIFGEKSDPKVHHGWYSIYTSDDSRSLFNKESARDQVLGEVKRLMEQYKTEEVSITVTGHSMGASMATLNAGDIVFNGINKGFPVTAFLFASPRVGDENFKRTFSKLENLRALRIRNAPDLVPSYPLFGYSDVGVELAIDTRKSGYLKSPGDQSSFHNTDCYLHGIAGTQGSKGGFKLEVERDISLINKYLDALKDEYGVPTSWWVEKNNGMVQQQNGTWILVDHEDDDF

Solyc02g077140 SlPLA1-17

MGCINRGWEVVSRKISSCLIKKKGIENRDNSIAERWEILSGKNNWEGLLDPLDYDLRRYLIHYGQMPQAICDSFNNEKVSKYRGTSRYSKKNLFTRVGLDKNPYEITKYVYAASSKTEETKESNWIGFVAVATDEGKVALGRRDILIAWRGTKTKSEMNEDDKWSLVQPSKIFGENRDNILVHKGFYSVYTCLNEASNFNRTTSARDQVLEEIKRLLKQYSKEEISISVTGQSMGSSLGTLCVEGCAITEWRAYEDVGFELVIDTTKSEYLKKDIFSHFLEVYLHGIAGTHGVEGEFKLEINRDIALVNKQGDFLKEEYGVPSAWWIEKNKGMVQQEDGSWILIDHET*

SlPLA1-17 supplementation

MGCINRGWEVVSRKISSCLIKKKGIENRDNSIAERWEILSGKNNWEGLLDPLDYDLRRYLIHYGQMPQAICDSFNNEKVSKYRGTSRYSKKNLFTRVGLDKNPYEITKYVYAASSKTEETKESNWIGFVAVATDEGKVALGRRDILIAWRGTKTKSEMNEDDKWSLVQPSKIFGENRDNILVHKGFYSVYTCLNEASNFNRTTSARDQVLEEIKRLLKQYSKEEISISVTGQSMGSSLGTLCAIDIVVNEINKEFPVTAFLFSSPRVGEANFKKAYRNLKNLHILRITNVPDPIPKLMERGQVEGCAITEWRAYEDVGFELVIDTTKSEYLKKDIFSHFLEVYLHGIAGTHGVEGEFKLEINRDIALVNKQGDFLKEEYGVPSAWWIEKNKGMVQQEDGSWILIDHET

Solyc02g077150 SlPLA1-18

MYFNCFKPRKNPRETIEKMQNGNKNIAERWRVLSGNNNWEGLLDPLDNDLRRYLIHYGEMVQAIRDAFNNDETSKYAGCSRYSKKNLFSKVGIEISNPFKYEVTKYVYATSSIQVPEAFIIKSLSGEAWSKESNWIGFVAVATDEGEVALGRRDIVIAWRSTVVPMEWFNDFEFIRVSAPTIFGENSDPKVHHGWYSMYTSNDPRSLFNKASARDQVLGEVERLMEQYKTEEVSITVTGHSMGASIATLNAVDMVFNGINKGFPVTAFLFASPRVGDENFNKTFSELENQLRALRVRNIPDIIPHYPFIGYSDVGVELIMDTRKSDYLKSGGDYWTWHNLECYLHGVAGTQGSKEGFKLEVERDISLVNKHMDTLKDEYGVPVSWWVVENKGMVQQQNGSWILMDHEDEDNDF*

Solyc02g077160 SlPLA1-19

MACINKGWEVVSRKISNCLKRKRGIENGENNIAERWEILSGKNNWEGLLDPLDYDLRRYLIHYGQMPQAIYDSFNNEKVSKYRGTSRYSKKNLFTRVGLHRNKYEITKYFYGASSKTEKVKVSNWIGFVAVATDEGKVALGRRDILIVWRGTITVSEWNDDFESSMVQPIEIFRENTDNILVHKGFYSIYTSLNHASNFNRTTSARDQVLEEVKRLMDQYKKEEVSISVTGHSLGSSLATLCAIDIAVSQVNKGFPVTAFLFASPRVGEINFKKACENLKNLHILRITNASDLITKLPDRGQVEGCETDWRVYEDVGFELSIDTTKSDYLKKEINGHILEVYLHGIAGTHGFEGEFKLEMNRDIALMNKADDVLKDEYGVPASWWIEKNKGMVQHENGSWILMDHEDDHN*

Solyc02g077420 SlPLA1-20

MSSMAEKWEELSGKNKWEGLLNPLDVDLRKYIIQYGELAEVTYDTFISDKVSKYAGASRYSMENLFSNVGLDPSKYRVTKYFYATSSIPLPDAFITKSLSREAWSKESNFMGYIAVATDEGKVSLGRRDIVIAWRGTIQTLEWVNDLQFLLIPGPKVFGDGGLLPLFKPLVHHGFYNVYTSESARSNFNKKSARDQVIEEVKRLVEEYKNEEVSITVTGHSLGASLATLNAFDIAYNKINKTSEGKEFPVTAFVFASPKVGDINFVNAFNKLKHLHVMRIHNVLDIVPKYPPLGYFDVGQEIIIDTTKSPYLNLPGDILTWHNLECYLHGVAGTQGIGLLAGFKLEVDRDIALVNKSSGALKSEYLVPANWWTAKNKGMVQQEDGKWVLNDREEYDIVVAEV*

Solyc02g077430 SlPLA1-21

YQVKTKREKKMSSMADKWEELSGKNKWEGLLNPLDVDLRKYIIQYGELAHVTYDTFITEKASKYAGASRY
SMENLFSKAGLDPSKYRVTKYFYATSSIPLPDAFITKSLSREAWSKESNFMGYIAVATDEGKVSLGRRDIVIAWRG
TIQTLEWVNDLQFLLIPAPEVFGKGGLLPLTQPLVHHGFYNIYTSESERSKFNKTSARDQVLEEVKRLVEEYKDDE
VSITVAGHSLGASLATLNAVDIAFNGINKTSEGKEFPVTAFVFASPKVGDVNFVNTFNKLKNLHIMRIDNLLDIVP
KYPPIGYFDVGQEIIIDTTKSPYLKLNPGDPHTRHNLEGYLHGIDGTQGIGPLDGFKLEVNRDLALVNRIWDILKD
EHLVPGAWWVEKHNGMVQQENGKWILMDHEEYEL*

SlPLA1-21 supplementation

MSSMADKWEELSGKNKWEGLLNPLDVDLRKYIIQYGELAHVTYDTFITEKASKYAGASRYSMENLFSKAG
LDPSKYRVTKYFYATSSIPLPDAFITKSLSREAWSKESNFMGYIAVATDEGKVSLGRRDIVIAWRGTIQTLEWVND
LQFLLIPAPEVFGKGGLLPLTQPLVHHGFYNIYTSESERSKFNKTSARDQVLEEVKRLVEEYKDDEVSITVAGHSLG
ASLATLNAVDIAFNGINKTSEGKEFPVTAFVFASPKVGDVNFVNTFNKLKNLHIMRIDNLLDIVPKYPPIGYFDVG
QEIIIDTTKSPYLKLNPGDPHTRHNLEGYLHGIDGTQGIGPLDGFKLEVNRDLALVNRIWDILKDEHLVPGAWW
VEKHNGMVQQENGKWILMDHEEYEL

Solyc02g080340 SlpPLA4

MSWGLGWKRPSDVFHLTLSYGEDEALDESTPTSSRSSSSTSVHTSPFSPSPPSPPAEGQEENNQEELLGFR
VDLDWNVGDDEDQVALKLQSQVMVALPSPQDTVEVEFKDKKENENAAEEDMGEVAVEMRVVKRREPLKGV
MMWRVGSSSQQSDGMGVLSKLIRSNFANGGALGIGEGSPVGCADHWKSVTVVSLCGLGLMVLPVEITQLPLI
ERLYLDNNKLSNLPPELGALKCLKVLAVDYNMLVSVPVELRECIGLVELSLEHNKLVRPLLDFRAMTMLRVLRLFG
NPLEFLPDILPLQKLRHLSLANIRVVADDQLRLVNVQIEMENSSYFIASRHKLSAFFSLIFRFSSCHHPLLASALAKI
MQDEGNRVVVGKDENAVRQLISMISSDNQHVVEQACSALSSLATDVSVAMQLMKSDIMQPIERVLKSAGPEE
VISVLQVLGNLAFASDIVSQKLLTKDVLRSLKLLCAHRNPEVQRLALFAVGNLAFCLENRRILVTSESLRELLLRLTVA
SEQQVSKAAARALAILGENEVLRRAIRGRQVPKQGLRILSMDGGGMKGLATVRILKEIEKGTGKQIHELFDLICG
TSTGGMLAVALGIKLMSLEKCEEIYKKLGKLVFAEPVPKDNEAATWREKFDQLYKSSSQSFRVVIHGSKHSAEQFE
RLLKEMCADEDGDLLIESAIKRIPKVFVVSTLVSATPAQPFIFRNYQYPPGTPEISPAATENLTTAGQGTISDPAQVE
HKRNAFMGSCKHRIWQAIRASSAAPYYLDDYSDDVYRWQDGAIVANNPTIFAIREAQLLWPDARIDCMVSIG
CGSVPMKVRKGGWRYLDTGQVLIESACSVDRVEEALSTLLPLLPDVHYFRFNPVDERCDMELDETDPAVWSKL
EAATDDYIQNTSAAFKNICERLLERPHDEKFSDKKSHQFLKAKNSKTDESSPSLGWRRSVLLVEAPNSADAGRVF
HHVRSLESLCARNGIKLSLFNGISNTQKATPGSTFPTPFASPLFTGSFPSSPLLYSPDIGAHRVGRIDLVPPLSLDGL
QSAKTTVSPPDSPRKHRQLSLPVQSLYEKLKNSPQVGVVHLALQNDTSGSVLSWQNDVFVVAEPGELADKFLQ
SVKFSLLSMMRGRRRKYASVISDISTVADLVRCRPCFQIGGVVHRYIGRQTQVMEDDQEIGAYMFRRTVPSMH
LTSEDIRWMVGAWRERIIIFTGFYGPIQPVIKAFLDSGAKAVICPSSEPDEVQLSTFHGSGDFNSFDNGKFEIGEE
EAEDDDTEPTSPASDWDDSEPDESEGRSQFFWDDDEGELSQFICQFYESLFQGGSRIGAALQQARASHRSLRY
SCHLPSI

Solyc02g090490 SlpPLA5

MGRMVLIAAAMTLFVTLQVLQPPLVVSAATKGKTVTVLSIDGGGIRGIIPGTLLAFLESKLQELDGPNARIA
DYFDVVAGTSTGGLVTTMLTAPNKDNRPLYQAKDISNFYMQHGPQIFPQSRRNSFVRRITNLFGGPKYDGIYLR
TIINSILGNLTMKQTLTNTVIPTFDIKRLQPIIFSTADAKANISKNAQLSDVCLSTSAAPTYFPVHSFETKDAQGKTR
TFDLVDGGVAANNPTLMAITYVSKQIMTGNFQYEGMKNMDCNKMLVLSLGTGIGKQEEKYNATVASRWGM
VGWVYNNGATPLIDIYGDASADMVDIHTSTMFQTLGSEKNYIRIQDDNLTGEAASMDIATTQNMETLVQIGN
DLLKKPISRVNLETGRYEPVVGEGTNEAAIVRFAQLLSEERKLRIN

Solyc02g090630 SlpPLA6

MEREVEGESSRVAMPPPNTGKLITILTIDGGGIRGIIPGVILAYLESQLQELDGEDARIADYFDLIAGTSTGGL
VTAMLAAPNKDKRPLYAAKDITPFYIEHSPKIFPQISCCSGLFAGAINLAKMINGPKYDGKYLHELIKRLLGGTRLH

DTLTAVVIPTFDIKTLQPVIFSSYEANSKPELNAELADICISTSAAPTFLPAYCFNTKDAQNEDREFNLIDGGVAANN
PTLVAIGEVTKQTLMKHEDLFPIKPMDYGRFLVISLGTGNAKNEGKYNAKMASKWGLLSWLTHDNSTPIVEAFN
QASADMVDYHNFVVFKALHSDDKYLRIQDDTLTGNLASVDISTKENLQGLIKVGEELLDKPTSKINLDKGVYEAV
ENGGTNKEALRRFAKKLSDERKFRQANAGQ*

Solyc02g090640 SlpPLA7

MENSAAVGVAMPPPNKGKLITILSIDGGGIRGIIPGVILAYLESQLQELDGEDARIADYFDLIAGTSTGGLVT
AMLAAPNKAKRPLYAAKDITPFYLEHSPKIFHQIGGPFGGAINLTKMLNRPKYDGKYLHTLIKGLLGGTRLHDTLT
AVVIPTFDIKELQPV

SlpPLA7 supplementation

MENSAAVGVAMPPPNKGKLITILSIDGGGIRGIIPGVILAYLESQLQELDGEDARIADYFDLIAGTSTGGLVT
AMLAAPNKAKRPLYAAKDITPFYLEHSPKIFHQIGGPFGGAINLTKMLNRPKYDGKYLHTLIKGLLGGTRLHDTLT
AVVIPTFDIKELQPVIFSSYETKSKPVLDAELADICISTSAAPTFLPAHNFKTKDAQNNENEFNLIDGGVAANNPTLI
AIGEVTKQVLMKHEDLFPIKPLDYGRFLVISLGTGNAKNEGKYNAKMASKWGLLSWLTHDNSTPIIEAFNQASA
DMVDFHNFVVFKALQSDDQYLRIQDDTLTGNLASVDIATKENLQGLVKVGEKLLDKPTSKINLDKGVYEAVENG
GTNKEALRRFAKILSDERKFRQSNAGQ

Solyc02g090660 SlpPLA8

MERYGERITVLSIDGGGVRGIIPGTILSFLESKLQELDGQEARLADYFDIIIAGSSTGGLMATMLTTWPSSLC
SQVKLWSIIFYVVINCSIASALIRMLWGPKYDGKYLRNLIRRILGNRRLHETITHLLIPTYDIKTLEPQIFSSYEVLKFT
SLHLFLQKENSFSSILTNAGHGFDALLSDICVGTSSAPVYLPAHLFKTKDYHGNDRPYRWRHCSQ*

Solyc02g090920 SlPLA1-22

MERVSSNKYLQLKPDETSLLDLIRILFSGDLKSKKFIESWTEKEIAFERRRYIFLSVSVQKAFHFISKPLFFFGST
SESCLNLLASYQSLPTLLLRVLQGKVEMPDKESASYLSTIGFIDQRVNSHKKFKPGDKTYIVALSAVASKVAYENKA
FIRATVEDQWKMDLLGYYDFWNEYHEKKSTQGFLFHDKTRCPGVIIVAFRGTEPFNSYDWSTDFDISWYEFQD
MGKVHSGFMKALGLQKDKKWLPNITQDDQRPLAYYTIREKLRDIFQKNKQTKFVLTGHSLGGALAVLFAAVLAF
HDETFILERLEAIYTFGQPRVGDAEFGDFMKENFRNYGVEYYRFVYSHDIVPRLPYDDSIMLFKHFGTCLYYNSTY
EGKIVSEEPDRNYFSVRSLISKRVDALWELACRLMVLKSILMQFVWEMLICSPMLLYHLDNSRLLWCLIGNWNL
FLH

SlPLA1-22 supplementation

MERVSSNKYLQLKPDETSLLDLIRILFSGDLKSKKFIESWTEKEIAFERRRYIFLSVSVQKAFHFISKPLFFFGST
SESCLNLLASYQSLPTLLLRVLQGKVEMPDKESASYLSTIGFIDQRVNSHKKFKPGDKTYIVALSAVASKVAYENKA
FIRATVEDQWKMDLLGYYDFWNEYHEKKSTQGFLFHDKTRCPGVIIVAFRGTEPFNSYDWSTDFDISWYEFQD
MGKVHSGFMKALGLQKDKKWLPNITQDDQRPLAYYTIREKLRDIFQKNKQTKFVLTGHSLGGALAVLFAAVLAF
HDETFILERLEAIYTFGQPRVGDAEFGDFMKENFRNYGVEYYRFVYSHDIVPRLPYDDSIMLFKHFGTCLYYNSTY
EGKIVSEEPDRNYFSVRSLISKRVDALWELVRSFLLPYLYGAEYRENLLLQALRLYGLVFPGMPAHGPQEYINAICL
GDANLFTNVAVSS

Solyc02g090930 SlPLA1-23

MASNSCNKSFCSNYMLLKPEECSVLDLAKLLLSGKKLGQKDFIDCPNEEMTKEPLSRRWVIFLSILVQKILL
ATAKPLAGLGNAIEYWLNLQNVNGGFFRLLFKSLICKAVSPDKESAAYLSFIGNLDKRVDLDFSKNVGGRRRYDE
GISMMAAKAAYENKAYVETTVNHHWKMDLVGSFDFWNDYQEKATTQAFVLQDKSVDPELIIVAFRGTEFFNS
DDWISDFNLSWYDIPGMGKVHAGFMKALGLQKSLGWPKNIVQTDTNNINQPPPAYYFLRKLLKQLLEKNEKA
RFLVTGHSLGGALAILFPAILAFHEESWLLKRLGGIYTFGQPRVGDESFIEYMNGQLTKHEVPYYRVVYSNDMVP
RLPFDNSTFLFKHFGTCIYYNSLYREKILVEEPDKNGSFLLMFIPKMLNAAWELIRSCILPCVHGRKYQEGGLLLC
MRVIGLLFPGMPAHCPQDYVNSTRLGSSDHSTEDVAKYQKIGIVPA*

Solyc02g090940 SlPLA1-24

LICRMASSSCNKSFCSNYMLLKPEECSVSDLAKILFSSKNLGQKEFVDVDIPNEELNMTKEPLSRRWVFFISI
LVQKVLIKISKPLAGFGNTIEYWLNLQNVNGGFFRLIFNSLRGRAVIPDKESAEYLSFIGNLDKRFELDFSKIVEEVG
GCKRYDEAISIMAAKAAYENKAWIQNTVNQHWKMDFVGSFDFWNDYQEKATTQAFVLQDKNVDPELIVVAF
RGTEFFNSDDWISDFNLSWYDIPGMGKVHAGFMKALGLQKSLGWPKEIEQTDTNNINQPSPAYYFLRKLLKQL
LEKNENAKFVVTGHSLGGALAILFPAILAYHEESWLLKRLGGVYTFGQPRVGDENFVDYMKEQLAKYEVPYYRV
VYSNDMVPRLPYDNFTFMFKHFGTCIYYNSLYKKQILGEEPDKNGLALLLFLPKMLNAGWELIRSCILPCVNGW
KYQEGGLLLFMRVVGLLLPGIPAHCPQDYVNASRLGSLKTSQSSKRLV*

SlPLA1-24 supplementation

MASSSCNKSFCSNYMLLKPEECSVSDLAKILFSSKNLGQKEFVDVDIPNEELNMTKEPLSRRWVFFISILVQ
KVLIKISKPLAGFGNTIEYWLNLQNVNGGFFRLIFNSLRGRAVIPDKESAEYLSFIGNLDKRFELDFSKIVEEVGGC
KRYDEAISIMAAKAAYENKAWIQNTVNQHWKMDFVGSFDFWNDYQEKATTQAFVLQDKNVDPELIVVAFRG
TEFFNSDDWISDFNLSWYDIPGMGKVHAGFMKALGLQKSLGWPKEIEQTDTNNINQPSPAYYFLRKLLKQLLE
KNENAKFVVTGHSLGGALAILFPAILAYHEESWLLKRLGGVYTFGQPRVGDENFVDYMKEQLAKYEVPYYRVVY
SNDMVPRLPYDNFTFMFKHFGTCIYYNSLYKKQILGEEPDKNGLALLLFLPKMLNAGWELIRSCILPCVNGWKY
QEGGLLLFMRVVGLLLPGIPAHCPQDYVNASRLGSLKTSQSSKRLV

Solyc03g025510 SlPLA1-25

MSVLCGLPPLLECVYGVACARWAWKRCLHSAGHDSENWSVATAEEFEPVPRLCRYILGVYEDDLRQPQ
WEPPEGYGINPDCLIVKKNYPDTGGRAPPYLLYLDHDHADIVLAIRGLNLAKESDYAVLLDNKLGKRKFDGGYVH
NGLLKAAGLVLNAECEIMKQLLEKYPNYTLTVTGHSLGSGVAALLTMVVAQNLDKLGNIDRKRIRCFAIAPARCM
SLNLAVRYADVINSIVLQDDFLPRTATPLEDIFKSLFCLPCLLCLRCMRDTCISEEKMLKDPRRLYAPGRLYHIVERK
PFRCGRFPPVVKTAVPVDGRFEHIVLSCNATSDHAIIWIEREARRALELMQERDHVKEIPAKQKMERQQTLTRE
HNEEHKAALQRAVTLAVPHAFSPSHYGTFDELEEEQSDTSVRDSSPGSSSRSKTKDNWDELIERLYEKDESGHKI
LIRSRSQ*

SlPLA1-25 supplementation

MSVLCGLPPLLECVYGVACARWAWKRCLHSAGHDSENWSVATAEEFEPVPRLCRYILGVYEDDLRQPQ
WEPPEGYGINPDCLIVKKNYPDTGGRAPPYLLYLDHDHADIVLAIRGLNLAKESDYAVLLDNKLGKRKFDGGYVH
NGLLKAAGLVLNAECEIMKQLLEKYPNYTLTVTGHSLGSGVAALLTMVVAQNLDKLGNIDRKRIRCFAIAPARCM
SLNLAVRYADVINSIVLQASSTCITLNILNHSRWILLVWKHDDFLPRTATPLEDIFKSLFCLPCLLCLRCMRDTCISEE
KMLKDPRRLYAPGRLYHIVERKPFRTCLCMWKTRSMMVPAISTNTATRCGRFPPVVKTAVPVDGRFEHIVLSCN
ATSDHAIIWIEREARRALELMQERDHVKEIPAKQKMERQQTLTREHNEEHKAALQRAVTLAVPHAFSPSHYGTF
DELEEEQSDTSVRDSSPGSSSRSKTKDNWDELIERLYEKDESGHKILIRSRSQ

Solyc03g044710 SlpPLA9

MLTHTVIPAFDIKRLQPIIFTTVDGRTNGLKDALLSDICLSTSAAPTYFPVHYFETRDAAGRIHTFDLIDGGV
AANNPTLMAITHISKEIMMGSLKYEEMERMDSKKMLVLSLGTGIGKHQGKYNAASATKWGLLGWVYNNGDT
PIIDVYSDASADMVDIHVSTMFQTLHNEKNYLRIQDDNLIGDAASMDIATTENMETLVQIGNNLLKKPVSRVNL
ETGQYEPVHGEGTNEEALIRFANLLSHEKKLRS*

SlpPLA9 supplementation

MCIEKEWVLKRMYHFEGRIARRDGYYISRDVSRVAMVTIIEGRVVRRDRCMDRYVPHGSRTDRQRAICL
QVGIVCGATNEKKLVTVLSIDGGGIRGIIPGTLLAFLESKLQELDGPNARIADYFDVVAGTSTGGLITTMLTAPNRD
NRPLYAAKDISTFYMEHGPHIFPQTRRRNFVNNVAHLFGGPKYDGEYLRLLVDSILGNLTIKQMLTHTVIPAFDIK
RLQPIIFTTVDGRTNGLKDALLSDICLSTSAAPTYFPVHYFETRDAAGRIHTFDLIDGGVAANNPTLMAITHISKEI
MMGSLKYEEMERMDSKKMLVLSLGTGIGKHQGKYNAASATKWGLLGWVYNNGDTPIIDVYSDASADMVDI
HVSTMFQTLHNEKNYLRIQDDNLIGDAASMDIATTENMETLVQIGNNLLKKPVSRVNLETGQYEPVHGEGTNE
EALIRFANLLSHEKKLRS

Solyc03g083370 SlPLA1-26

MASEREIFSLSGPLYLIAIDWSNTCHRRSIAASLVQGVYILERDRQQNRQGFNALAPPWWEFFHFQLIQVL
VDNEDQSYFGAIYEYKFPNSHFHNKTKNDIQNQNQNPPKYVIAFRGTITKKGNRSQDFKINLTLIRDNLHNCSR
FHIGLQVVHNIVQNHEISDIWLTGHSLGSSIALLIGRNMVKTGIDLETYLFNPPFTSLPVEKITKNEKLKHGIRITHS
VLTAGLASAVNICKSKSINKSESITLLSSWIPYLFVNPSDPICAEYVGYFEHREKMAAIGKGEIGRIATQNSIRSIIGN
AIGKDQLEPSHLLPSANVAINLSPSPDFKRAHGIHQWWNPDVQCNYKLYQFR*

Solyc03g122280 SlPLA1-27

MATATLATAAGAAALLYYTLNKKLQSSPTTDDDDDECGSSGQDHALLGVNRVSNRLIQAPATWLETIATLS
ETLRFTYSETLGKWPIGDLAFGISFLLKQQGNIHVSSIFCVEDSAQLKGSDIAAELKCLLRLLTVCWHFSKKPFPLFL
EETGYSQESVLLQEPKAGILKPAFTILADHRSRSFLLVIRGTHSIKDTLTAATGAVVPFHHTVVHEGGVSNLVLGYA
HCGMVAAARWIARLATPCLLKALSIYPEYKLKIVGHSLGGGTAAILTYVLREQKELSTATCVAFAPAACMTWELAE
SGCEFITSVINGADLVPTFSAASVDDLRSEVTTSAWLNDLRNQIEHTRILSTVYRSASALGSRLPSIATAKAKVAGA
GAILRPVSSGTQVVMKRAQSMAQAALSRPAMQLSSWSCMGPRRRSTAIQVNSEERQISRGTSSGDNSEAFIV
ESETRRTSVMELPVSSTEGVSWNAEIDQSFADGINIHSGLDSDLDDSEHVTHGPEDRMTEVEMWQQLEHELY
DQSEGETDVAKEIREEEEAAIAETGQSSSESSVPKTKEVHRFFPPGKIMHIVTLLSEEVDHESDSDTLSEDHNQPK
DTKVGIFLTPRSLYSKIRLSQTMISDHFMPVYRRHIEKLIRDLENNDACEL*

SlPLA1-27 supplementation

MATATLATAAGAAALLYYTLNKKLQSSPTTDDDDDECGSSGQDHALLGVNRVSNRLIQAPATWLETIATLS
ETLRFTYSETLGKWPIGDLAFGISFLLKQQGNIHVSSIFCVEDSAQLKGSDIAAELKCLLRLLTVCWHFSKKPFPLFL
EETGYSQESVLLQEPKAGILKPAFTILADHRSRSFLLVIRGTHSIKDTLTAATGAVVPFHHTVVHEGGVSNLVLGYA
HCGMVAAARWIARLATPCLLKALSIYPEYKLKIVGHSLGGGTAAILTYVLREQKELSTATCVAFAPAACMTWELAE
SGCEFITSVINGADLVPTFSAASVDDLRSEVSFWIYLTEFLWLIYVQIVILLSIEYAAGEPFEVTTSAWLNDLRNQIE
HTRILSTVYRSASALGSRLPSIATAKAKVAGAGAILRPVSSGTQVVMKRAQSMAQAALSRPAMQLSSWSCMGP
RRRSTAIQVNSEERQISRGTSSGDNSEAFIVESETRRTSVMELPVSSTEGVSWNAEIDQSFADGINIHSGLDSDLD
DSEHVTHGPEDRMTEVEMWQQLEHELYDQSEGETDVAKEIREEEEAAIAETGQSSSESSVPKTKEVHRFFPPG
KIMHIVTLLSEEVDHESDSDTLSEDHNQPKDTKVGIFLTPRSLYSKIRLSQTMISDHFMPVYRRHIEKLIRDLENND
AFSLGRVCDDHINENDKA

Solyc03g123750 SlPLA1-28

MEVDGGEWTGNSRFLIVNYQDGGILDLMRFLLSANKENAHKFLHYSDGGTPVVAEELTRVHDDTTAGD
HRWVIFVSVIVRKLIAIFGKPMEWFGYLLDFILNLLSLNGNFFGLFYNILHGKVVMPQRGSETFISAIGHLDGRIDL
YRTETLTKEIGEPDFWQKDIQLGIGHRALMDLCMMASKLAYENAKVVQNVVNIHWKMHFVDFYNCWNDFE
KEMSTQVFILCDKPKDANLIVISFRGTEPFDADDWITDFDYSWYEIPKLGKVHMGFLEALGLGSRAKVSTFHEQL
FMNNQNFTKLENDATIAPSESSESSTMFSDSDAHSVSDQSPESDRPTDAGSKKFKLDMPERTAYYVVRSKLKRL
LNEHKNAKFVVTGHSLGGALAILFPTILVLHEEMNVMERLLGIYTYGQPRIGNRQLGRFMEAHLEHPVPKYFRV
VYSNDLVPRLPYDNKTFLFKHFGICQYYNSLYVEQNVDEEPNRNYFGLRFLIPLYLNAGWELIRSFTMGHIYGAEY
EECWESVVLRVLGLFLPGISAHSPVDYVNSVRLGKERSTQMSSF*

Solyc04g078800 SlPLA1-29

MGCNKEFCKDYFELKAEEASCYDLFRIFYSCELEKKKFVDASQGTNKIQGIQRRWIVFASVSMQRFLISVRK
PMNIIGSKVELLLNYPSCNGGLLKLFFNLILGEVVRPDMKSENFMSMIGQLDWRVDLDKTKNVGDNYYGPSLA
IMAAKLSYENEAFTKKVITNNWQMDFIKFYSFWNGKLHAGFMKAMGLQKNKGWPKEIDESSDQKLFAYYQIR
KELKMILIKNEKAKFILTGHSLGGALAVLFAAILILHEEEWLLDRLEGVYTFGQPRVGDPQFGNFMKDKFNKYDV
KYYRHVYSNDMVPRLPYDDTTFFKHFGSCLYYNSLYSGKVVEEEPNKNYFSVLWFLPMLLIAVYEFIRGFILPWTK
GSHYREDWLQKMFRVVGLVIPGLSAHTTIDYVNLTRLGSVLHLPRSASHDQDRLKDD*

SlPLA1-29 supplementation

MGCNKEFCKDYFELKAEEASCYDLFRIFYSCELEKKKFVDASQGTNKIQGIQRRWIVFASVSMQRFLISVRKPMNIIGSKVELLLNYPSCNGGLLKLFFNLILGEVVRPDMKSENFMSMIGQLDWRVDLDKTKNVGDNYYGPSLAIMAAKLSYENEAFTKKVITNNWQMDFIKFYSFWNAYQEVYTTQAIMFQDKVEDSNLVVVAFRGTIPYNADHWITDVDLSWYELEGVGKLHAGFMKAMGLQKNKGWPKEIDESSDQKLFAYYQIRKELKMILIKNEKAKFILTGHSLGGALAVLFAAILILHEEEWLLDRLEGVYTFGQPRVGDPQFGNFMKDKFNKYDVKYYRHVYSNDMVPRLPYDDTTFFKHFGSCLYYNSLYSGKVVEEEPNKNYFSVLWFLPMLLIAVYEFIRGFILPWTKGSHYREDWLQKMFRVVGLVIPGLSAHTTIDYVNLTRLGSVLHLPRSASHDQDRLKDD

Solyc04g079210 SlpPLA10

MNAKLSDICIGTSAAPTYLPAHNFQTQNEDGKFHEFNLIDGAIAANNPTLIAINEATKQIFEQTPYFKNFKEMKPTNYGKFLVLSIGTGSAKLEHKYDAKIASNWGIFGWLTGGGSNPIIDAFADASDDMVDYHISVVFQSVHSQKHYLRIQDDTLSGADSTVDISTKENMNKLVEIGTNLLKKPVSRVNLQTGLFEQLNKDGGTNEEALKEFAKLLSEEKKIRNFKSSPTDES*

Solyc04g079230 SlpPLA11

KMSSSSSSKLQSPINGKFITILSIDGGGIRGLIPATILEYLESQLQELDGKDARLADYFDIITGTSTGGLVTAMLTAPNKENRPLFAAKDIKPFYLEHGPNIFPQNNMILIGSIIKGWKFLTGPKYNGKYLHQVIGEIRGD*

SlpPLA11 supplementation

MNAKLSDICIGTSAAPTYLPAHNFQTQNEDGKFHEFNLIDGAIAANNPTLIAINEATKQIFEQTPYFKNFKEMKPTNYGKFLVLSIGTGSAKLEHKYDAKIASNWGIFGWLTGGGSNPIIDAFADASDDMVDYHISVVFQSVHSQKHYLRIQDDTLSGADSTVDISTKENMNKLVEIGTNLLKKPVSRVNLQTGLFEQLNKDGGTNEEALKE

Solyc04g079240 SlpPLA12

MKNVMLSSSGSLKLQPPTYGKLITILSIDGGGIRGIIPAIILEYLESQLQELDGEDARLADYFDIIAGTSTGGLVTAMLTAPNKDKRPIFAAKDIKPFYLEHGPKIFPQKGMLFFGSIIKTLKSLIGPKYNGKYLHQVIKEKLGETHLHETLTNVVIPTFDIKNLQPTIFSTFEAKEKPLMDAKLSDICISTSGAPTYLPAHYFKTQDRDDNFHEFNLVDGGVAANNPALVATSQVTKQIMAGNPDFFPIKPIDYGRFLVISVGTGSAKVEQKYNAKIASKWGILGWLLNGGSTPIVDVFTQASGDMVDFHISVVFQALHSEENYLRVQDDTLSGTDSSVDISTKENMNKLVEIGTNLLKKPVSRVNLQTGLFEECKKDDTNEETLKRFAKLLSEEKRLRDSKSPHTSKTSE*

Solyc04g079250 SlpPLA13

MERKTSQIQPPTYGDLITVLSIDGGGIRGIIPATILSFLESQLQELDGKEARLADYFDVIAGTSTGGLVTAMLTAPDENNRPLYAAKDITPFYLEHCPKIFPQKKCGLFAPIGNIVQTLIGPKYDGKYLHQVVKEKLKDTRLSNTITNVVIPTFDIKKLQPTIFSTYETKRAACYDAKLSDICISTSAAPTYLPAHYFKVEDTKGNFKEHHLIDGGVAANNPGLIAISEVSKEIFKNNPDFYPIKPMEYGRFLVISLGTGAAKYEQKYNSSMAAKWGILEWLLNKGSNPLIEVFTQSSADMVDYHNSVVFQALRSEDSYLRIQEDELSGTEASVDVATKENLERLVEIGENLLKKPLSRVNLETGLSEPIPKGGTNEEALKRFATLLVNERRLRESKSPLIIKKA*

Solyc04g079260 SlpPLA14

MERKTSQIQPPTYGDFITILSIDGGGIRGIIPATILTFLESQLQELDGKNARIADYFDVIAGTSTGGLVAAMLTAPDENHRPLYAAKDITPFYLEHCPKIFPQKKCGLFAPIGKMVQALIGPKYDGKYLHEVVKEKLKDICISNTITNVVIPTFDIKKLQPTIFSTYETKRSTCCDAKLSDICISTSAAPTYLPAHYFKVEDGKGNVKEHHLIDGGVAANNPGLIAISEVSKEIFKNNPDFFPIKPMDYGRFLVISLGTGAAKCEQKYNSLMGAKWGIVDWLIHKGSTPLVEVFTQSSADMVDYHNSIVFQALRSENSYLRIQEDELIGTEASVDVATKKNLERLVEIGEQLLKKPLSRVNLETGLSEPTPKGGTNEEALKRFARLLVNERRLRELRSPLIKKASK*

Solyc05g051280 SlPLA1-30

MSVACGVECVLVVGCIRWAWKRCTYIGNNDSATWPEATCEEFEPVPRLCRTILAVYEPNLHKPKFPPPGGYRLNPDWVVKRVTYEQTSGRAPPYLIYCDHEHKEIVLAIRGLNLLSESDYKVLLDNRLGKQMFDGGYVHHGLLKSAIWVLNNESETLSKLWIDNGRSYKMIFAGHSLGSGVASLLTIIVVNHKDRLGGIPRNLVRCYAVAPARCMSLNLA

VKYADVIHSVILQDDFLPRTATPLEDIFKSIFCLPCLIFLICLRDTFIPEGRKLRDPRRLYAPGRMYHIVERRFCRCGRY
PPDVRTAIPVDGRFEHIVLSCNSLSDHGIIWIEKESEKAFARLKEASAETTTSPPKVQKFERLKTLEKEHKDAIERAV
SLNIPHAVGVGEEESSTQKEEEEPIEDITFEASQKHDEDASTSKAQGSDARTSLHEVVETLLNRDESGKLQLKRES
TGPE*

SlPLA1-30 supplementation

MSVACGVECVLVVGCIRWAWKRCTYIGNNDSATWPEATCEEFEPVPRLCRTILAVYEPNLHKPKFPPPGG
YRLNPDWVVKRVTYEQTSGRAPPYLIYCDHEHKEIVLAIRGLNLLSESDYKVLLDNRLGKQMFDGGYVHHGLLK
SAIWVLNNESETLSKLWIDNGRSYKMIFAGHSLGSGVASLLTIIVVNHKDRLGGIPRNLVRCYAVAPARCMSLNLA
VKYADVIHSVILQKDAIFVCGIHTWMISCQEQPHHLKIYLNPSSGINAFNSLPCLIFLICLRDTFIPEGRKLRDPRRL
YAPGRMYHIVERRFCRCGRYPPDVRTAIPVDGRFEHIVLSCNSLSDHGIIWIEKESEKAFAKYAITHHLDYMLHIV
VLECLFSIKIFNFKIPEEHSSRTSIYLHVCKSLSFSFVVTSTVPQSRIGWGRVNESSCVERLILLRKTSMQRLKEASAE
TTTSPPKVQKFERLKTLEKEHKDAIERAVSLNIPHAVGVGEEESSTQKEEEEPIEDITFEASQKHDEDASTSKAQGS
DARTSLHEVVETLLNRDESGKLQLKRESTGPE

Solyc05g053910 SlPLA1-31

MELVLPISKCSTLSKRVVARSFKITNSGLGHCSVGHRAVVGLRDRWMEFQGIKNWEGLLDPLDDDLRKEI
LRYGEFVEAAYRCFDFDMSSPTYATCLYPKSSMLTDSGLDKTGYKVIKNLYATCVVQMPRWTKKTFPNLASPRSS
WIGYVAVCDDEKEITRLGRRDVVIAYRGTATSSEWLENFRATLTCLPDDMTTFDENYDQPMVQSGLLNLYTTNT
QCDQSLQDTIREEISKILDKYNDEPLSITITGHSLGAALATLTACDITTKFSNAPIVSVVSFGGPRVGNKSFRCLLEK
NGTNILRIVNSDDPITKVPGFVIDDDIDDMAESHVASTGMPSWLQKFMEDTQWVYAEVGKELRLSSKGDIATC
HDLKTYLDLVNNYENDTSLA*

SlPLA1-31 supplementation

MMRLSSGNNLKTNCNIVFTKQHMELVLPISKCSTLSKRVVARSFKITNSGLGHCSVGHRAVVGLRDRWM
EFQGIKNWEGLLDPLDDDLRKEILRYGEFVEAAYRCFDFDMSSPTYATCLYPKSSMLTDSGLDKTGYKVIKNLYAT
CVVQMPRWTKKTFPNLASPRSSWIGYVAVCDDEKEITRLGRRDVVIAYRGTATSSEWLENFRATLTCLPDDMTT
FDENYDQPMVQSGLLNLYTTNTQCDQSLQDTIREEISKILDKYNDEPLSITITGHSLGAALATLTACDITTKFSNAPI
VSVVSFGGPRVGNKSFRCLLEKNGTNILRIVNSDDPITKVPGFVIDDDIDDMAESHVASTGMPSWLQKFMEDT
QWVYAEVGKELRLSSKGDIATCHDLKTYLDLVNNYENDTSLA

Solyc05g053920 SlPLA1-32

MRLSSGNICKSKCNILFMTKQHMRLVFPISKCFKLSKHGARRFKITCSWNQGSNHFCVADRAVVGLRDR
WMEFQGIKNWEGLLDPLDDDLRKEILRYGEFVEAAYRCFDFDMSSPTYATCLYPKSSMLSDCGLDKTGYKVVK
NLCATCVVQMPRWTKKMFPNLASPRSSWFGYVAICDDEKEIARLGRRDIVIAYRGTATSSEWLENLRATLTCLP
DDMTTFDENYDQPMVQSGLLNLYTTNTQCDQSLQDTIREEISKILDKYNDEPLSITITGHSLGAALATLTACDITT
KFSNAPIVSVVSFGGPRVGNKSFRCLLEKNNTNILRIVNSDDPITKVPGFVIDDDIDDMAESHVASTGMPSWLQ
KCMEDTQWVYAEVGKELRLSSNDLCQQIINKGNVAMCHDLKTYLYLLDNFVKVDHKREKPIEKSGWPSD*

Solyc05g056030 SlpPLA15

MESNIISDMRLEPSIDTDKLSYEIFSILESKFLFGYDDQKLWVPKNLPSPVEDGKNDGVLTSTATENIQAIKN
QRGKICILSIDGGGMRNILSGKALAYLEQALKVKSGNPEARIADYFDVAVGSGVGGIFTAMLFSTKDQNRPVFQ
AEDTWKLLEEQGRRIYPSKGSTSAGNGFLRRVLRGGATRSDASGLEKVMKEAFMDKKTGRSLTLKDTLKPVLIPC
YDLSSTAPFLFSRADAFESESFDFRLWEVCRATSAEPGLFEPVCMKSVDGKTGCVAVDGGLAMSNPTAAAITHV
LHNKQEFPFVRGVEDILVLSLGTGQLLEGSFQYEHVKKWKAKDWAKPMARISGDGAADMVDHSVAMAFGQC
RSSNYVRIQANGSSFGRCGVNIDADPSPNNVKKLVGIADEMLKQKNVESLLFGGKKIAEQSNFQKLDWFAGEL
VQEHQRRSCRIAPTVAFKQHLTNQEK*

Solyc06g054550 SlPLA1-33

MEGRILLKGVIFMCLIACSTAREFKVKDQGAIYNHTLATILVEYAASVYVSDLTELFTWTCPKCNDLTKGFQI

LELIVDVKRCLQAFVGVAPNLNAIVIAFRGTQGTSIQNWIADLYWKQLDIEYPGMEDAMVHHGFYSAYHNTTL
RPGVLSAVESAKEQYGDIPIMVTGHSMGGAMAAFCGLDLTVNYGSRNVSVMTFGQPRIGNAAFASYYSKWV
PNTIRVTHEHDIVPHLPPYYYYFPQKTYHHFPREVWLHNLGFGLLSYTVEKVCDDSGEDPSCSRSVTGRSIKDHL
TYYGVRLGGEESGFCKIVMDDRLAAYSKVDIDGNVHLSRDFSASVLRMNVESNEEGWSI*

SlPLA1-33 supplementation

MDVSSETLGKSKILCKLIVVLSYRDGQDSYLRLSFLAVEFKVKDQGAIYNHTLATILVEYAASVYVSDLTELFT
WTCPKCNDLTKGFQILELIVDVKRCLQAFVGVAPNLNAIVIAFRGTQGTRFLSTLGCAHHPCGLMFNLTVCIQN
WIADLYWKQLDIEYPGMEDAMVHHGFYSAYHNTTLRPGVLSAVESAKEQYGDIPIMVTGHSMGGAMAAFCG
LDLTVNYGSRNVSVMTFGQPRIGNAAFASYYSKWVPNTIRVTHEHDIVPHLPPYYYYFPQKTYHHFPREVWLH
NLGFGLLSYTVEKVCDDSGEDPSCSRKRDIKDKTKNKVIKGLLFGVAQWFELGTSMLEVSSSKCLASKSKGFAFW
VELVAPGLPSAGYLSYVPNAILICETCPVTKGEALLAPFPLLIVLCIVENSRNFASALGLFANFLKYSRSVTGRSIKDH
LTYYGVRLGGEESGFCKIVMDDRLAAYSKVDIDGNVHLSRDFSASVLRMNVESNEEGWSI

Solyc06g060870 SlPLA1-34

MQIGTALPNLHLLQPQGVNFKCNAQTVKTISSSTEMTKKHISNLEKLLQKTNPIDSSRPVIEPSSNRLIENR
RKNLLEGLNLANIWPDEMRKAAEDMSPRHLNRLKRLLSSKSMEYSPRNNLANRWREYHGSNNWLGLLDPLD
ENLRRELVRYGEFIQSAYHCFHTDPATSADEVLPERHVALPDRSYKVTKSLYATSSIGLPKWVDDVAPDLRWMTQ
RSSWIGYVAVCDDRSEIQRMGRRDIVIALRGTATCLEWAENFRDLLVEQNDNNDEGVVQSKVECGFLSLYKTSD
HRVPSLAESVVNEVQRLIEKYKGEPLSITVTGHSLGAALSLLVADDLSTCVPNAPPVAVFSFGGPRVGNRGFADRL
NDNNVKVLRIVNNQDVITRVPGMFVSESLDKKLRESGAGRVLEMLDCRMPWAYSHVGTEFRVDTKMSPFLKP
NADVACCHDLEAYLHLVDGFTASNCPFRPNAKRSLVRLLNEQRSNFKRLYTSKGKDLTINLDREHNFPTSSCLPSP
SS*

Solyc06g071280 SlPLA1-35

MVKIGEGIEVRDELIKKTCNLTMEAHNLSPGKPYIYKKINGSTDVVFAFAGTLSSDGWYSNTSFGEKEINTT
LFPSLRSVGTDEVAKVNEVFATRFEEILDKSSLKNEVEKAMLEGRQVVFAGHSSGGAIAILAALWCLECCRTRPN
GDMLLHPYCMTFGSPLVGNKIWSHALRRENWARYFLHFVMKYDVVPRMMLAPLSSIQELLQVISPFINPKSQY
YQHEAVARSSHASNFFMTVMRSASSVASYDACNLKGCTNLLLETVSNIVQLSPYRPFGTYIFCTGNRKLVVVENP
DAVLQLLFYSSQLSSEAEAAVVVPRSLNDHLLYKNEMQDSLEMQDVLHLNNLTDIPLSSNVDPSMNSALNDLGL
STRARLCLRAAGEWEKQKKKNEEKIEQNKRSIRDALSKIQEYQTKCDIRKVGYYDAFKIQNTDDDFNANVRRLEL
AGIWDEIIEMLKRYELPDSFEGRRDWIELGTQFRRQVEPLDIANYYRHLKNEDTGPYLIRARPKRYRFTQRWLEH
FDRVQAGARSESCFWAEVEELRNKPFAQVQDRVLNLETAANGWIQSSLLGDDIFFPESTYTKWWKTLPPQHK
QASWVSRKITP*

Solyc06g083920 SlPLA1-36

MANRSLKRFLNNIEFSSGISRKHVSNQRSVYNILNRCQSNYSNTDGHKPEEKSISNGRERSKYDIDDGDFH
DNKWKLELDWLSKAVEPAVQLCRWALSTGNGNGDKLPPTNKSLAEIFASIQRSKLGLQDWSLTDLTIGLYLIYLQ
QASTSPIEDVKGEQIYSDLIVQDLIYHTELAKGSYKDCTAALSRNCMLRESNVVKFIKNSSVLRPGYYIGIDKRKKL
VVLGIRGTHTVYDLITDIVSSSHEEITLEGYSTHFGTSEAARWFLTHEMGTIRNCLEKHKGFRLRLVGHSLGGATAS
LLAIMLRNMSARELGFSPDIVSAVGYATPPCVSRELAESCSEYVTTVVMQDDIIPRLSIASLTRLRNEILQTNWVS
VLQKEDWRGVVDLFTNAKQVVSSVQDVARRLADYAKLRSQTKHSESTVPRVPDTSNSKVNTSSLVRQEGEAEL
FVPGTLYILKRNANARKEGNSVECFTLWKRHSGEHFQRILLSNNIISAHKCDSHYYALRDVLKGLPGSTDEAVF*

SlPLA1-36 supplementation

MANRSLKRFLNNIEFSSGISRKHVSNQRSVYNILNRCQSNYSNTDGHKPEEKSISNGRERSKYDIDDGDFH
DNKWKLELDWLSKAVEPAVQLCRWALSTGNGNGDKLPPTNKSLAEIFASIQRSKLGLQDWSLTDLTIGLYLIYLQ
QASTSPIEDVKGEQIYSDLIVQDLIYHTELAKGSYKDCTAALSRNCMLRESNVVKFIKNSSVLRPGYYIGIDKRKKL
VVLGIRGTHTVYDLITDIVSSSHEEITLEGYSTHFGTSEAARWFLTHEMGTIRNCLEKHKGFRLRLVGHSLGGATAS

LLAIMLRNMSARELGFSPDIVSAVGYATPPCVSRELAESCSEYVTTVVMQDDIIPRLSIASLTRLRNEILQTNCIYNSKSSRTNYFLLVAIVYGSQNDFDLSMNFEKQLMGSAKWCFAFFPPLTTPFLAYIRISSFRITLVFFATPAIYSLVRCNLSRIFHENCDAVAFVLFPSSALRGGYVLQKEDWRGVVDLFTNAKQVVSSVQDVARRLADYAKLRSQTKHSGMFMSIGMACTNSTFHYTHKSFLFVNCRGLWDVNVTCTESTVPRVPDTSNSKVNTSSLVRQEGEAELFVPGTLYILKRNANARKEGNSVECFTLWKRHSGEHFQRILLSNNIISAHKCDSHYYALRDVLKGLPGSTDEAVF

Solyc07g014730 SlPLA2-2

MVKLSLHFLAFCIIAIFTNLFNSPISIHALNVGVETNAGLSLEKECSRTCESKFCAVPPFLRYGKYCGIMYSGCPGEQPCDALDACCMKHDLCIQHKDNNYLNLECNENFLSCVAKFTKSGSPTFKENTCSITTVVRVITDVIDAAVAAGKIFKKP*

Solyc07g032220 SlPLA2-3

MLHGDKSPMGKWVASSFILTILLFFSIAESTNNSQVRCSKTCVAENCNCMSSLSFTLLNSFSHNAYLIYFFFSAIGIRYGKYCGVGWSGCPGEKPCDDLDACCKIHDECVEKNGMTNVICHEKFKRCIKKVQKSGKAGFTRDCPYDVAVPTMVQGMDMAILFSQLGNSKLEL*

SlPLA2-3 supplementation

MLHGDKSPMGKWVASSFILTILLFFSIAESTNNSQAILTFSFSIQCIFLFSFTNSSIKNSAIGIRYGKYCGVGWSGCPGEKPCDDLDACCKIHDECVEKNGAIPFNSSSNLFDLLLFQL

Solyc07g055160 SlPLA1-37

MGKEPTWHELLGSNNWEGLLEPLHLNLRRLILRCGDFCQATYDAFNNDEYSMYCGSSRYGKSSLFHKVMFKSASDYQIVSFLYATARVGAHKALFLHSLSRESWDRESNWIGFIAITNDEISEQLGRREIYIAFRGTTRNYEWVNVLGARSESAEPLICNGGSICNDDENAPRVMNGWLKIYISSDPKSPFTRLSIRAQLQVMIEDLRDRYKKENLSITFTGHSLGASLSILAAFDLVENGVTDIPVSAIVFGSPQVGNRAFNDKLKEFPNLKILHVKNKIDVITLYPSSLLGYVNSGIELVIDTRKSPCLKDSKNPSDWHNLQAMLHIIAGWNGEHGEFEMKVKRSLALVNKSSSMLKDEILIPGSWWVEKNKGVVLDEDGEWILAPPLEEDIPIPEVYSLENEAISKLLEDEITQIHEDGIEEEEHRHIAILKESRLLIHRTKAVLKQSYKEGHFVFRQYGKHGETS*

SlPLA1-37 supplementation

MGKEPTWHELLGSNNWEGLLEPLHLNLRRLILRCGDFCQATYDAFNNDEYSMYCGSSRYGKSSLFHKVMFKSASDYQIVSFLYATARVGAHKALFLHSLSRESWDRESNWIGFIAITNDEISEQLGRREIYIAFRGTTRNYEWVNVLGARSESAEPLICNGGSICNDDENAPRVMNGWLKIYISSDPKSPFTRLSIRAQLQVMIEDLRDRYKKENLSITFTGHSLGASLSILAAFDLVENGVTDIPVSAIVFGSPQVGNRAFNDKLKEFPNLKILHVKNKIDVITLYPSSLLGYVNSGIELVIDTRKSPCLKDSKNPSDWHNLQAMLHIIAGWNGEHGEFEMKVKRSLALVNKSSSMLKDEILIPGSWWVEKNKGVVLDEDGEWILAPPLEEDIPIPEYSLSPHYIELFIVYSLENEAISKLLEDEITQIHEDGIEEEEHRHIAILKESRLLIHRTKAVLKQSYKEGHFVFRQYGKHGETS

Solyc07g056250 SlPLA1-38

MDKISAEEMKNEGETTAKESGIESHPYAFHVSGPRNVSSPNWKDLINSSWRKDANYKRTVMACFIQAVYLLELDRQDNRTEQNALAPKWWIPFRYKLVETLKDERDGSIFGAILEWDRSAALADFVLMRPSGAPRGVLALRGTILKSQTMRRDIEDDLRFLAWESLKGSVRFSGVLKALKAIADKYGSNNVCIAGHSLGAGFALQVGKALAKEGIYVEAHLFNPPSVSLAMSFRNIGEKAGFAWKRIKAMLPSKADSQISCEEGGATSFPVGLKQWVPHLYINNSDYICCSYTYADGAQNDQTAANKENAKQTTNCRQAAAKLFLSSKGNQKFLEAHGLEQWWSDNLELQMAISNSKLISQQLKSLYTMPAAQLTPVKR*

Solyc08g006850 SlpPLA16

MATTKSFLILIFMILATTSSTSATLEDVVTILSIDGGGIKGIIPGVILEFLEGKLQELDRDARLADYFDVIGGTSTGGLITAMITTPNENNRPFAAANEIVPFYFQHGPHIFNSSFDGKYLLQILEDNFGETRLHQALTEVVISSFDIKRNKPVIFTKSNLTNYPELNATMYDICYSTAAAPTVFPPHYFVTNTSNGDTYEFNLVDGGVASVGDPALLSVSVATKLAEEDPAFASIRSLNLKKMLLLSLGTGTNSEFDKTYTANETAKWGYFQWKSVIPPMLDASSSYMTDYYLSTVFQALDS

QDNYLRVQENALTGTTTDMVDASVANMDLLKQVGKNLLKKPVSKDNPETYEQALTRFAQSLIDRKNLRANKASF*

SlpPLA16 supplementation

MATTKSFLILIFMILATTSSTSATLEDVVTILSIDGGGIKGIIPGVILEFLEGKLQELDRDARLADYFDVIGGTSTGGLITAMITTPNENNRPFAAANEIVPFYFQHGPHIFNSSTGTFLGPSFDGKYLLQILEDNFGETRLHQALTEVVISSFDIKRNKPVIFTKSNLTNYPELNATMYDICYSTAAAPTVFPPHYFVTNTSNGDTYEFNLVDGGVASVGDPALLSVSVATKLAEEDPAFASIRSLNLKKMLLLSLGTGTNSEFDKTYTANETAKWGYFQWKSVIPPMLDASSSYMTDYYLSTVFQALDSQDNYLRVQENALTGTTTDMVDASVANMDLLKQVGKNLLKKPVSKDNPETYEQALTRFAQSLIDRKNLRANKASF

Solyc08g006860 SlpPLA17

MATTKSFLILIFMILATTSSTFATEEMVTVLSIDGGAIRGIIPGVILRYLEAELQRIDNNTDARVADYFDLIGGTSTGGLVTAMLTTPNENNRPFAAANEIVPFYFEHGPKIFEPSGNPLFGPQYNGTYLMQVIQEKVGETFLNQTLTEVVISSFDIQTNKPVIFTKSSLAKSPELNAKMYDICYSTAAAPTYFAPLGFNTSHNGDQYRFNLVDGGVATVGDPALLSVSVATKLAEEDPAFSSIRSLNFKRMLLLSLGTGTTSDFDKTYTAEQAATWGILQWGSAIQAMTGAASSYMTDYYLSTVFQALDSQDNYLRVQDNALTGTTTAWDDASMANMLLLEQVGENLLKKQVSNDETYEQALTRFAEKLSAQKKLRENKASY*

SlpPLA17 supplementation

MVTVLSIDGGAIRGIIPGVILRYLEAELQLFRAPFLIFFEDTSSIIKIFCNSFRIPFLIFLKDNFRYRKNISSCMYRIDNNTDARVADYFDLIGGTSTGGLVTAMLTTPNENNRPFAAANEIVPFYFEHGPKIFEPSGNPLFGPQYNGTYLMQVIQEKVGETFLNQTLTEVVISSFDIQTNKPVIFTKSSLAKSPELNAKMYDICYSTAAAPTYFAPLGFNTSHNGDQYRFNLVDGGVATVGDPALLSVSVATKLAEEDPAFSSIRSLNFKRMLLLSLGTGTTSDFDKTYTAEQAATWGILQWGSAIQAMTGAASSYMTDYYLSTVFQALDSQDNYLRVQDNALTGTTTAWDDASMANMLLLEQVGENLLKKQVSNDETYEQALTRFAEKLSAQKKLRENKASY

Solyc08g007225 SlPLA1-39

MLIGATLPATNLHFFQARRASFRRYGSPLNPSANSSQSRKPQLLDNDSRGEIHPRESRRSNGSKENRGKNLLEGLNLSRIWPEHKVAEEMSPRHLNKLKKLLSSNSIEYSPRNNLGSRWKEYHGCKDWLGLIDPLNENLRRELIRYGEFIQAAYHCFHSNPATCNECDTRKVALPDKSYKVTKSLYATSSIGLPKWVDDVAPDLGWMTQRSSWIGYVAVCDDRSEIKRMGRRDIVIALRGTATCLEWGENFRDLLVQIPTKIESESESSEGQAKVECGFLSLFQTAGVNVPSLAESVVNEVQRLIEQYKGESLSITVTGHSLGAALALLVADEVSTCTPDSPPVAVFSFGGPRVGNRSFADRLNSRNVKVLRIVNNQDVITRVPGMFVSEELDKKLRESGFVSGMLNVLDKSMPWAYAHVGTELRVDTRMSPFLKPDADVACCHDLEAYLHLVDGYIASNCPFRANAKRSLAKLLSEQRSNIKMLYTSKAKGLNLNLEREHSFSTPSCLPSPSS*

SlPLA1-39 supplementation

MSPRHLNKLKKLLSSNSIEYSPRNNLGSRWKEYHGCKDWLGLIDPLNENLRRELIRYGEFIQAAYHCFHSNPATCNECDTRKVALPDKSYKVTKSLYATSSIGLPKWVDDVAPDLGWMTQRSSWIGYVAVCDDRSEIKRMGRRDIVIALRGTATCLEWGENFRDLLVQIPTKIESESESSEGQAKVECGFLSLFQTAGVNVPSLAESVVNEVQRLIEQYKGESLSITVTGHSLGAALALLVADEVSTCTPDSPPVAVFSFGGPRVGNRSFADRLNSRNVKVLRIVNNQDVITRVPGMFVSEELDKKLRESGFVSGMLNVLDKSMPWAYAHVGTELRVDTRMSPFLKPDADVACCHDLEAYLHLVDGYIASNCPFRANAKRSLAKLLSEQRSNIKMLYTSKAKGLNLNLEREHSFSTPSCLPSPSS

Solyc08g022240 SlPLA1-40

MKAIHLPLVTHLHHHDLDPNKNRVPVVYAYQDVKTSKKSNRLGEKLSNLLNIQKNDPTRSNLNDIHEDKANTPTMSPKEDISDRWCDIHGVQEWEGLLDPLHPFLRREIVKYGEFAQATYDALDIDSFSEYCGSCMYNSHKLFDKLGLNKSGYRVTKYIYAMSQIDMPQWLERSKLTYTWSKDSNWIGFVAVSDDEESRRIGRRDIVVAWRGTVTPSEWYENMQRKLESIGHMDSKVEHGFLSIYTSKCDSTRYNKSSASQQVMKELKTLVEFYKTKGEQVSLTITGHSLGGALALLNAYESAANFPKLPISVISFAAPRVGNIAFRDELYQMGVKILRVTVKQDLVPRMPGIVLNESLQKFDDLTG

TLEWIYTHAGTELKLDVRSSPYLKRGFNFIGIHMLETYLHLVDGFVSSTSTFRSNAKRDVALVNKACDMLVDELRI
PTCWYQLAHKGLECNSYGRWVRPKRHPEDIPSPTREQLL*

Solyc08g023410 SlPLA1-41

MKELKTLVEFYKTKGEQVSLTITGHSLGGALALINAYESATNFPKLPSSVISFAAPRVGNIAFRDELYQMGVK
IYVLQ*

Solyc08g078090 SlPLA1-42

MQVAATLPATGVHFFPTRRASFKCNGYSSPLKPIARASSINAQSLQTITPTTTTTEMTKKHLSNLEKLLQKE
AKPEPVIQKQGKTGENRGKQGEKTEENRGRNLLEGLNLSRIWPEMKAAEEMSPRHLIRLHRMLSSKSMEYSPR
NNLGSRWKEYHGCKDWLGLLDPLDENLRRELVRYGEFIQAAYHCFHSDPATSANENAHVARDVSLPDRSYKVT
KSLYATSSIGLPKWVDDVAPDLGWMTQRSSWIGYVAVCDDKTEIQRMGRRDIVIALRGTATCLEWGENLRDVL
VQMPGENELVDAQPKVECGFLSLYKTGGAKIPSLAESVINEVKRLIEMYKGESLSITVTGHSLGAALALLVADDIST
CSPDAPPVAVFSFGGPRVGNKGFANRLESKNVKVLRIVNKQDVITKVPGMFVSEAIDKKLRDTGASGVLNLLDN
SMPWAYSHVGTELRVDTTKSPFLKPDADVACCHDLEAYLHLVDGYLGSNESFRPNAKRSLEKLLSEQSANIKKLY
TSKGKDLSSLNLNREINFPRPSCLPSPSVLPSPSS*

Solyc08g082450 SlPLA1-43

MATSNDIVAATKGQEELSVLISEREIFDQCGPVHLTAIDWENVAHQRSVAASLVQGVYILERDKQEKRKGS
QALAPPWWRHFQFELYRVLIDDVDSCIFGAIYKFAPSKSYFGGSKDKSQRFVIAFRGTLTKGDAFSRDIQLDIHIL
RNGLHQTSRFETAIQAVRHVVATFGSSSIWLTGHSLGAAMAMLAGKTMAKTGVFLDAFLFNPPFLSAPIERIKD
QKVKHGIRFATSVITAGLAFAAKHKNVNNQSGDTFVALSAWTPCLYVNPSDPICAEYIGYFEHREKMDTMGAG
VIEKLATQHSLGGLVLNFMGKECDEPLHLIPSANLTVNLTPPSDFKGAHGIHQWWKPDLLVESKKHQFT*

SlPLA1-43 supplementation

MATSNDIVAATKVEDWGRRFVGSEWCSTFKGESQEFVPNNEVGELHLAAHFLLSGTFDGKGQEELSVLIS
EREIFDQCGPVHLTAIDWENVAHQRSVAASLVQGVYILERDKQEKRKGSQALAPPWWRHFQFELYRVLIDDVD
SCIFGAIYKFAPSKSYFGGSKDKSQRFVIAFRGTLTKGDAFSRDIQLDIHILRNGLHQTSRFETAIQAVRHVVATFGS
SSIWLTGHSLGAAMAMLAGKTMAKTGVFLDAFLFNPPFLSAPIERIKDQKVKHGIRFATSVITAGLAFAAKHKNV
NNQSGDTFVALSAWTPCLYVNPSDPICAEYIGYFEHREKMDTMGAGVIEKLATQHSLGGLVLNFMGKECDEPL
HLIPSANLTVNLTPPSDFKGAHGIHQWWKPDLLVESKKHQFT

Solyc09g056350 SlPLA1-44

MESLQRRVESWIRGQKSKMLKITWPQQWKMVVRWPWADAREQRKLMEDEFKRRKKQLEDLCHAVK
AESVADLHDILCCMVLSECVYKRPDAEMVRAVNKFKADFGGEVVSLERVQPSSDHVPHRYLLAEAGDTLFASFI
GTKQYKDVMADVNIFQGALFHEDAVEDIHGLEPIESGQVDTQRSNRESHYKISKSKTRPSNLTQKPAAHRGFM
ARAKGIPALELYRLAQKKKRRLVLCGHSLGGAVAVLATLAILRVFAASSKDNEKVQVKCITFSQPPVGNAALRDYV
NEKGWQQYFKTYCIPEDLVPRILSPAYFHHYNARPLPIPSDGGASVSMSKSSELSLLKQKIEKPKDDEREQLVLGV
GPVQNSFWRLSRLVPLEGVRKQLYRYRGKKVEPLETPTDSDSIASVNDIADTPQSLEIQEGSDGISLRLLPTDQDI
LGEGNLGKSVAESNVNNGDKRGWRRMPYLPLYVPFGQLYLLENSSVEFLSGAEYSKLTSVRSVLAEVKERFQSH
SMKSYRFRFQRIYELCMSDDTIPFLGIEQVQQFPQLQKWLGISVGGTVDLGHIVESPVIHTATSLVPLGWSGIPSG
KNTDPFKVDISGFGLHLCTLVEARVNGRWCSTSVESFPSSPVHSPDHGEQSEVQNMRVLVGGPLKRPPKHHM
VEDIPMFSSIDSSYIDTKLKQNVFKVEGRNLVLPDGLDDFVIYCTTDFSTVWKEVNLRTRRVKLIGLEGSDDDVQE
GIAGGLCYSDSTGVNLQNLNMEATHFRDDLWKGIRDLCKKTDLIILVHNLSHKIPRYNDSNALQPQPAMCLLLN
EAKSLGIPWILAITNKFSVSAHQQKVAINAVVKAYQASPSTTEVVNSCPYVTSSAAGASQSWYTEGKDPEWMF
GAQKLIFAPLELVRRPFQKKTAVLPIDGVSALCELVHRVLRSQEEAALLEFARDRLFVELARERAVEIQDAQTKVNP
LNAAAVGASLGAGLGLVLAVVMGAASALRKP*

SlPLA1-44 supplementation

MESLQRRVESWIRGQKSKMLKITWPQQWKMVVRWPWADAREQRKLMEDEFKRRKKQLEDLCHAVK

AESVADLHDILCCMVLSECVYKRPDAEMVRAVNKFKADFGGEVVSLERVQPSSDHVPHRYLLAEAGDTLFASFI
GTKQYKDVMADVNIFQGALFHEDAVEDIHGLEPIESGQVDTQRSNRESHYKISKSKTRPSNLTQKPAAHRGFM
ARAKGIPALELYRLAQKKKRRLVLCGHSLGGAVAVLATLAILRVFAASSKDNEKVQFLASYLILSSYVNEKGWQQY
FKTYCIPEDLVPRILSPAYFHHYNARPLPIPSDGGASVSMSKSSELSLLKQKIEKPKDDEREQLVLGVGPVQNSFW
RLSRLVPLEGVRKQLYRYRGKKVEPLETPTDSDSIASVNDIADTPQSLEIQEGSDGISLRLLPTDQDILGEGNLGKS
VAESNVNNGDKRGWRRMPYLPLYVPFGQAVFLEVIPLSFKMMKDVRWGDKHYLVYAKYVNSTALSPGEFICG
VPIWCRILKIDIVLGVTFSRNAIISHPPLLVKEEKPIQTGCFSFWLLLMAKTFPGENNVVMFFTLRLSSDSWSRRRT
VRSYDQIQVSVEKHLVISSIRLSFGGQSYDTCVGGRIYELCMSDDTIPFLGIEQVQQFPQLQKWLGISVGGTVDL
GHIVESPVIHTATSLVPLGWSGIPSGKNTDPFKVDISGFGLHLCTLVEARVNGRCQSLTSPHCICTSPHYIPEPPAF
CPAPMPLLVSDIVVFNSISPSICPHASLHLHLHHYHLLNVRLLEEAGED

Solyc09g065240 SlpPLA18

MDFSKISLEIFSKLEQKWLYHYEGKKTRVLSIDGGGTTGIVSGAALIHLENQICAKTGDPHVRISDFFDIVVG
TGIGAIYAAMLVVDGGDGRPLFTAKDAVKFVKENQSRLFKAKKVGVLRRKKRFSGNSMEKVLKEVFRREDGKAL
TLRDTCKPLLVPCFDLNSAAPFVFSRADAIESFSYDFDLWKVCRATSANPSMFKPFNLKSVDGKTSCLAVDGGLV
MNNPAAAAVTHVLHNKRDFPSVTGVDDLLVLSLGNGPLNSTANLKMRNDGYCSPLSVVGIVFDGVSETVDQ
MLGNAFCWNPNDYVRVQATGYASGGVGPGVEEALEERGVESLPFGGKRLLTETNGQRIGGLVQRLVATGRSSV
PPSPCKDVAVSPLRNGR*

Solyc09g065890 SlPLA1-45

MLDPMDPLLRSELIRYGEMAQACYDAFDFDPYSKYWGVANFQDVSFFDGLGMAEYGYDITRYLYATSNI
NLPNFFKQSRWPKIWSKNANWIGYVAVSNDETTKRLGRRDITIAWRGTVTRLEWIADLMDYLRPISSDNIPCP
DPNVKVESGFLDLYTDKDESCRYCKFSAREQILTEVKRLIEMYPDEENEHNGYRAQLR*

SlPLA1-45 supplementation

MASLTKNSILTFQTCTPHKHQVKSSTKTRSLSSRTSQSFTSIINEKEKTNLSIDQLEESENTSNRTNIYEAEVV
DRWLEIHGQDDWVGMLDPMDPLLRSELIRYGEMAQACYDAFDFDPYSKYWGVANFQDVSFFDGLGMAEY
GYDITRYLYATSNINLPNFFKQSRWPKIWSKNANWIGYVAVSNDETTKRLGRRDITIAWRGTVTRLEWIADLMD
YLRPISSDNIPCPDPNVKVESGFLDLYTDKDESCRYCKFSAREQILTEVKRLIEMYPDEENEHNGYRAQLSGNGVK
CEGGYKWCANMCIFISGPRVGNVRFKERIEKLGVKVLRVVNVHDIVPKSPGLVLNEHSPSMVMKICEKLPWSY
SHVGVELALDHKNSPFLKPTSDLVCAHNLEAHLHLLDGYHGKGRRFVLEKGRDIALVNKACDFLKDHYCVPPN
WRQDENKGMIRDKDGRWVQPERPRLDDHTHDIHHHIKHLGL

Solyc09g091050 SlPLA1-46

MATLQTHLQFPICSSPRLFHFKNPNSVSFSKKLFFSRKVNGLFSYSKFGAKDSFFCCSQTSGEILPLSSAQKE
KETSERPPFDINLAVILAGFAFEAYTSPPDNVGKLEVDAANCKTIFLSESFVREIYDGQLFIKLKKGLNLPAMDLWG
TSDPYVVLQLDSQVVKSKVKWGTKEPMWNEEFALNIKQPPLYDLQIAAWDANLVTPHKRMGNAAVNLEHLC
DGDSHKLLVDLDGMGGGGKIEIEIKYKSFEKIEEEKKWWNIPIITEFLRKNGFESALKTILGSETVQARQFVQFAF
GQMKLLNDAYNDSNSSSSPVLESDVLPESQQSSNLDDSSMPPASEISNNLKDTKVDGEVKLNRDGSDVTDEHD
SPGTKILESFQSDKHFWKNFADTVNQKVVQRLGLPAPEKIKWDNLDLLNKIGLQSRKDADASYVESGLATPDKR
ENVNGSASTESPILNNIQSSLPDIKKVTQDLLRQTDTILGALMVLNATVSQFNKGAGLFGKGDAKEDSSTGLEND
ILLYPMNKDGIVLDEKKAEEMKSLFSTAETAMEAWALLATSLGHPTFIKSEFDKLCFLDNESTDTEVALWRDSARK
RLVVAFRGTEQTKWKDLVTDLMLVPAGLNPERIGGDFKEEVQVHSGFLSAYDSVRIRLISLIKKAIGYQDDDLDTP
NKWHVYVTGHSLGGALATLLALELSSSQLAKRGAIRVTMYNFGSPRVGNKKFAEVYNEKVKDSWRVVNHRDII
PTVPRLMGYCHVAQPVYLAAGDPQNTMDNVELLEDGYQGDVIGEATPDVIVSEFMKGEKNLLRKY*

SlPLA1-46 supplementation

MATLQTHLQFPICSSPRLFHFKNPNSVSFSKKLFFSRKVNGLFSYSKFGAKDSFFCCSQTSGEILPLSSAQKE
KETSERPPFDINLAVILAGFAFEAYTSPPVNSQSLYVHFYVFLCSVGWLSNLLRDSIFYPQMVIKQLNCFPSLIQFAL

FDIACQDNVGKLEVDAANCKTIFLSESFVREIYDGQLFIKLKKGLNLPAMDLWGTSDPYVVLQLDSQVVKSKVK
WGTKEPMWNEEFALNIKQPPLYDLQIAAWDANLVTPHKRMGNAAVNLEHLCDGDSHKLLVDLDGMGGGGK
IEIEIKYKSFEKIEEEKKWWNIPIITEFLRKNGFESALKTILGSETVQARQFVQFAFGQMKLLNDAYNDSNSSSSPVL
ESDVLPESQQSSNLDDSSMPPASEISNNLKDTKVDGEVKLNRDGSDVTDEHDSPGTKILESFQSDKHFWKNFA
DTVNQKVVQRLGLPAPEKIKWDNLDLLNKIGLQSRKDADASYVESGLATPDKRENVNGSASTESPILNNIQSSLP
DIKKVTQDLLRQTDTILGALMVLNATVSQFNKGAGLFGKGDAKEDSSTGLENDILLYPMNKDGIVLDEKKAEEM
KSLFSTAETAMEAWALLATSLGHPTFIKSEFDKLCFLDNESTDTEASSYVAGYEFLVSNVALWRDSARKRLVVAFR
GTEQTKWKDLVTDLMLVPAGLNPERIGGDFKEEVQVHSGFLSAYDSVRIRLISLIKKAIGYQDDDLDTPNKWHV
YVTGHSLGGALATLLALELSSSQLAKRGAIRVTMYNFGSPRVGNKKFAEVYNEKVKDSWRVVNHRDIIPTVPRL
MGYCHVAQPVYLAAGDPQNTMDNVELLEDGYQGDVIGEATPDVIVSEFVEAMLLGVLCWILQKLCIVRGSETA
IRDGSALMQHMEDFYYITLLENVRSNYRTVPRPQLTEEKNISIG

Solyc09g098450 SlPLA1-47

MSPLLPLLPNLRNRSKGFGLGLKFPNSFRKMMPLPAPELKRLRRSAVVLGVSNALIIIMGILIVIVAHSTCGE
NDGTSPVMVMIMVSFIRIGAMIGTGIAQQHTASSILTSQTDLPDSQIAIRQQRRRKYRRLLLWTRIASVITIMQLL
GAVFLLFTVTNLLHHDTASNNCLRGILSNGSKWQRNMLILFMVIISYVAPVQCFAGADVLRWRSFYATEDNAW
RAHYREVFDHGIREALCCLGRVKYLTVLEEDEVCSVAQLLGDLVTYRASGTGHLELLAGLALLQNSSSFSKSYEESR
VVPIERMRKAAFYHPFAEAAYTGLLLDIGRNPVLFSCSWLYRQGILAPWLWNRRPLLEGDNWWRGHAAAFLK
HVHLSAHMLRKGRVNQGKCKAAYFIVVLHNVKSVVIAVRGTETPEDLITDGLGRECCLTEEELDSLLNGNHCDP
YLHQRVVSSTPHYAHSGVVEAARDLYRQVDGNCGDGGFLASLLGVGCECEGYGVRIVGHSLGGAIAAVLGMKL
RKRYPDLHVYTYGALPCVGLVVADACSEFITSIVNNDEFSARLSVASIMRLQAAALKALSEDGTIDITTILKLAQHF
TSLTVCQKSMNDGESSVNSLTAMSSCTNQINHGQLENGLAKREAGSSVLHDIDTDFRCDDEIVSTESSNHFSSP
FNCSTSDSSPFSDPLTEFMEAVPSSENKSSLSIPELYLPGLVIHIVPQKDGLHKPLWKLWKSWERRCRFRAYVAKR
EAFKDIIVSPYMFLDHLPWRCQTALENILKTGQLKTPDDASEIV*

SlPLA1-47 supplementation

MIVMNARSALNIGFVFYFLDLEVFTLKTSSSLSISKNSSSFSKSYEESRVVPIERMRKAAFYHPFAEAAYTGLL
LDIGRNPVLFSCSWLYRQGILAPWLWNSCLHVTRRSQVRSMETTSCRNAGRPLLEGDNWWRGHAAAFLKHV
HLSAHMLRKGRVNQGKCKAAYFIVVLHNVKSVVIAVRGTETPEDLITDGLGRECCLTEEELDSLLNGNHCDPYLH
QRVVSSTPHYAHSGVVEAARDLYRQVDGNCGDVSARVCFVLTENLRKRRITYVSWSFMCGFLASLLGVGCECE
GYGVRIVGHSLGGAIAAVLGMKLRKRYPDLHVYTYGALPCVGLVVADACSEFITSIVNNDEFSARLSVASIMRLQ
AAALKALSEDGTIDITTILKLAQHFTSLTVCQKSMNDGESSVNSLTAMSSCTNQINHGQLENGLAKREAGSSVLH
DIDTDFRCDDEIVSTESSNHFSSPFNCSTSDSSPFSDPLTEFMEAVPSSENKSSLSIPELYLPGLVIHIVPQKDGLHK
PLWKLWKSWERRCRFRAYVAKREAFKDIIVSPYMFLDHLPWRRNTIIYFFRCQTALENILKTGQLKTPDDASEITL
FMDQVSPDGQNGPFSRSTSSGAAVRKTLAMESVGLADQNGPFQKLNEPQAENSETLRVAPPTPYEGIRKGFA

Solyc09g098460 SlPLA1-48

MLRLPNIPNLNFPTFYEEFLPKPCMVRPNEGISIAPLTLQQLRVSPKLSSTKNIASDDIAMDRCRCSSGRLLS
SMWREVQGSKNWENLVDPLDSLLRSEIIRYGEFVAACYDAFDLDTNSKRYLNCKYGKSRMLSEVGLGESGYQV
TKYIYATPDLIIGSNNISIGSSCGKWIGYIAVSNDEETKRLGRRDVLVTFRGTVTSPEWIANLMSSLTPARLDPHNP
RPQVKVEAGFLSLYTSNEDKKFGLGSCREQLLSEIGRLVNKYYKQEDMSITIAGHSMGSALALLLAYDIAELGLNT
HHHHHHMTTVTVFSFGGPRVGNSGFKERCEELGVKVLRITNVNDPITKLPGVFLNENSRVLGGKYEVPWSCSC
YAHVGVEILLDFFKMHNPSCVHDLGTYLNLLKCPKRCLQVQREEGIHHFINKAKEFVISGLNFNPPLQWKNVAM
NMVNMVQSQRT*

Solyc10g038170 SlPLA1-49

MPSFSGQSPEKIGKNWMEYQGIKNWEGLLDPLDDNLRGEIIRYGHFVEAAYRACNFDPSSPSYAMCKYS
RKKLFHLSGFSGTGYRISKYLKATSGINLPNWVDKAPKWMSKQSSWIGYVAICHDQREIARLGRRDVVIALRGTA

TCLEWLENLRATLTPLPNIKHTICCPMVESGFLSLYTSKIDAQQSLQDMVREEIDRIKKLYDGETLSFTIAGHSLGA
ALATLTAYDIKQFFRDIPLVTVMSFGGPRVGNHSFRYHLDKQDTKILRIVNSDDLITKIPGFVIDNNDDDDKYVEK
SDHWMKRLVEDSQWVYADVGCELRLSSSGSPHFNGINIATCHELNTYLHLVNSFVSSSCPVRATAKKIMHKSN
NNVKCT*

SlPLA1-49 supplementation

MKLSFKPSPSCTSKQNLQQHTLIQCVTITKPANKFTRNINLTKNIKNIILGWITNSDEKTSSTSSWTMPSFS
GQSPEKIGKNWMEYQGIKNWEGLLDPLDDNLRGEIIRYGHFVEAAYRACNFDPSSPSYAMCKYSRKKLFHLSGF
SGTGYRISKYLKATSGINLPNWVDKAPKWMSKQSSWIGYVAICHDQREIARLGRRDVVIALRGTATCLEWLENL
RATLTPLPNIKHTICCPMVESGFLSLYTSKIDAQQSLQDMVREEIDRIKKLYDGETLSFTIAGHSLGAALATLTAYDIK
QFFRDIPLVTVMSFGGPRVGNHSFRYHLDKQDTKILRIVNSDDLITKIPGFVIDNNDDDDKYVEKSDHWMKRLV
EDSQWVYADVGCELRLSSSGSPHFNGINIATCHELNTYLHLVNSFVSSSCPVRATAKKIMHKSNNNVKCT

Solyc10g078530 SlpPLA19

MAANSVNTISMLDSNMEVDKLTNEIFSILENKFLFGYDDPKKSVTGNEENLSARFAGNKNVGAGKVRILSI
DAGGSTDGVLAAKSLTHLESTLRRKSGKSDAHIADFFDVVAGSGTGGVLAGLLFTRGADGVPMFTTDEALRFIV
ENGEKISRSSKTGFFRQVSRPAKVFKKVFGDLTLKDTMKAVLIPCYDLKTGAPFVFSRADAWEMDGCDFAMSD
VCGATMADRAVDLKSIDGRSKITAVGGGIAMTNPTAAAITHVLNNKQEFPFANGVEDLLVVSLGNGDSDSGTG
NVMSSPAAFVKIAGDGTADMVDQAVSMAFGQTRNNNYVRIQGNGIVGKKYQLIKDENMNKCEKMKKMVVI
AEEMLGQKNVECVLFQGKKLVENSNLDKLKTIASELIKEQERRKTSILPPVVLKHASPSPRTSSVTTLSSDSSC*

Solyc10g079410 SlpPLA20

MDISNEARVEFFSIGPSSIVGRTIAFRVLFCKSISRLRRSIFHFMMYYLYKIKNCLSHYLTPLIKWFHPRNPQG
ILVLVTLLAFLLRRYTYVKIRADMVYKRKFWRNMTKSALTYEEWAHAAKMLEKDTPKMNEAEFYDEELVVNKLQ
ELQHRRNEGSLRDIMFFMRADLVRNLGNMCNPQLHKGRLHVPKLIKEYIDEVSTQLIMVCDSDSDEILLEEKLA
FMHETRHAFGRTALLLSGGASLGAFHVGVVKTLVEHKLMPRIIAGSSVGSIMCSVVATRSWPELQSFFEDSWH
VLQPFFEQMGGILTVFRRIMRQGAVHEIRQLQVMLRHLTNNLTFQEAYDMTGRVLGITVCSPRKHEPPRCLNYLT
SPHVVIWSAVTASCAFPGLFEAQELMAKDRSGNLVPYHPPFHLEPDQAAASGSSARRWRDGSLEVDLPMMQ
LKELFNVNHFIVSQANPHIAPLLRIKEFVRAYGGNFAAKLAHLSEMEVKHRCNQVLELGFPLRGLAKLFAQDWE
GDVTVVMPATLAQYLKIIQNPSTLEVQKAANQGRRCTWEKLSAIKANCGIELALDECVAILNHMRRLKRSAERA
AASSQGMSSSTVKLNASRRIPSWNCIARENSTGSLEEEFHADASSSLHHHNAGRNWRCNNKNTALDHHGSDS
ESESADNNSWTRSGGPLMRTTSADKFIDYVQNLEMHPSQRSSRGLSVDLNNVVVREPLSPSPRVTTPDRRSDT
EFDQRDIRIIVAEGDLLQSERTNNGIVFNVVRRGDLTPSNRSLDSENNSCFHDPVAECVQLENPDKDMDISSASE
DGETENAVLNVVTENQII*

Solyc10g079770 SlpPLA21

MDISNEATIDFFAIGPSTILGRTIAFRVLFCKSITQLRHRLFHFLMYYLYKFKSGISYYVTPLISWLHPRNPQGI
LALVTLLAFLLRRYTNVKVKAEMAYRRKFWRNMMRSALTYEEWAHAAKMLDKETPKLNEADLYDEELVRNKL
QELRHRRQEGSLRDIIFCMRADLVRNLGNMCNSELHKGRLHVPRLIKEYIDEVSTQLKMVCDSDSEELLLEEKLA
FMHETRHAFGRTALLLSGGASLGAFHVGVVKTLVEHKLLPRIIAGSSVGSIMCSIVATRSWPELQSFFEDSWHSL
QFFDQLGGIFTIFRRVMTQGAVHEIRQLQVLLRNLTNNLTFQEAYDMTGRVLGITVCSPRKHEPPRCLNYLTSPH
IVIWSAVTASCAFPGLFEAQELMAKDRSGDLVPYHPPFHLGPDDTSGASSRRWRDGSLEVDLPMMQLKELFN
VNHFIVSQANPHIAPLLRIKEFVRAYGGNFAAKLAQLAEMEVKHRCHQVLELGFPLGGIAKLFAQDWEGDVTV
VMPATLAQYSKIIQNPSTLELQKAANQGRRCTWEKLSAIKANCGIELALDECVAILNHMRRLKRSAERAAAASH
GLSSTVRFNASRRIPSWNCIARENSTGSLEDFLADVAASHHQGGSGSGAHTTRNWRTHRSAHDGSDSESENV
DLNSWTRSGGPLMRTTSADKFIDFVQNLEIGSRLNKGLTIDLNNLVPQMAGRDLFSPSPRVSTPDRTSDTEFDQ
RDFSIRVPAGSSSIMVGEGDLLQPERTNNGIVFNVDTVAECVQLDSPEKEMDISSVSEDGEDYVEQESGKINEV
DSVHSGDNRSTIDDGEDKQVIDKQVIDH*

SlpPLA21 supplementation

MDISNEATIDFFAIGPSTILGRTIAFRVLFCKSITQLRHRLFHFLMYYLYKFKSGISYYVTPLISWLHPRNPQGI LALVTLLAFLLRRYTNVKVKAEMAYRRKFWRNMMRSALTYEEWAHAAKMLDKETPKLNEADLYDEELVRNKL QELRHRRQEGSLRDIIFCMRADLVRNLGNMCNSELHKGRLHVPRLIKEYIDEVSTQLKMVCDSDSEELLLEEKLA FMHETRHAFGRTALLLSGGASLGAFHVGVVKTLVEHKLLPRIIAGSSVGSIMCSIVATRSWPELQSFFEDSWHSL QFFDQLGGIFTIFRRVMTQGAVHEIRQLQVLLRNLTNNLTFQEAYDMTGRVLGITVCSPRKHEPPRCLNYLTSPH IVIWSAVTASCAFPGLFEAQELMAKDRSGDLVPYHPPFHLGPDDTSGASSRRWRDGSLEVDLPMMQLKELFN VNHFIVSQANPHIAPLLRIKEFVRAYGGNFAAKLAQLAEMEVKHRCHQVLELGFPLGGIAKLFAQDWEGDVTV VMPATLAQYSKIIQNPSTLELQKAANQGRRCTWEKLSAIKANCGIELALDECVAILNHMRRLKRSAERAAAASH GLSSTVRFNASRRIPSWNCIARENSTGSLEDFLADVAASHHQGGSGSGAHTTRNWRTHRSAHDGSDSESENV DLNSWTRSGGPLMRTTSADKFIDFVQNLEIGSRLNKGLTIDLNNLVPQMAGRDLFSPSPRVSTPDRTSDTEFDQ RDFSIRVPAGSSSIMVGEGDLLQPERTNNGIVFNVVRKGDVTPSNRSLDSENNSSVQDTVAECVQLDSPEKEM DISSVSEDGEDYVEQESGKINEVDSVHSGDNRSTIDDGEDKQVIDKQVIDH

Solyc10g080690 SlpPLA22

MAAAAISTISMIDSNMEVDKLTYEIFSILENKFLFGYDSDPKLSPACRENRNFSTPFAGNKNVPAGKIRILSID GGGSTNGILAAKSLTHLETTLRRKTGKKNTHIADFFDVVAGSGTGGILATLLFTRGKDGVPLFTAEEALKFLIENNR KISRSSSNGVFRHVFRPPVKVFGKVFGDLTLKETVKAVLIPCYDLTTRSPFLFSRADALEMDGCDFKLADVCGATI ADRTVEVKSVDGKRKITAVGGGVTMNNPTAAAITHVLNNKQEFPFANSVEDLLVISLGNGESDSGTGNMTSSP AALVKIAGDGAADMVDQAVSMAFGEFRNNNYVRVQGNEIIVGKKHMIKDEKKRKSIAIAEEMLKQKNVESILF QGKKLMEKTNLEKLEIFAGELIKEEEMRKNSILSPVVLKQSSSSPRTSSATSLSTISSC*

Solyc11g011120 SlPLA1-50

MSVACGFECVVVLGCMRWVWKRCTYIGNDDSATWPPATYDEFEPIPRLCRTILAVYEDDLHNPKFPPEG GYRLNADWVVKRVTYRDTLGNAPPYLIYLDHEHHEIVVAIRGLNLVKESDYKVLMDNKLGKQMFDGGYVHHG LLKAAIWMLNKESETLKRLWVENGKSYRMIFVGHSLGSGVASLLTIIAANHGDRLGGIPRSSLRCYAVAPARCMS LNLAVKYADVIHSVVLQDDFLPRTPTPLEDIFKSVFCLPCLLFLVCLRDTFIPEGRKLRDPRRLYAPGRMYHVVERK FCRCGRFPPDVRTAIPVDGRFEHIVLSCNATSDHGIIWIQRESEKALARLKEATSAEAPTAPPPVQRIERQHTLEKE HKKALERAVTLNIPHAVPTDTDEELSVHKEDDSGRGVTETAFLIEDASTSTSHSTDARTNWNDVVEKLFAQDET GKLTLNKEASCSTE*

Solyc11g065530 SlPLA1-51

MSSFQLSLTNPKHKRNHTCLEVFPLWNTFKSNLSFPNKRLSPSLSLKTSSCLSNLEDNNTIELQQYDEEEKK PLHEMWREIQGCNNWKGLLDPMDCHLRKEIIRYGEFAQSCYDSFDYDPHSKYCGTCKYQPSQFFDKINMLKK GYEMKRYLYATSNINLPNFFQKSKMRNVWSQHANWMGYVAVATDPEEIKRLGRRDIVVAWRGTVTYLEWIH DLQDILHPAHFRDDPNIKIETGFFDMYTKKENNCHYASFSAREQILAEINRLIEKYQGEELSITITGHSLGAALALLS AYDIAEMKLNILHNGKSITKIIPITVFSFAGPRVGNLKFKERCEELGIKVLRVVNVHDKVPKVPGIIANEKFQFQKQ LEEKFSFAWSYAHVGAELALDHHRSPFLKPNSDLGSAHNLEAHLHLVDGYHGKVRAFRSATSRDVALVNKDSSF LKEEYGVPPFWWQDENKGMVRTSDGQWVLPERPIIEAHPPDTAHHFQQVLKLARARLNLP*

Solyc12g010910 SlPLA1-52

MEKEATWHELLGSKDWDGLLQPLNLPLRRLILRCGDFCQATYDAFNNDQNSKYCGTSRYGKSSFFDKVM LESSTDYKIYCFLYATAKIGALEAIFLHSLSRESWDRESNWIGYIAVTTDEVSRKLGRREVYVVFRGTSRNYEWVNV LGARPDSADSLLHPKSLQKGINNKNDEDEDEDEDEIKVMDGWLKIYVSSNPKSSFTRLSAREQLQAKIEKLRNEY KDENLSITFTGHSLGASLAVLASFDVVENGVPVDIPVSAIVFGSPQVGNKAFNERIKKFSNLNILHVKNKIDLITLY PSALFGYVNSGIELVIDSRKSPSLKDSKDMGDWHNLQGMLHVVAGWNGEDKKFELKVKRSVALVNKSSSFLKD DYLIPGSWWIEKNRGMVFDKNGEWILAPPSDEDLPVPEY*

Solyc12g036490 SlPLA1-53

MVAEKWEELSKKNKWEGLLNPLDVDLRKDIIQYGELAHVTYDTFITEKASKNAGYRATKYFYGTSCIPLPN AFITKSLLREAWGKESNFIGYVVVATDEGKVSLGRRDIVIAWRGTIQTLEWVNDLQFLLIPGPQVFGKGGLAQPL VHHGFYNIYTSESVRSKFNQASARDQEYKDDEVSITVAGHSLGASLATLHAVDIAYNGINKQVNRDLALVNKRW DILKAEYLVPGAWWVEKHNGMVKQQDGKWILKDYEEYEF*

Solyc12g055730 SlPLA1-54

MATSNIDSFLIVKPENGGTWDLVGPLLGGLGEINAKFLEYSCADDGNGDCDGGEEVDDHRWVIVVSILV RKILKVFKKPMEWSGYFLEFFINIFSLNGNFRGLFHNILHGRLVIPQRGTANFISIIGHIDGRIKLPYNTEILTKESSRL IEKNEQLGMRHRDLMDLCMMSSKLVYENEIFVEDVVNHQWKMHFVDFYHCWNDYQKQNSTEVFILCDKSK DANLILISFRGTAPFSADDWITDFDYSWYEIPKIGKLHMGFLEALGLGSRVNVSTFHEHLVDKNVDVTIAHPERIT AYYTVRSKLKSLLKEHENARFVVTGHSLGGALAILFPTMLVVHEEMDVMKRLSRVYTYGQPRVGDRQLGRFME EHLEHPVPKYFRIVYNNDIVPRLPYDDKTFLYKHFGVCMYYNSSYVEEEVHEEPNMNFFGLRYVLPMYLNAVW EFIRSLFMCYIYGPEYKESWESIVFRMVGMFLPGISAHSPVNYVNSVRLGKKKSSDVSESVD*

Solyc12g088800 SlPLA1-55

MANNNDEEFCKDYFELKAQEASYFDFIRIFYSSNLDKRNFFDVSIGVASTIRGFRRRWLIFISIVLQRLFFWF KNPMENLGSFMELLQNYPSFNGGFIQLFLNIFQGKVVRPEKSSEKFKSMIGNLDLRVELDKKIKIGDIRYNRHVSI MAAKLSYENEALNKTVIQKHWQMHFLGLYNFWNAYEEQYSTQAIMFQDKIEDPNLIVVAFRGTSPFNANAWI TDIDLSWYELEGLGKIHAGFMKALGLQKPIGWPKQINQDQNNSNSKEFAYYKIREELKKILSKNEKAKFIVTGHSL GGALAILFASILILHEEEWLLDKLEGVYTFGQPRVGDEQFGRFMMEKLKKFDVKYYRYVYCNDMVPRLPYDDKT LFFKHFGSCLYYNSLYCGKVLEEEPNKNYFSLLWVLPKVLNGVFELIRSFILPWIKGNDYKQSWSEMIFRMVGLIIP GLSAHGPVDYVNLTRLGTNLHLPQSQGGLKQD*

Solyc12g098730 SlPLA1-56

MLPNMKIFTFSSEFLPKSCLVKGNEVKLSTSRLTLLACTMPTTTFSHSNSSSNINTTNTNNNKLATMWREI QGSRNWENLVNPLDSLLQEEIIRYGEFVVACYNAFDLDPNSKRYLNCKYGKNSMLSKVGLGKSGYEITKYIYATT NINVLSIGQNSSPSSSGRWIGYVAVSNDEETKRLGRRDVLITFRGTVTSPEWVANLMSTLSPARLDPSNLRPEVK VEAGFLSLYTSKEGERFGLRSCREQLLSEIGRVTNMYKNEEMSITIGGHSMGSALALLLAYDIAELGLNKHATTTR RDQLYKSSVSEPVSERVSEPESSNISVTVFSFGGPRVGNSGFKERCEELGVKVLRIVNVNDPITKLPGVLLNENFR VFGGRYEVPWSYSCYTHIGVEILLDFFNMQNPSCVHDLGTYLNLIKSRRHHHHKSLQVQRGEHEDIFFNIAKEFF LLMLYYGEFLPLIIW*

SlPLA1-56 supplementation

MKIFTFSSEFLPKSCLVKGNEVKLSTSRLTLLACTMPTTTFSHSNSSSNINTTNTNNNKLATMWREIQGSR NWENLVNPLDSLLQEEIIRYGEFVVACYNAFDLDPNSKRYLNCKYGKNSMLSKVGLGKSGYEITKYIYATTNINVL SIGQNSSPSSSGRWIGYVAVSNDEETKRLGRRDVLITFRGTVTSPEWVANLMSTLSPARLDPSNLRPEVKVEAGF LSLYTSKEGERFGLRSCREQLLSEIGRVTNMYKNEEMSITIGGHSMGSALALLLAYDIAELGLNKHATTTRRDQLY KSSVSEPVSERVSEPESSNISVTVFSFGGPRVGNSGFKERCEELGVKVLRIVNVNDPITKLPGVLLNENFRVFGGR YEVPWSYSCYTHIGVEILLDFFNMQNPSCVHDLGTYLNLIKSRRHHHHKSLQVQRGEHEDIFFNIAKEFFLLMLY YGEFLPLIIW
